# Cross‐Organ Multi‐Omics Profiling of Systemic Alterations in 5xFAD Mice and Changes Associated with Huanglian Jiedu Decoction Treatment

**DOI:** 10.1002/advs.78442

**Published:** 2026-10-31

**Authors:** Wenya Gao, Jing Gao, Xiaohui Su, Xiangying Kong, Xiaolu Wei, Tunan Ding, Tiantian Guo, Shuangrong Gao, Yanyan Zhou, Junyi Zhou, Haiyu Zhao

**Affiliations:** ^1^ Institute of Chinese Materia Medica China Academy of Chinese Medical Sciences Beijing China; ^2^ Beijing Academy of Traditional Chinese Medicine Beijing University of Chinese Medicine Beijing China

**Keywords:** Alzheimer's disease, cross‐organ, energy metabolism, Huanglian Jiedu decoction, metabolomics, proteomics, transcriptomics

## Abstract

Alzheimer's disease (AD) is increasingly recognized as a systemic disorder involving metabolic abnormalities beyond the brain, but links between hepatic alterations and neuroinflammation remain unclear. We integrated behavioral, histopathological, transcriptomic, proteomic, phosphoproteomic, and metabolomic analyses in male mice assigned to WT (*n* = 15), AD (*n* = 16), high‐dose HLJDD (*n* = 16), low‐dose HLJDD (*n* = 11), and donepezil (*n* = 10) groups and treated for 10 weeks. Longitudinal behavioral data were analyzed using repeated‐measures models, and omics *p*‐values were adjusted using the Benjamini–Hochberg FDR procedure. Cognitive impairment, glial activation, and neuroinflammation were accompanied by hepatic metabolic disturbances and alterations in insulin signaling and energy metabolism, implicating PPAR and PI3K/Akt‐related processes. HLJDD, particularly at the high dose, improved behavioral and neuropathological outcomes and shifted abnormalities in TCA‐cycle metabolites, amino acid neurotransmitters, and oxidative balance toward WT levels. To assess hepatocyte contributions to microglial inflammation, we established an AML12–BV2 conditioned‐medium model with hepatocyte PPAR‐γ knockdown. Conditioned medium from LPS/HLJDD‐cotreated AML12 cells elicited lower inflammatory responses in BV2 cells than medium from LPS‐treated hepatocytes. PPAR‐γ knockdown attenuated this effect, supporting a hepatocyte PPAR‐γ‐dependent contribution to microglial regulation and further investigation of hepatic involvement in HLJDD‐associated neuroprotection.

## Graphical abstract

Cross‐organ multi‐omics reveals coordinated inflammatory and metabolic disturbances in 5xFAD mice. Huanglian Jiedu Decoction improves cognition and alleviates brain and liver abnormalities. Complementary hepatocyte–microglia conditioned‐medium experiments suggest PPAR‐γ involvement in treatment‐associated changes in microglial inflammation, providing functional evidence of hepatocyte–microglia communication and a basis for further investigation of liver–brain interactions in Alzheimer's disease.

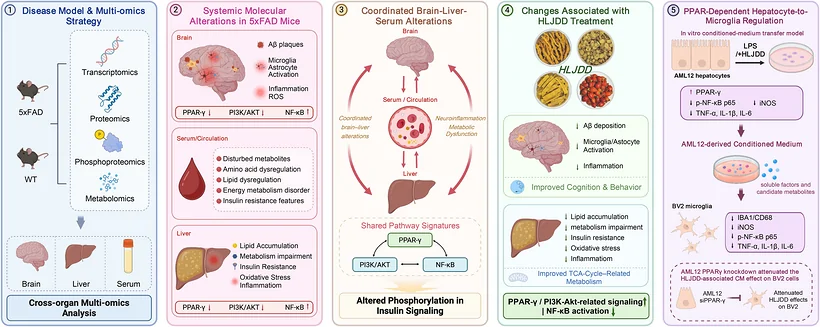

**Abbreviations**
AD: Alzheimer's disease
ANOVA: analysis of variance
ApoE: Apolipoprotein E
Aβ: Beta‐amyloid protein
BCAA: branched‐chain amino acids
CE: collision energy
DEGs: differentially expressed genes
DEMs: differentially expressed metabolites
DEPs: differentially expressed proteins
DP: declustering potential
DPPs: differentially phosphorylated proteins
ESI: electrospray ionization
FC: fold change
GO: gene ontology
GSEA: gene set enrichment analysis
H&E: hematoxylin‐eosin
HILIC: hydrophilic interaction chromatography
HLJDD: Huanglian Jiedu Decoction
HMDB: Human Metabolome Database
IL‐6: Interleukin‐6
IR: insulin resistance
KEGG: kyoto encyclopedia of genes and genomes
LC‐MS: liquid chromatography‐mass spectrometry
NF‐κB: Nuclear factor kappa‐B
NO: Nitric oxide
NOS: Nitric oxide synthase
OPLS‐DA: orthogonal partial least squares discriminant analysis
OXPHOS: oxidative phosphorylation
PCA: principal component analysis
PPAR: peroxisome proliferator‐activated receptor
QC: quality control
ROS: reactive oxygen species
RSD: relative standard deviation
SEM: standard error of the mean
T2DM: Type 2 diabetes mellitus
TCA: tricarboxylic acid cycle
TGF‐β1: Transforming growth factor β1
TNF‐α: Tumor necrosis factor alpha
VIP: variable importance in projection
WT: wild‐type

## 1 Introduction

Alzheimer's disease (AD) is a progressive neurodegenerative disorder primarily characterized by declining cognitive function and behavioral impairments, representing the most prevalent form of dementia [1, 2]. As global population aging accelerates, the rising incidence of AD imposes an escalating socio‐economic burden. The etiology and pathogenesis of AD involve a complex interplay of multifaceted factors. Although multiple pathogenic processes—including amyloid‐β (Aβ) aggregation, tau hyperphosphorylation, neuroinflammation, ferroptosis, and oxidative stress—have been implicated in AD, the mechanisms underlying disease initiation and progression remain incompletely understood [3]. Current therapeutic options remain limited, and many available treatments primarily provide a symptomatic benefit or modestly modify disease progression. Aging is a major risk factor for AD, often accompanied by declining metabolic rates. In the brains of AD patients, impaired glucose uptake is one of the most well‐characterized defects [4]. Notably, previous studies have reported alterations in brain energy metabolism before the emergence of typical AD pathological features, suggesting that metabolic dysregulation may contribute to the early stages of neurodegeneration [5]. Consequently, metabolic dysregulation is increasingly recognized as an important contributor to neurodegenerative processes [6]. Accumulating evidence has also linked AD to brain insulin resistance, a concept sometimes referred to as “type 3 diabetes”, and has suggested coordinated inflammatory and metabolic interactions between peripheral organs and the central nervous system (CNS) [7].

Previous studies of AD have mostly focused on CNS‐intrinsic mechanisms. However, recent findings highlight the vital contributions of peripheral organs in sustaining normal brain function and influencing the pathogenesis of brain diseases [8]. Although AD primarily affects the brain, its onset and progression are closely linked to dysfunction in peripheral organs, suggesting new therapeutic opportunities. As the body's metabolic hub, the liver is essential for systemic glucose and lipid homeostasis and has recently emerged as an important contributor to AD progression, supporting the view that AD is a systemic disorder shaped by gene environment interactions.

Recent studies have implicated hepatic dysfunction in AD progression, noting that patients with impaired liver function exhibit significantly elevated plasma Aβ levels [9]. The liver is a major organ responsible for clearing circulating metabolites from the blood [10]. Liver dysfunction has been associated with cognitive decline [11]. Researchers have found that the liver physiologically eliminates Aβ from the blood and regulates the level of Aβ in the brain [12]. Furthermore, aging‐related increases in liver‐derived ApoE4 infiltration into the brain position the liver as a critical therapeutic target. Chronic hepatic inflammation and oxidative stress further disrupt the blood‐brain barrier (BBB), facilitating the influx of neurotoxic metabolites into the brain [10]. The liver–brain axis may form a vicious cycle in AD, in which peripheral metabolic dysfunction worsens cerebral amyloid deposition, neuroinflammation, and mitochondrial failure. By integrating multi‐organ transcriptomic, proteomic, phosphoproteomic, and metabolomic data, this study shows that coordinated neuroinflammatory and metabolic disturbances are associated with the systemic pathological phenotype of 5xFAD mice.

Traditional Chinese Medicine (TCM) offers a holistic strategy for AD by restoring systemic balance through multi‐component, multi‐target regulation rather than symptom control alone. In TCM theory, although AD manifests in the brain, its pathogenesis is closely linked to Zang‐fu dysfunction, with deficiency, turbidity, stagnation, and toxin jointly contributing to cerebral malnutrition and cognitive decline. Based on this framework, Huanglian Jiedu Decoction (HLJDD), a classic heat‐clearing and detoxifying formula used for over 1,600 years, has been extensively employed in AD treatment. Previous studies by our research group indicated that HLJDD exhibits significant therapeutic promise in AD models, modulating gut microbiota, attenuating neuroinflammation, and restoring metabolic homeostasis [13, 14]. However, the precise systemic mechanisms through which HLJDD regulates cross‐organ communication remain poorly understood. This study aims to elucidate the cascading mechanisms of glucose metabolism disorder in peripheral and central nervous system inflammation in AD. The multi‐omics analysis was used to integrate different target organs. We further examined whether HLJDD‐associated changes in hepatic inflammatory and metabolic states were accompanied by altered microglial inflammatory responses, including through a hepatocyte–microglia conditioned‐medium model. Ultimately, this work provides a bioinformatic network to quantify the contribution of HLJDD to specific regulatory nodes, offering novel insights and methodologies for AD therapy.

## 2 Result

### 2.1 Multi‐Tissue Transcriptomic Profiling Nominates Systemic Immune Activation and Metabolic Dysregulation in 5xFAD Mice

To investigate AD pathogenesis and identify potential diagnostic and therapeutic targets, we performed transcriptomic profiling on both liver and brain tissues. High‐throughput sequencing yielded high‐quality metrics across all samples, with Q30 scores exceeding 94.3%, stable GC content (48.97%–49.82%), and a base‐calling error rate below 0.03%, confirming the reliability of the data. We identified 462 differentially expressed genes (DEGs) in the 5xFAD brain (Figure 1A). Hierarchical clustering revealed distinct expression patterns between the WT and AD groups (Figure 1B). Functional enrichment analysis showed that brain DEGs were predominantly involved in immune and inflammatory responses, insulin resistance, lipid metabolism, and energy metabolism, with significant enrichment in the PPAR and PI3K/Akt signaling pathways (Figure 1C,D). To evaluate the robustness of these neuroinflammatory findings without relying exclusively on differentially expressed gene thresholds, selected brain transcriptomic signatures were further assessed using complementary GSVA and ssGSEA approaches. GSVA identified higher scores in AD than in WT mice for microglial cell activation, positive regulation of inflammatory response, and astrocyte activation. Consistently, ssGSEA showed increased microglial activation and NF‐κB signaling scores in the AD brain. These findings further supported a robust AD‐associated neuroinflammatory signature, particularly at the level of microglial activation (Figure 1E). Subsequent RT‐PCR validation confirmed that these central immune‐related genes, identified as common DEGs across normal and AD model groups (including the APP/PS1 mice used in the previous efficacy experiments and the 5xFAD mice used in the present experiment), exhibited significant upregulation in AD groups, indicating systemic immune activation in AD brains. Notably, *Itgax* (encoding CD11c, a key marker of microglial activation) was markedly elevated in AD brains (Figure 1F,G). Furthermore, Gene Set Enrichment Analysis (GSEA) indicates functional abnormalities in microglia (Figure 1H,I). Microglia played central roles in the brain, contributing to a multitude of processes ranging from metabolism to immunity. Their dysregulated state was strongly implicated in the pathological processes of neurodegenerative diseases. As resident innate immune cells of the CNS, microglia and astrocytes were critical mediators of brain immune responses. IBA1 and GFAP were established markers of microglial and astrocytic activation, respectively. Immunofluorescence co‐localization staining revealed dense clusters of activated IBA1‐positive microglia and GFAP‐positive astrocytes surrounding Aβ plaques (Figure 1J). Higher‐magnification examination of pathological images revealed dense clusters of activated microglia and astrocytes surrounding Aβ plaques.

**FIGURE 1:**
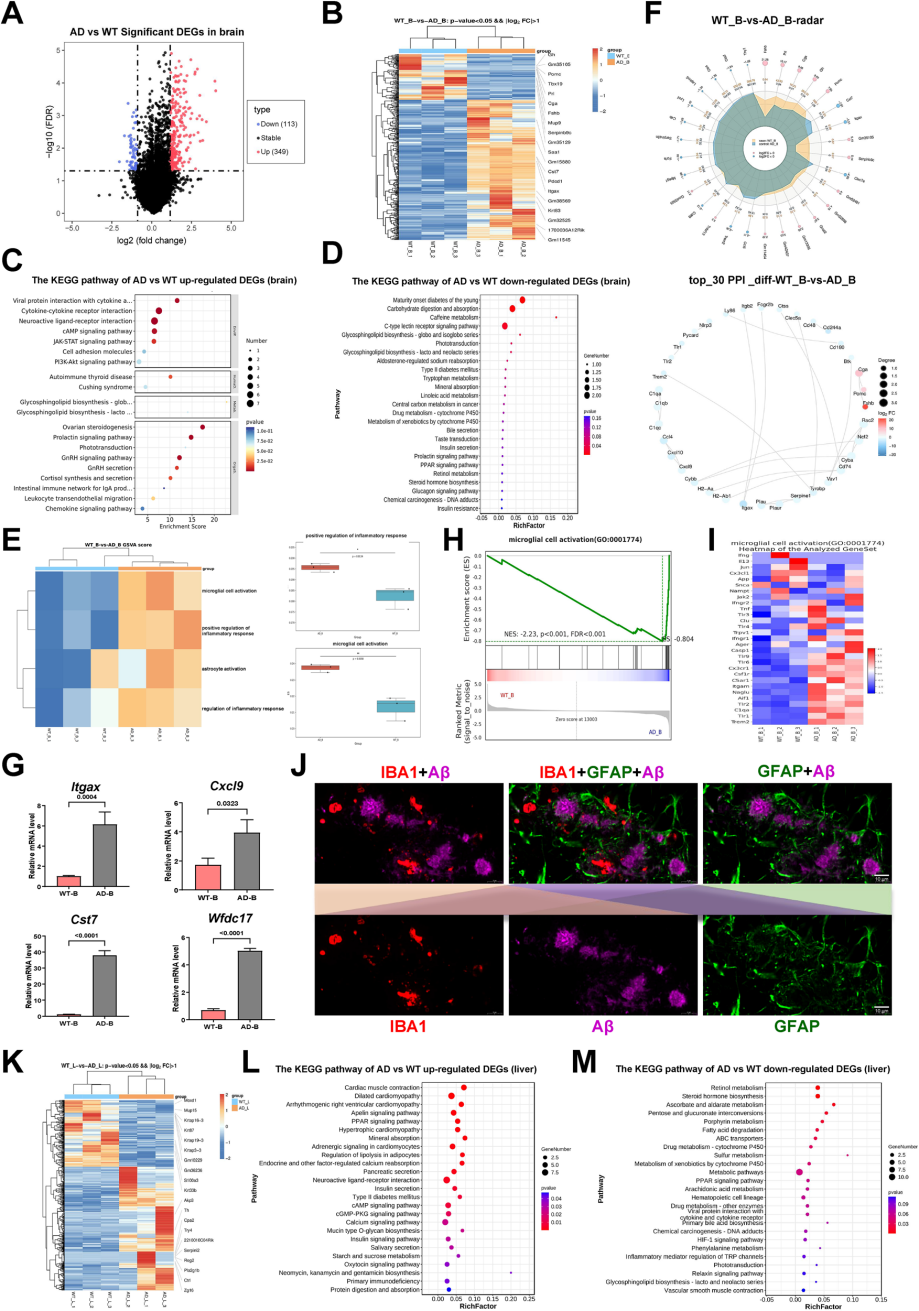
Transcriptomic profiling reveals immune dysregulation and microglial activation signatures in brain and liver tissues of 5xFAD mice. (A) Volcano plot showing differentially expressed genes (DEGs) in brain tissues between AD and WT mice. Red and blue dots represent upregulated and downregulated genes, respectively. (B) Hierarchical clustering heatmap showing the expression patterns of DEGs identified in brain tissues between WT and AD mice. (C, D) KEGG pathway enrichment analysis of DEGs upregulated (C) and downregulated (D) in brain tissues of AD mice compared with WT mice. (E) GSVA‐based pathway activity analysis showing altered immune‐related pathways, including microglial activation and inflammatory response, between WT and AD brain tissues. (F) Radar plot and protein‐protein interaction (PPI) network of the top 30 DEGs in brain tissues of AD mice compared with WT mice. (G) Quantitative PCR validation of *Itgax*, *Cxcl9*, *Cst7*, and *Wfdc17* expression in brain tissues from WT and AD mice (*n* = 3). (H) GSEA enrichment plot of microglial cell activation. (I) Heatmap showing the expression of genes involved in microglial cell activation. (J) Representative immunofluorescence image showing the spatial relationship among Aβ, microglia, and astrocytes in brain tissue from 5xFAD mice (Scale bar = 10 µm). Aβ is displayed as pink; the microglial marker Ionized Calcium‐Binding Adapter Molecule 1 (IBA1) is displayed as red; the astrocyte marker Glial Fibrillary Acidic Protein (GFAP) is displayed as green. (K) Hierarchical clustering heatmap of liver DEGs between WT and AD mice; (L, M) KEGG pathway enrichment analysis of DEGs upregulated (L) and downregulated (M) in liver tissues of AD mice compared with WT mice. For RNA‐seq‐based transcriptomic analyses shown in A‐F, H‐I, and K‐M, *n* = 3 biological replicates per group. Quantitative data are presented as mean ± SEM.

In liver tissues, 573 DEGs (372 downregulated, 201 upregulated) were detected (Figure 1K). Enrichment analysis of liver DEGs revealed prominent alterations in diabetes‐related pathways, energy metabolism, and lipid metabolism. Notably, PPAR‐ and insulin‐related pathways were enriched in both brain and liver tissues, suggesting coordinated central and peripheral molecular alterations (Figure 1L,M).

### 2.2 Integrated Proteomic and Phosphoproteomic Analyses Characterize Signaling and Phosphorylation‐Related Alterations Across Tissues in 5xFAD Mice

Global proteomics identified 8189 proteins in brain tissues (Figure 2A). Phosphoproteomic analysis identified 46711 phosphorylation sites across 6410 proteins, with 4541 overlapping proteins (Figure 2B). PCA showed clear separation between AD and WT groups (Figure 2C). 216 differentially expressed proteins (DEPs) were identified in the AD brain, including 154 upregulated and 62 downregulated proteins (Figure 2D,E). Additionally, 4451 differentially phosphorylated sites (DPPs) were identified, including 2624 upregulated and 1827 downregulated sites (Figure 2F–H).

**FIGURE 2:**
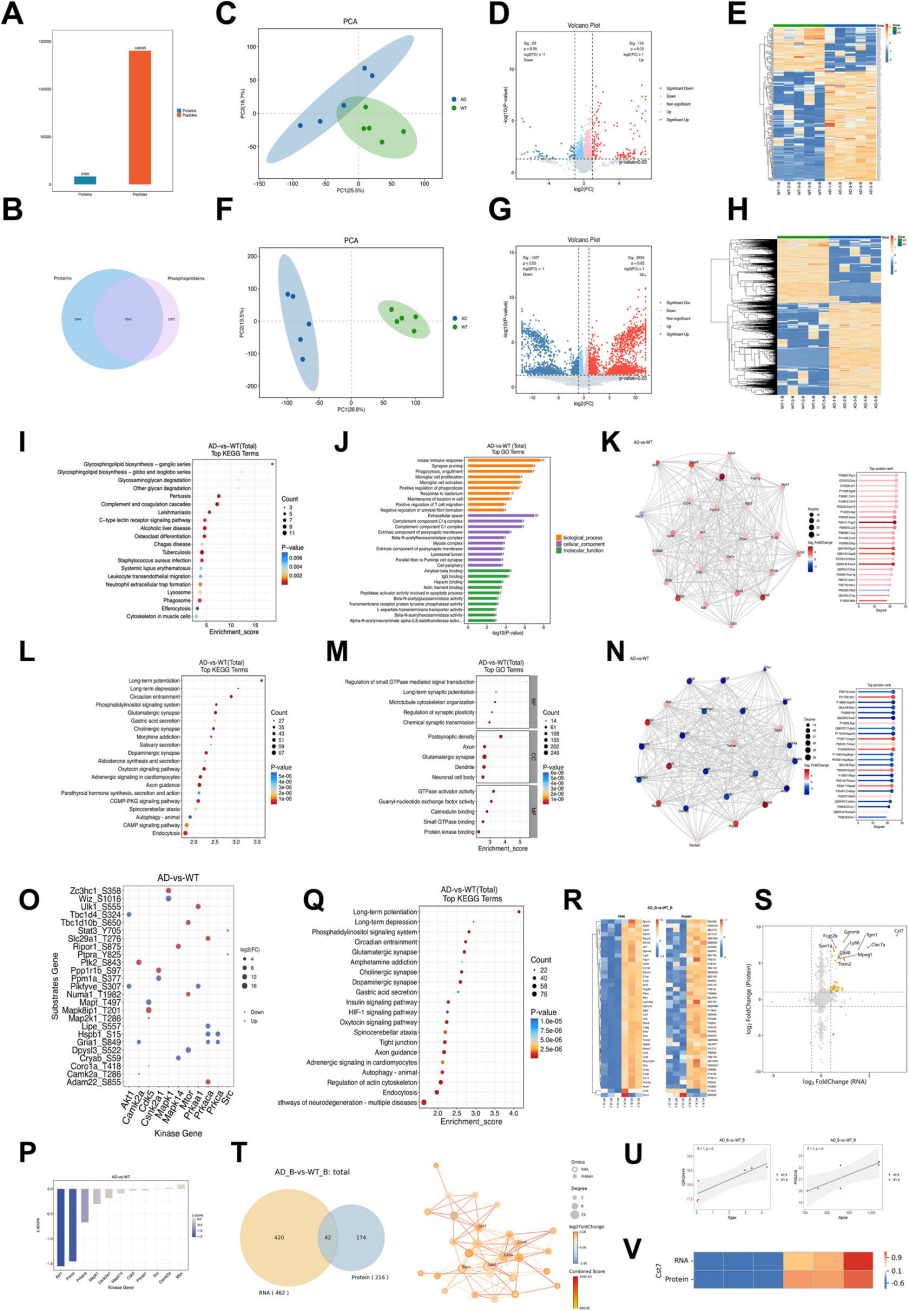
Integrated proteomic and phosphoproteomic analyses of brain tissues from WT and AD mice. (A) Summary of proteins and peptides identified by DIA‐based proteomics in brain tissue. (B) Venn diagram showing the overlap between identified total proteins and phosphoproteins. (C) Principal component analysis (PCA) of DIA proteomic profiles in brain tissues from WT and AD mice. (D, E) Volcano plot (D) and clustering heatmap (E) of differentially expressed proteins (DEPs) in brain tissues between WT and AD mice. (F) PCA of phosphoproteomic profiles in brain tissues from WT and AD mice. (G, H) Volcano plot (G) and clustering heatmap (H) of differentially phosphorylated proteins (DPPs) in brain tissues between WT and AD mice. (I, J) KEGG pathway enrichment (I) and GO term enrichment (J) analyses of DEPs from DIA‐based proteomics in brain tissues between WT and AD mice. (K) PPI network and hub protein ranking of DEPs. (L, M) KEGG pathway enrichment (L) and GO term enrichment (M) analyses of DPPs identified by phosphoproteomics. (N) PPI network and hub protein ranking of DPPs. (O) Bubble plot of the top 20 kinase‐substrate activities. (P) Bar plot of the top 20 enriched kinase activities. (Q) KEGG pathway enrichment analysis based on integrated proteomic and phosphoproteomic data. (R) Multi‐omics clustering heatmap of DEPs and DEGs in brain tissues from WT and AD mice. (S) Quadrant scatter plot comparing fold changes at the RNA and protein levels. (T) Venn diagram of overlapping DEGs and DEPs and their corresponding PPI network. (U) Scatter plots showing RNA‐protein expression correlations for Apoe and Itgax. (V) Heatmap of Cst7 expression at the RNA and protein levels. The displayed R and P values were rounded. R=1 and P=0 therefore represent values close to 1 and 0, respectively. Proteomic and phosphoproteomic analyses in A–Q were performed using *n* = 5 biological replicates per group. Integrated transcriptomic‐proteomic analyses in R‐V were based on transcriptomic data with *n* = 3 biological replicates per group and proteomic data with *n* = 3 biological replicates per group.

In brain tissues, DEPs were enriched in immune and inflammatory pathways including NF‐κB signaling, IL‐17 signaling, complement and coagulation cascades, consistent with transcriptomic evidence of microglial and astrocyte activation. Additionally, metabolic and signaling hubs, such as the PI3K‐Akt and insulin signaling pathways, showed significant protein‐level dysregulation (Figure 2I). GO enrichment analysis further corroborated these findings, showing terms highly consistent with transcriptomic and metabolomic profiles: innate immune response, microglial cell activation, phagocytosis, and positive regulation of the PI3K/Akt signaling cascade (Figure 2J). Collectively, these signatures reflect sustained neuroinflammation, microglial overactivation, and impaired PI3K‐Akt signaling in the AD brain (Figure 2K). KEGG enrichment analysis revealed the phosphatidylinositol signaling system as a core pathway for DPPs (Figure 2L,M). As an upstream component of the PI3K‐Akt pathway, significant alterations in AKT1 phosphorylation at regulatory sites were observed in AD brains (Figure 2N–P). Integrated transcriptomic and proteomic analysis demonstrated significant co‐enrichment of DEGs and DEPs in metabolism‐related pathways, with consistent expression trends of key molecules at both RNA and protein levels, indicating that post‐transcriptional regulation contributes to metabolic disturbances in the brain tissues of AD mice (Figure 2Q–V). Comparative phosphoproteomic analysis revealed significant dysregulation of central insulin signaling, glucose/lipid metabolism, and neuroinflammatory pathways. Specifically, the AD group exhibited inhibitory phosphorylation of the IRS family (Irs1 Ser444; Irs2 Ser965/976/1153) and the AKT‐negative regulator Phlpp1 (Ser313/1482). This was accompanied by downregulated phosphorylation of downstream mTOR (Ser2069) and the energy‐sensor Prkaa1 (Thr488/490), indicating impaired PI3K/AKT signaling and energy homeostasis. Furthermore, the synergistic upregulation of glycogen synthase Gys1 (Ser652/653/657) phosphorylation suggested compromised glucose utilization. AD brain tissues exhibited increased phosphorylation of Nfkb1 (Ser447), Tab3 (Thr408), and Plcg1 (Ser18), together with altered Cyld (Ser394) phosphorylation, indicating coordinated changes in NF‐κB‐related inflammatory signaling. Phosphorylation changes were also observed in PPAR‐ and lipid metabolism‐related proteins, including Sorbs1 (Thr1189/Tyr1193) and Acsl4 (Ser57/Tyr59).

In liver tissues, 6516 proteins and 27154 phosphorylation sites were identified, with 3038 proteins overlapping (Figure S1A,B). PCA (PC1 = 22.2%, PC2 = 18.9%) showed clear separation between AD and WT groups (Figure S1C). A total of 240 DEPs (150 upregulated, 90 downregulated) and 2664 DPPs (1449 upregulated, 1215 downregulated) were identified (Figure S1D–H). Hepatic DEPs were predominantly enriched in PPAR, insulin, and AMPK signaling pathways, as well as fatty acid and lipid metabolism, indicating prominent metabolic alterations in the liver of 5xFAD mice (Figure S1I). GO enrichment analysis revealed marked enrichment in lipid and fatty acid metabolic processes, acyl‐CoA metabolism, peroxisome function, and glutathione and reactive oxygen species metabolism (Figure S1J). The consistency of metabolic regulators at both RNA and protein levels confirmed extensive and coordinated hepatic metabolic reprogramming (Figure S1K–N). Hepatic DPPs were primarily enriched in the PPAR signaling pathway, indicating abnormal phosphorylation‐dependent regulation of lipid metabolism, insulin transduction, and energy homeostasis (Figure S1O–P). Hepatic insulin resistance was characterized by increased inhibitory phosphorylation of Insr (Ser1054), Irs1 (Ser318/1097), and Irs2 (Ser385/675/907/1165), alongside decreased activating phosphorylation of Irs1 (Tyr891) (Figure S1Q). The upregulation of AKT inhibitors (Phlpp1, Ptpn1/11) further inactivated downstream effectors (mTOR, Rps6ka1, Prkaa1). Regarding inflammatory and metabolic remodeling, increased phosphorylation of Map3k7 (Thr417) facilitated the activation of the downstream NF‐κB subunit Rela (Ser238). Significant lipid metabolism alterations were evidenced by the downregulated phosphorylation of Ppargc1b (Ser233), Fabp1 (Ser100), and β‐oxidation enzymes (Acsl1 Ser423; Acsl5 Tyr96), contrasted by the upregulated phosphorylation of lipid droplet proteins (Plin1; Plin5). Furthermore, a synergistic imbalance in glucogenesis and transcriptional regulation was observed through increased phosphorylation of Gys1 (Tyr44), Pck2 (Ser304), and Foxo1 (Thr24), coupled with decreased phosphorylation of Stat3 (Ser727/Thr714) and Creb1 (Ser128). Comparison of overlapping phosphoproteins revealed both shared and tissue‐specific phosphorylation alterations in the brain and liver. Increased phosphorylation of NF‐κB‐related proteins in both tissues was consistent with coordinated inflammatory signaling alterations, whereas distinct phosphorylation sites were observed for several common proteins. For example, IRS1 exhibited reduced Tyr891 phosphorylation in the liver but altered serine phosphorylation in the brain, suggesting tissue‐specific regulation of insulin‐related signaling within a shared molecular framework.

### 2.3 Non‐Targeted Metabolomic Profiling Identifies Coordinated Metabolic Signatures Across Brain, Liver, and Serum in 5xFAD Mice

To dissect metabolic reprogramming mechanisms, we conducted non‐targeted metabolomics using UHPLC‐LTQ‐Orbitrap MS. Samples were fractionated via liquid‐liquid extraction into distinct high‐polarity and low‐polarity fractions, which were subsequently separated using hydrophilic interaction chromatography (HILIC) and reversed‐phase C18 (RP‐C18) columns, respectively. Data acquisition in both positive and negative ionization modes provided comprehensive metabolite coverage. Instrument stability was confirmed by highly consistent retention times and peak intensities of quality control (QC) samples (Figure 3A; Figure S2A). Metabolomic profiling identified a broad range of metabolites across both tissues. Specifically, HILIC and RP chromatography yielded 1745 and 531 metabolites in the brain, and 1671 and 584 in the liver in positive ionization mode, respectively. In negative mode, HILIC and RP detected 1190 and 160 metabolites in the brain, compared to 1170 and 432 in the liver. Detailed information on differential metabolites (DEMs) was provided in Tables S1 and S2. Orthogonal Projections to Latent Structures Discriminant Analysis (OPLS‐DA) evaluated model performance using permutation testing (all permuted R^2^ and Q^2^ values lower than original; Figure 3B,C). In‐depth analysis revealed that DEMs in the brain were primarily enriched in pathways related to neurodegeneration, amino acid, lipid, and purine metabolism (Figure 3D), as well as the AMPK and PI3K‐Akt signaling pathways. In liver tissues, differential metabolites were primarily associated with amino acid, nitrogen, and unsaturated fatty acid metabolism (Figure 3E).

**FIGURE 3:**
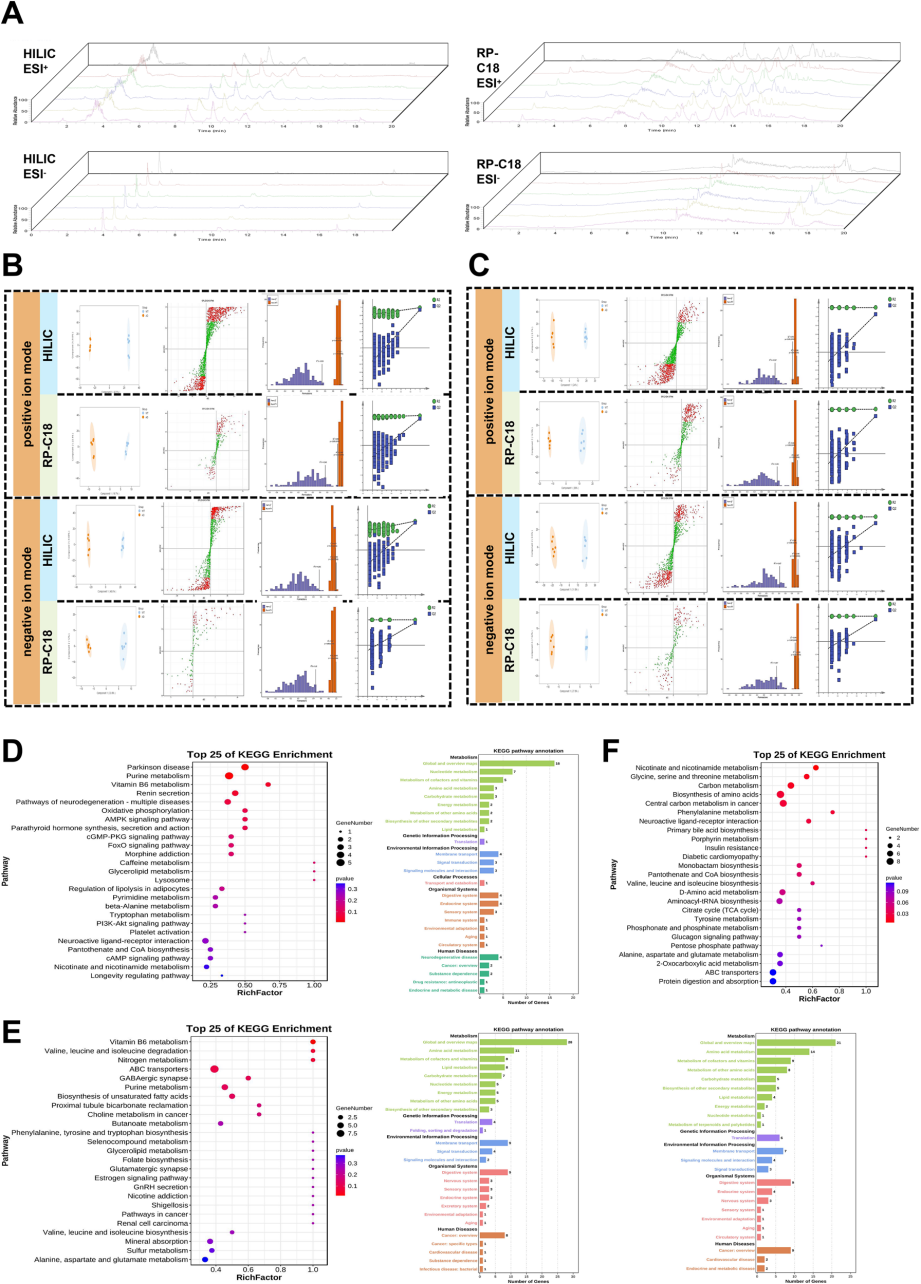
Multi‐tissue metabolomic profiling and pathway enrichment analysis in WT and AD model mice. (A) Overlay of base peak chromatograms (BPCs) from quality control samples of 5xFAD mouse brain tissues under four different detection conditions. (B, C) Orthogonal partial least squares discriminant analysis (OPLS‐DA) score plots of metabolomic profiles in brain tissues (B) and liver tissues (C) from WT and AD mice. (D‐F) KEGG pathway enrichment analysis of differential metabolites in brain tissues (D), liver tissues (E), and serum samples (F) between WT and AD mice. Untargeted metabolomic analyses in B–F were performed using *n* = 6 biological replicates per group.

To further assess circulating metabolic changes associated with the coordinated brain and liver alterations, we performed serum metabolomic profiling. The overlaid BPCs of serum QC samples demonstrated good analytical stability, while OPLS‐DA showed separation among the experimental groups, with permutation testing supporting the validity of the model (Figure S2B,C). Serum DEMs were predominantly enriched in amino acid and energy metabolism pathways (Figure 3F), including pathways related to insulin resistance.

### 2.4 HLJDD Treatment Mitigates Cognitive Deficits, Amyloid‐Beta Burden, and Neuropathological Changes in 5xFAD Mice

LC‐MS analysis enabled the putative characterization of 230 compounds in HLJDD, which mainly include alkaloids, flavonoids, iridoids, and phenolic acids (Table S3). Following a single oral administration of HLJDD, 35 HLJDD‐related compounds were putatively identified in biological samples collected at 2 h, comprising 31 prototype constituents and four metabolites. Of these, 31 compounds were detected in plasma, 30 in liver, and 8 in brain samples (Figure S3; Table S4). The experimental administration schedule was illustrated in Figure 4A. Body weights generally increased  across all groups (Figure 4B; Table S5). WT mice had a significantly higher final body weight than AD mice (*p* < 0.05). In contrast, drug‐treated groups showed weight trajectories similar to the AD group, suggesting that neither HLJDD (high or low dose) nor donepezil significantly affected the growth of 5xFAD mice. Analysis of organ‐to‐body weight ratios showed that the AD group had a significantly higher brain/body ratio (*p* < 0.001) but lower liver (*p* < 0.001) and spleen ratios (*p* < 0.01) than the WT group, potentially indicating systemic metabolic or structural alterations (Figure 4C,D; Table S6). Donepezil treatment significantly increased the liver/body ratio (*p* < 0.05) compared to the AD group, whereas no significant changes were observed in the H‐H and H‐L groups.

**FIGURE 4:**
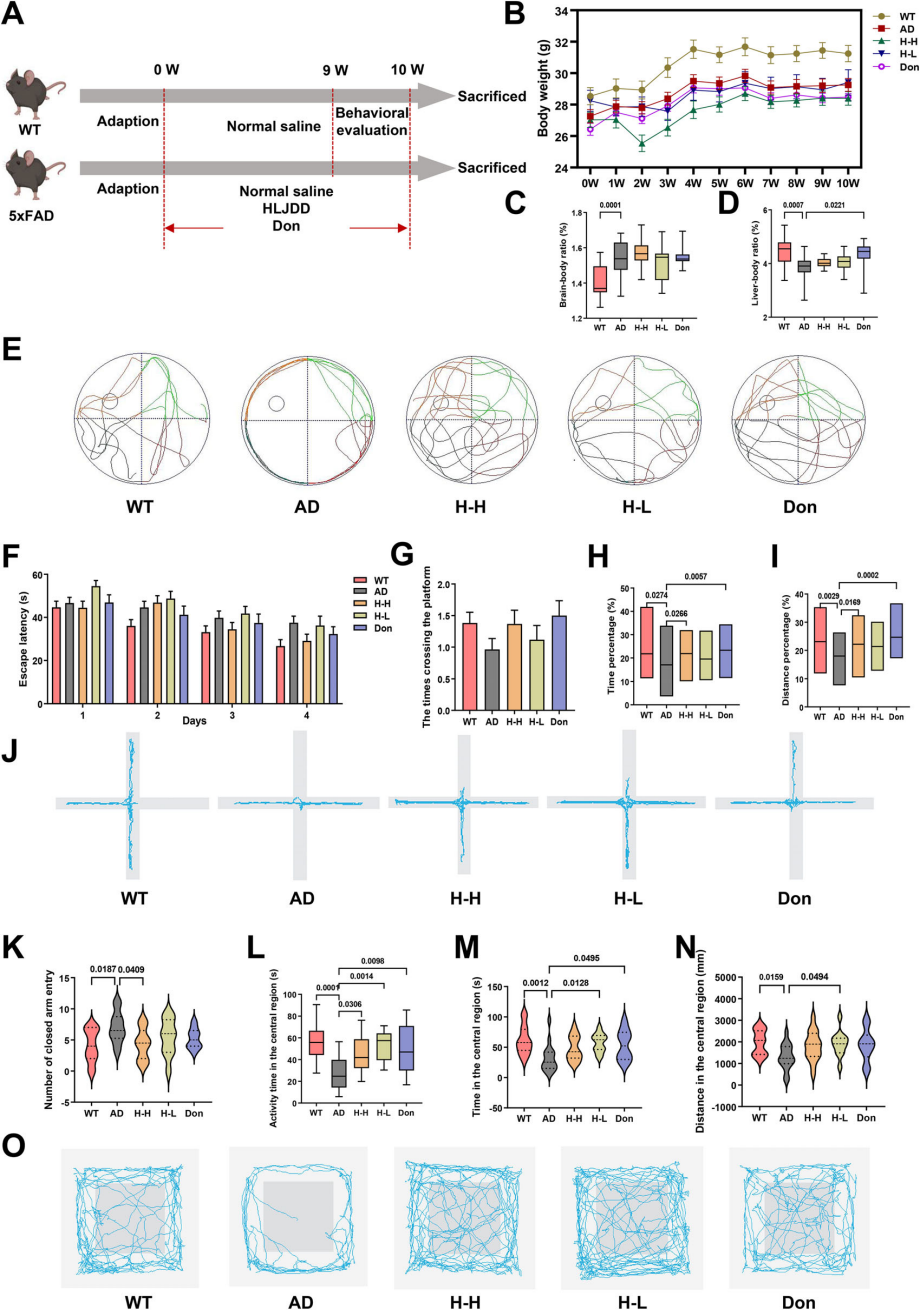
HLJDD improves behavioral deficits in 5xFAD mice. (A) Schedule diagram of the experimental design and treatment schedule. (B) Changes in body weight during the experimental period. (C, D) Brain‐to‐body weight ratio (C) and liver‐to‐body weight ratio (D). (E) Representative swimming trajectories recorded during the 60‐s platform‐free spatial probe trial of the Morris water maze (MWM) on day 5. (F) Escape latency during the four consecutive days of Morris water maze acquisition training (days 1–4). (G) Number of platform crossings in the MWM probe test. (H, I) Percentage of time spent in the target quadrant (H) and percentage of distance traveled in the target quadrant (I) during the MWM probe test. (J) Representative movement trajectories in the elevated plus maze. (K) Number of closed‐arm entries in the elevated plus maze. (L) Active time in the central area in the open field test. (M) Time spent in the central area in the open field test. (N) Distance traveled in the indicated region of the open field test. (O) Representative movement trajectories in the open field test. Body weight, organ index, and behavioral analyses in B–O were performed using WT, AD, H‐H, H‐L, and Don groups, with *n* = 15, 16, 16, 11, and 10 mice, respectively. Quantitative data are presented as mean ± SEM.

The MWM experiment was used to evaluate spatial learning and memory abilities. Representative swimming trajectories are shown in Figure 4E. During the acquisition phase, escape latency gradually decreased over the four training days in all groups. The AD group tended to exhibit longer escape latencies during the later training days, whereas the Don and H‐H groups showed shorter escape latencies than the AD group (Figure 4F). No significant difference in escape latency was observed between the H‐H and H‐L groups. In the spatial probe trial, compared with the WT group, the AD group spent a lower percentage of time in the target quadrant (*p* < 0.05) and traveled a lower percentage of the total distance in the target quadrant (*p* < 0.01). Compared with the AD group, the Don and H‐H groups showed a higher percentage of time spent in the target quadrant (*p* < 0.01, *p* < 0.05, respectively) and distance traveled in the target quadrant (*p* < 0.001, *p* < 0.05, respectively), and an increased number of platform crossings. No significant differences in the probe‐trial outcomes were observed between the H‐L and AD groups (Figure 4G–I).

In the EPM experiment, locomotor and anxiety‐related outcomes were analyzed separately. Total distance traveled, speed, and total arm entries did not differ significantly among groups, suggesting that the observed behavioral changes were not primarily attributable to differences in basal locomotor activity. Anxiety‐like behavioral parameters were therefore analyzed separately. Compared with the WT group, the AD group exhibited a greater number of closed‐arm entries (*p* < 0.05). The number of closed‐arm entries was lower in the H‐H group than in the AD group (*p* < 0.05), whereas neither the H‐L nor the Don group differed significantly from the AD group (Figure 4J,K).

In the OFT experiment, locomotor activity‐ and anxiety‐like behavior‐related parameters were also analyzed separately. The AD group exhibited decreased movement linearity (*p* < 0.05), reduced total distance traveled (*p* < 0.05), less time spent in the central area (*p* = 0.0012), and an increased number of fecal boli (*p* < 0.05), compared to the WT group. Compared with the AD group, the H‐H group showed increased active time and time in the central area (*p* < 0.05). The H‐L group exhibited increased active time in the central region (*p* < 0.01) and a greater distance traveled in the central area (*p* < 0.05). The Don group also showed increased active time (*p* < 0.01) and time spent in the central region (*p* < 0.05) (Figure 4L–O).

The HE staining results showed that in the brain of WT group mice, the neurons in the hippocampal region were arranged tightly and orderly, with no signs of degeneration, necrosis, or inflammatory cell infiltration. The morphological structures of the cell membranes and nuclei were normal. In contrast, in the hippocampal region of AD group mice, some neurons exhibited degeneration and necrosis, along with mild microglial proliferation and the deposition of numerous amyloid plaques. Similar observations were made in the cerebral cortex, where a large amount of amyloid plaque deposition and microglial proliferation were observed. In the Don group, although there were a small number of degenerated and necrotic neurons and amyloid plaque depositions in the hippocampal region, the overall pathological changes were less severe than in the AD group. The cortical neurons also maintained good structural integrity. Compared to the AD group, the number of amyloid plaque depositions in the H‐H group was reduced, and no significant pathological changes were observed. However, the pathological changes in the H‐L group were similar to those in AD group, with partial neurons exhibiting degeneration and necrosis, numerous amyloid plaque depositions, and mild microglial proliferation (Figure 5A). The positive staining intensities of Congo red (Figure 5B,C), Aβ (Figure 5D–F), and PS1 (Figure 5G–I) in the cortical and hippocampal regions of the AD group mice brain tissue were significantly higher than those of WT group mice (*p* < 0.05). The positive staining areas of Congo red, Aβ, and PS1 in the cerebral cortex of the H‐H group mice were significantly reduced (*p* < 0.05). In the H‐L group, the positive staining areas of Aβ and PS1 were significantly reduced (*p* < 0.05), but the reduction was not as significant as in the H‐H group.

**FIGURE 5:**
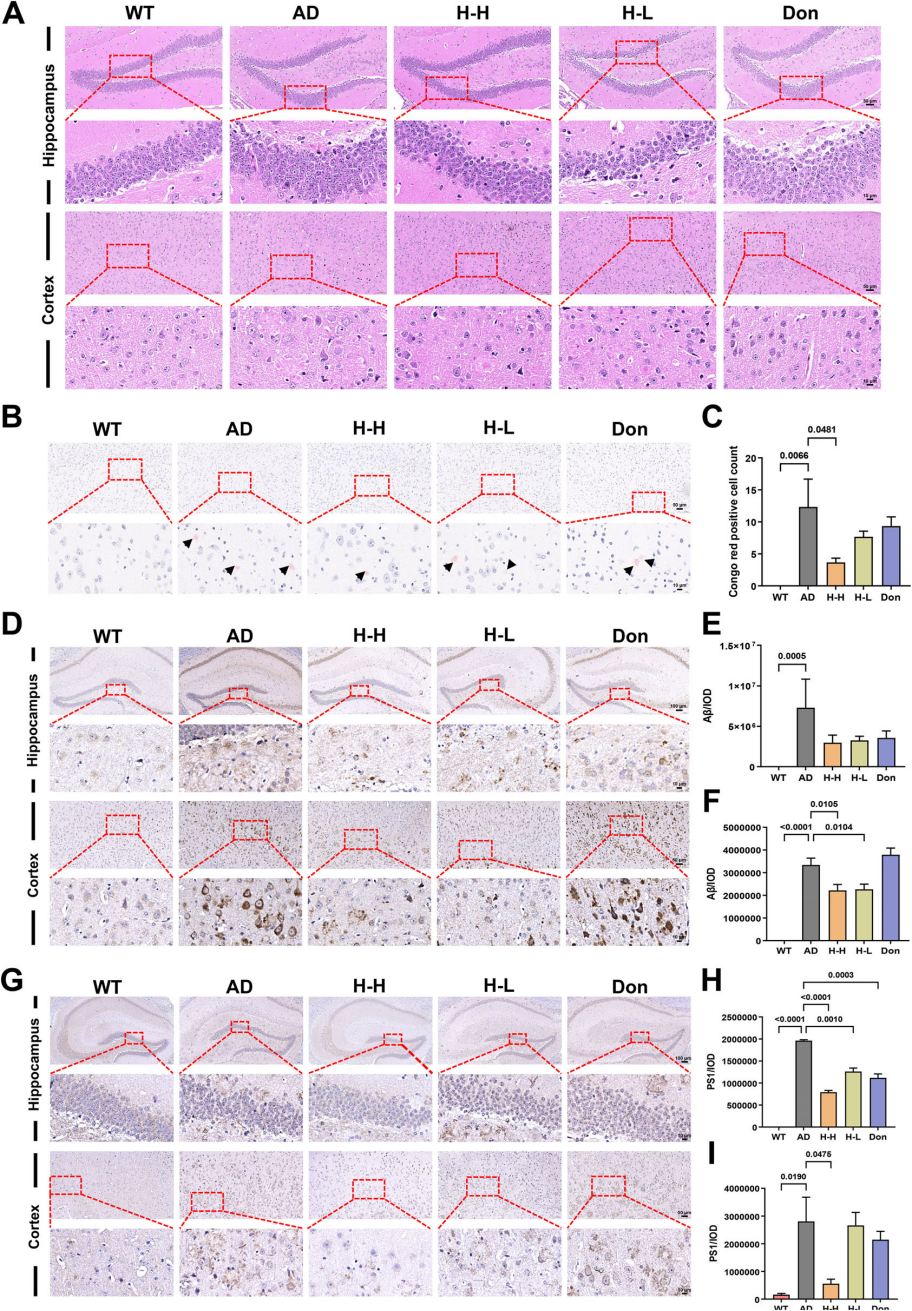
HLJDD ameliorates neuropathological alterations in brain tissues of 5xFAD mice. (A) Representative H&E‐stained images of brain sections (Scale bar = 50 µm and 10 µm). (B) Representative Congo red‐stained images of brain sections (Scale bar = 50 µm and 10 µm). (C) Quantification of Congo red‐positive amyloid deposition. (*n* = 3 mice/group; each *n* represents the average of three technical replicates). (D) Representative immunohistochemical staining images of Aβ in the hippocampal and cortical regions (Scale bars: 100 µm for hippocampal overview images, 50 µm for cortical overview images, and 10 µm for enlarged images). (E, F) Quantification of Aβ immunohistochemical staining in the hippocampus (E) and cortex (F) (*n* = 3 mice/group; each *n* represents the average of three technical replicates). (G) Representative immunohistochemical staining images of PS1 in the hippocampal and cortical regions (Scale bars: 100 µm for hippocampal overview images, 50 µm for cortical overview images, and 10 µm for enlarged images). (H, I) Quantification of immunohistochemical staining images of PS1 in the hippocampus (H) and cortex (I) (*n* = 3 mice/group; each *n* represents the average of three technical replicates).

### 2.5 HLJDD Treatment Partially Restores Transcriptomic Signatures Related to PPAR Signaling and Immune Metabolic Regulation in 5xFAD Mice

Differential expression analysis identified 437 DEGs (213 downregulated, 224 upregulated) in HLJDD‐vs‐AD brain, with 16 AD‐upregulated genes being significantly downregulated by HLJDD treatment and 12 AD‐downregulated genes rescued to upregulated status (Figure 6A–D). In liver tissues, HLJDD intervention modulated 263 DEGs (Figure 6E). HLJDD‐treated groups showed DEG enrichment in inflammatory immunity, amino acid metabolism, energy metabolism, and PPAR signaling pathways (Figure 6F–H). In HLJDD‐treated liver tissues, DEGs were primarily enriched in amino acid and energy metabolism (Figure 6I–K). Transcriptomic profiling further demonstrated robust immune activation in AD brains and consistent dysregulation of the PPAR pathway and diabetes/insulin‐related pathways across brain and liver tissues, suggesting that HLJDD may exert therapeutic effects by activating the PPAR pathway to modulate metabolic homeostasis and attenuate inflammatory responses in AD.

**FIGURE 6:**
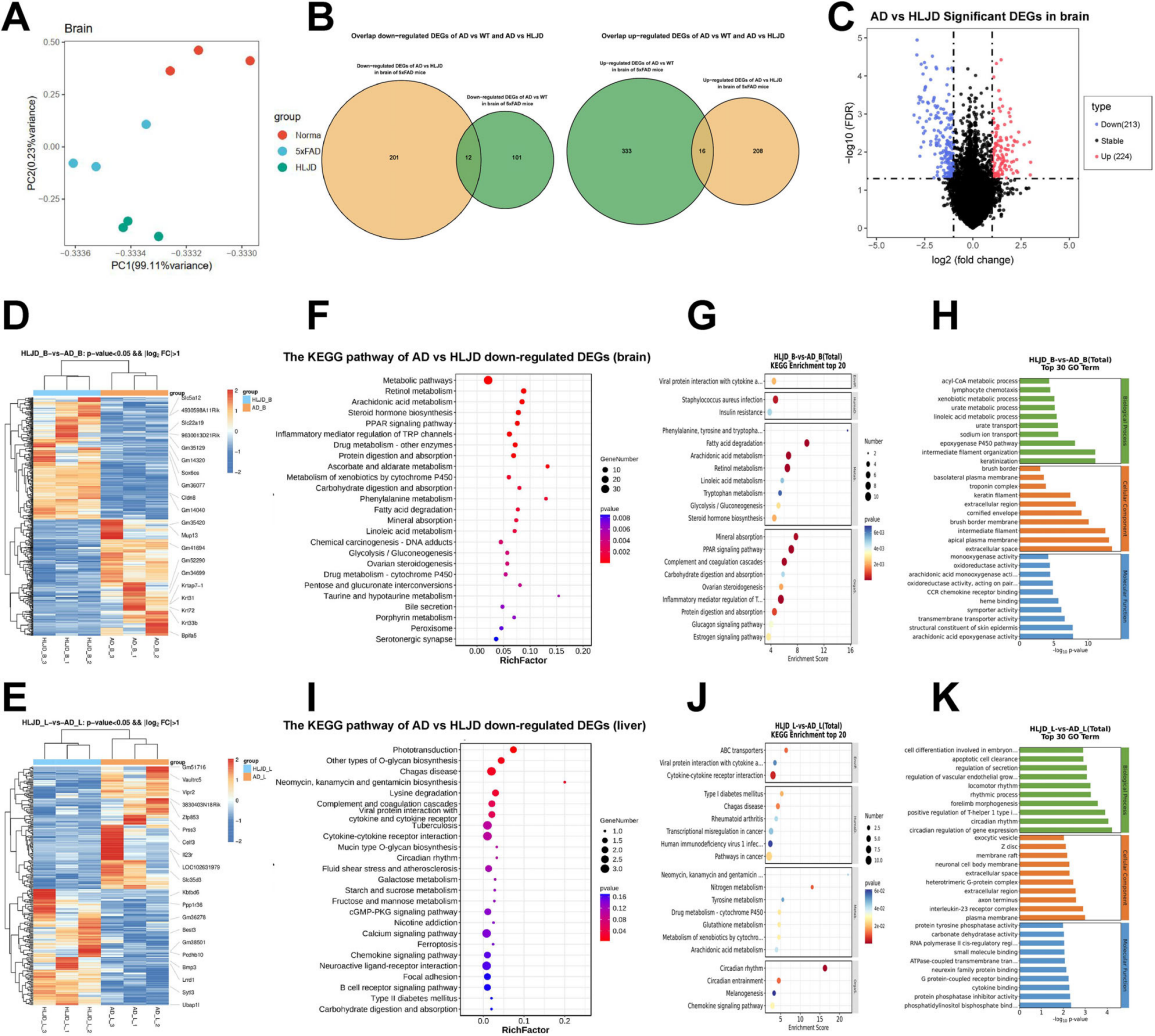
HLJDD reverses transcriptional alterations in brain and liver tissues of 5xFAD mice. (A) PCA of brain transcriptomic profiles from WT, AD, and HLJDD‐treated mice. (B) Venn diagram showing the overlap of DEGs in brain tissues. (C) Volcano plot of DEGs in brain tissues between AD and HLJDD‐treated mice. (D) Clustering heatmap of brain DEGs between AD and HLJDD‐treated mice. (E) Clustering heatmap of liver DEGs between AD and HLJDD‐treated mice. (F) KEGG pathway enrichment analysis of downregulated DEGs in brain tissues from HLJDD‐treated mice compared with AD mice. (G) KEGG enrichment bubble plot of total DEGs in brain tissues between HLJDD‐treated and AD mice. (H) GO enrichment analysis of brain DEGs between HLJDD‐treated and AD mice. (I) KEGG pathway enrichment analysis of downregulated DEGs in liver tissues from HLJDD‐treated mice compared with AD mice. (J) KEGG enrichment bubble plot of total DEGs in liver tissues between HLJDD‐treated and AD mice. (K) GO enrichment analysis of liver DEGs between HLJDD‐treated and AD mice. Transcriptomic analyses in A‐K were performed using *n* = 3 biological replicates per group.

### 2.6 HLJDD Treatment Is Associated with Phosphoproteomic Remodeling of Insulin and Inflammatory Signaling in Brain and Liver Tissues

After HLJDD treatment, 102 DEPs were identified in the brain tissues of AD mice, including 44 upregulated and 58 downregulated proteins (Figure 7A–C). A total of 3446 DPPs were identified, including 2040 up‑regulated and 1406 down‑regulated phosphorylation sites (Figure 7D–F). KEGG pathway enrichment of brain DEPs further showed consistency with the transcriptomic profiles. Immune‐ and inflammation‐related pathways, including NF‐κB signaling and efferocytosis, were enriched, consistent with the transcriptomic evidence of immune activation in AD brains and supporting HLJDD‐associated modulation of inflammatory responses (Figure 7G). Metabolic pathways such as retinol metabolism and phenylalanine metabolism were enriched. The insulin secretion pathway was also enriched, consistent with the transcriptomic identification of diabetes/insulin‐related pathway dysregulation. DEPs in brain tissues were significantly enriched in GO biological processes that aligned with transcriptomic findings. Key enriched processes included positive regulation of acute inflammatory response, which corresponded to the transcriptomic focus on inflammatory immunity and supported the attenuation of neuroinflammation by HLJDD (Figure 7H). Processes related to synaptic function, such as chemical synaptic transmission and dopaminergic synaptic transmission, were also enriched. Additionally, dopamine metabolic processes were enriched. In brain tissues, HLJDD treatment was associated with changes in insulin‐related phosphorylation patterns, including reduced phosphorylation of selected inhibitory sites on insulin receptor substrates (Figure 7I,J). Specifically, the phosphorylation levels of Irs1 (Ser444) and Irs2 (Ser976 and Ser1153) were downregulated following HLJDD intervention. Concurrently, HLJDD treatment was associated with increased phosphorylation of Tbc1d4 at Ser324 and Ser764 and Gsk3b at Tyr323, together with reduced phosphorylation of Phlpp1 at Ser293. Regarding energy metabolism pathways, HLJDD treatment was associated with increased phosphorylation of Prkaa1 (AMPKα1) at Thr382 and of Eif4b at Ser409 and Ser422, indicating treatment‐associated changes in energy‐ and translation‐related signaling. KEGG enrichment analysis of these brain DEPs and phosphosites showed substantial overlap with transcriptomic findings, particularly in immune‐ and metabolism‐related pathways (Figure 7K–N). Notably, whereas transcriptomic analysis identified diabetes‐related pathway alterations, phosphoproteomic analysis showed enrichment of pathways related to Glycolysis/Gluconeogenesis, the Citrate cycle (TCA cycle), and the Pentose phosphate pathway, consistent with phosphorylation‐level remodeling of energy metabolism‐related processes. Furthermore, enrichment of Insulin secretion and Insulin resistance pathways at the phosphorylation level suggested treatment‐associated remodeling of insulin‐related signaling. These changes occurred alongside enrichment of synaptic and dopaminergic transmission‐related processes.

**FIGURE 7:**
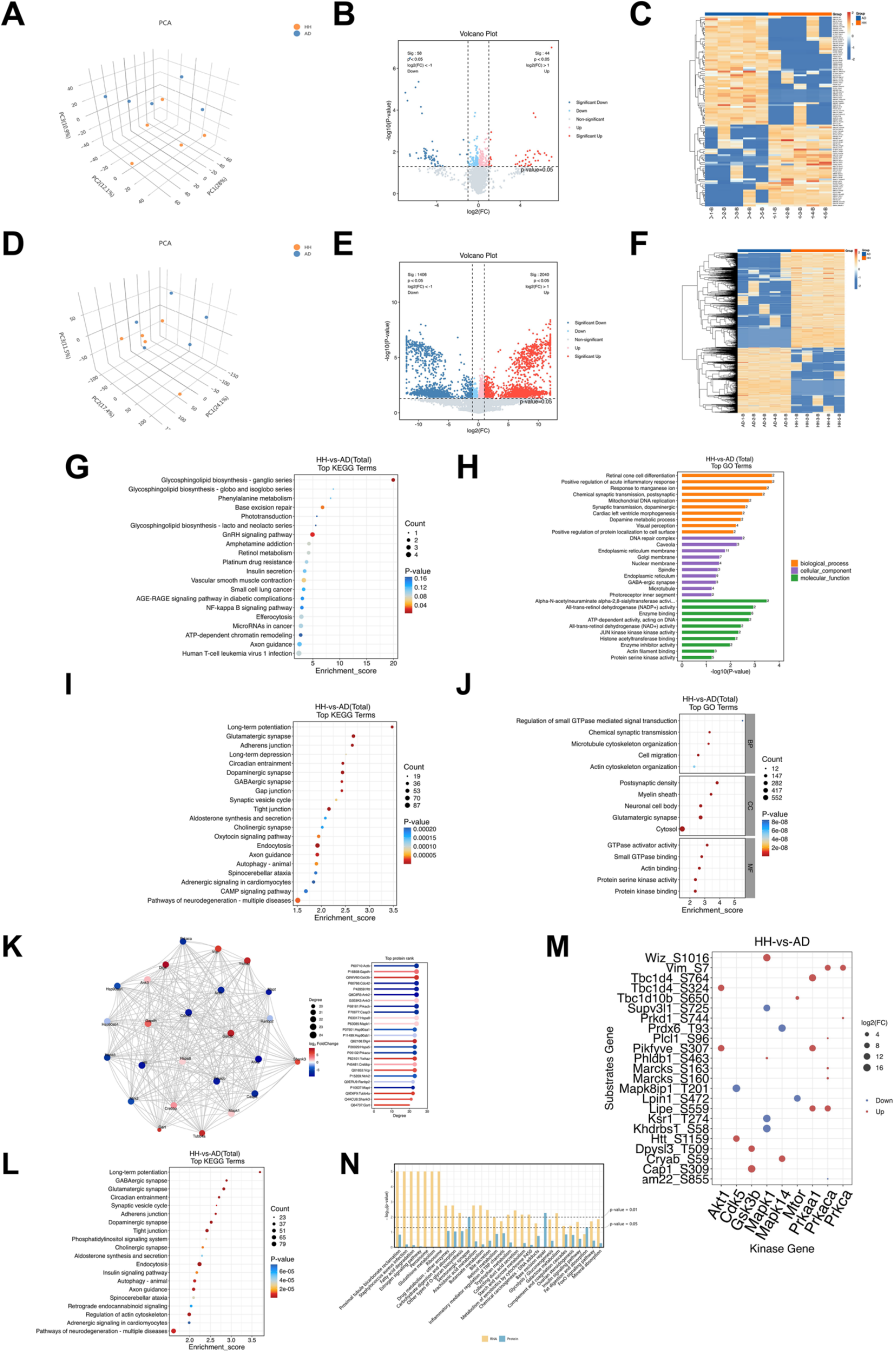
Integrated proteomic and phosphoproteomic profiling of brain tissues after HLJDD treatment in 5xFAD mice. (A) PCA of DIA proteomic profiles in brain tissues from AD and HLJDD‐treated mice. (B, C) Volcano plot (B) and clustering heatmap (C) of DEPs in brain tissues between HLJDD‐treated and AD mice. (D) PCA of phosphoproteomic profiles in brain tissues from AD and HLJDD‐treated mice. (E, F) Volcano plot (E) and clustering heatmap (F) of DPPs in brain tissues between HLJDD‐treated and AD mice. (G, H) KEGG pathway enrichment (G) and GO term enrichment (H) analyses of DEPs identified by DIA‐based proteomics. (I, J) KEGG pathway enrichment (I) and GO term enrichment (J) analyses of DPPs identified by phosphoproteomics. (K) PPI network and hub protein ranking of DPPs. (L) KEGG pathway enrichment analysis based on integrated proteomic and phosphoproteomic data in brain tissues between HLJDD and AD groups. (M) Kinase activity enrichment analysis in brain tissues from AD and HLJDD‐treated mice. (N) GSEA bar plot based on integrated transcriptomic and proteomic analysis in brain tissues from AD and HLJDD‐treated mice. Proteomic and phosphoproteomic analyses in A‐M were performed using *n* = 5 biological replicates per group. Integrated transcriptomic‐proteomic analysis in N was based on RNA‐seq data with *n* = 3 biological replicates per group and proteomic data with *n* = 3 biological replicates per group.

In liver tissues, 159 DEPs were detected, with 54 upregulated and 105 downregulated proteins. A total of 2443 DPPs were identified, including 1048 up‑regulated and 1395 down‑regulated phosphorylation sites (Figure S4A–F). DEPs and DPPs were predominantly enriched in the PPAR signaling pathway, AMPK signaling pathway, insulin secretion pathway, and type II diabetes mellitus‐related pathways (Figure S4G–L). These enrichments were concordant with transcriptomic evidence of metabolic reprogramming and metabolomic profiles of lipid and amino acid metabolism (Figure S4M,N). Moreover, pathways including arachidonic acid metabolism, glutathione metabolism, and non‐alcoholic fatty liver disease‐related pathways were enriched, which further indicated HLJDD‐associated changes in hepatic metabolism‐related pathways. In liver tissues, the HLJDD group exhibited significant anti‐inflammatory and metabolic remodeling effects. HLJDD treatment was associated with changes in the phosphorylation of hepatic Insr (Ser1054) and Irs1/2 at sites altered in the AD group. Simultaneously, it upregulated the phosphorylation of the Akt2 (Thr451) activation site and restored the phosphorylation modifications of downstream Gys1 and Foxo1, suggesting changes in hepatic insulin‐related signaling and glucose metabolism. In inflammatory signaling‐related proteins, HLJDD treatment reduced phosphorylation of Map3k7 at Thr417 and Ser389 and of Rela (p65) at Ser238, consistent with attenuation of NF‐κB‐related inflammatory signaling. Regarding lipid metabolism, HLJDD treatment was associated with reduced phosphorylation of Plin5 and Plin1 and increased phosphorylation of Fabp1 and Ppargc1b (PGC‐1β), indicating treatment‐associated remodeling of lipid metabolism‐related signaling.

Notably, the insulin secretion pathway was enriched in both brain and liver tissues, suggesting shared alterations in insulin‐related signaling across the two organs. Integrated analysis further showed that HLJDD treatment was associated with reduced inhibitory serine phosphorylation of IRS1/2 at selected sites and with attenuation of inflammatory signaling in both tissues. In parallel, reduced Rela phosphorylation and other inflammatory changes were observed following HLJDD treatment, consistent with coordinated modulation of metabolic and inflammatory processes in the brain and liver.

### 2.7 HLJDD Treatment Is Associated with Remodeling of Central and Peripheral Metabolic Signatures in 5xFAD Mice

Principal component analysis (PCA) demonstrated clear separation between experimental groups for both tissues (Figures S5A,B and S6A,B). After HLJDD administration, the differential metabolites in the mouse brain tissues mainly concentrated in several key metabolic pathways, including oxidative phosphorylation, amino acid metabolism, lipid metabolism, and purine metabolism (Figure S5C). At the same time, two important signaling pathways, AMPK signaling and PI3K‐Akt signaling pathways, were also significantly affected. To further understand the function and role of the PI3K‐Akt signaling pathway, we conducted related validation work in the subsequent verification study. The liver OPLS‐DA model was evaluated using cross‐validation and permutation testing (Figure S5D). In liver tissues, differential metabolites between the AD and HLJDD groups were mainly enriched in amino acid metabolism, purine metabolism, and nitrogen metabolism (Figure S5E). In the comparison of serum between AD and HLJDD mice, the DEMs were enriched in glycolysis, insulin resistance, amino acid metabolism, energy metabolism, and lipid metabolism pathways (Figure S5F,G).

### 2.8 HLJDD Attenuates Systemic Inflammation by Suppressing Glial Activation and Pro‐inflammatory Cytokine Release

Multi‐omic transcriptomic and proteomic analysis revealed that DEGs/DEPs in mouse brain tissue were predominantly enriched in immune‐ and inflammation‐related signaling pathways (Figure 8A). Complementary ssGSEA analysis showed altered astrocyte activation and microglial cell activation scores in the brains of HLJDD‐treated mice compared with AD mice (Figure 8B). GSEA and gene expression heatmap analyses (Figure 8C,D) suggested that HLJDD modulates the astrocyte differentiation pathway and the expression of its related core genes, potentially contributing to the amelioration of systematic metabolic disorders in AD mice. Subsequently, DEGs identified in the brain tissue between the HLJDD and AD groups were selected and validated using RT‐PCR. The RT‐PCR results demonstrated that HLJDD treatment significantly reversed the expression levels of *Ly86*, *Cd68*, and *Itgax* (Figure 8E). These findings indicated that genes associated with central immunity were markedly upregulated in the AD group, whereas HLJDD treatment effectively downregulated.

**FIGURE 8:**
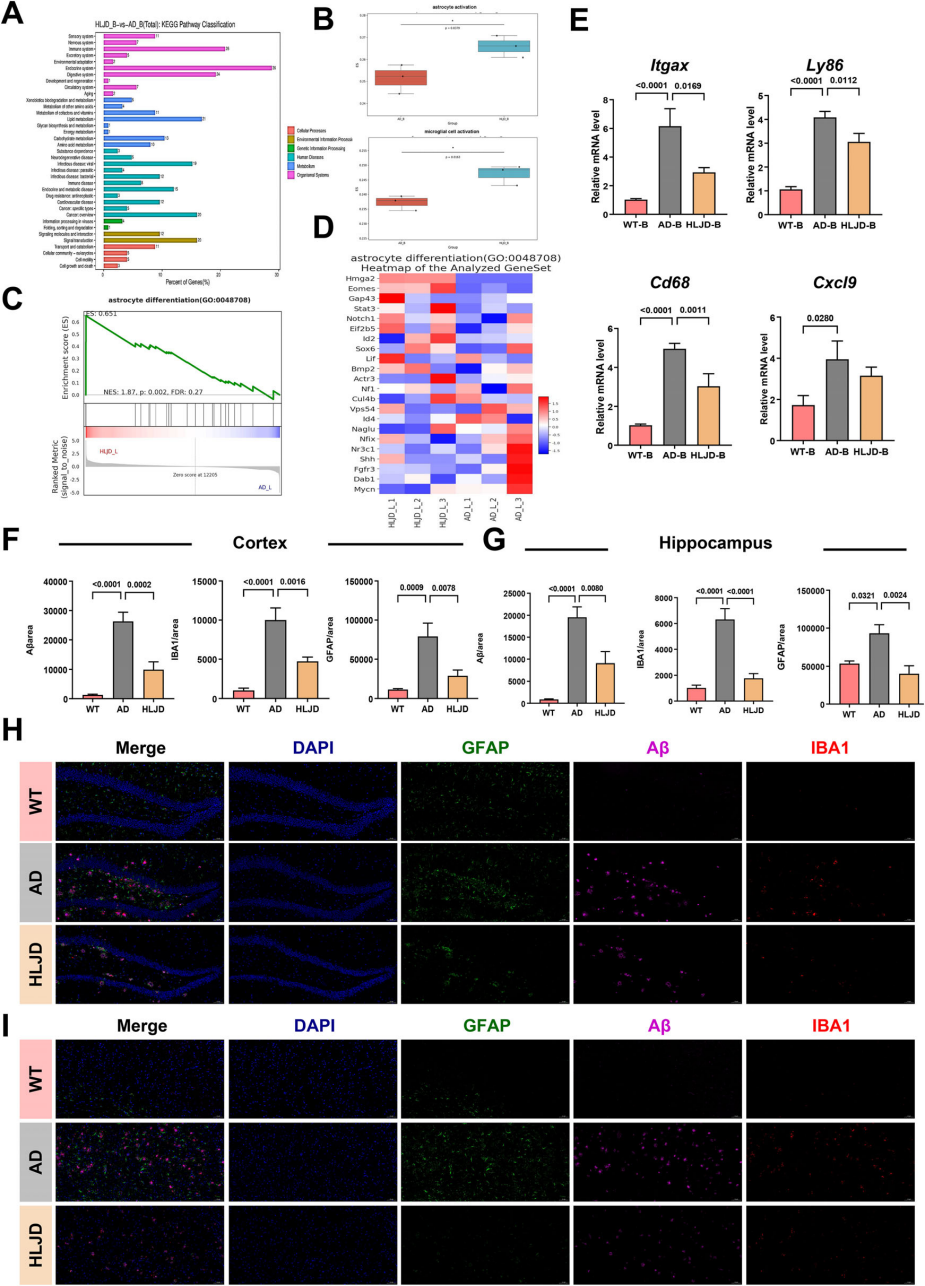
Effect of HLJDD on central immunity in 5xFAD mice. (A) KEGG secondary classification plot of total DEGs in brain tissues between HLJDD‐treated and AD mice. (B) Single‐sample gene set enrichment analysis (ssGSEA) scores for astrocyte activation (upper panel) and microglial cell activation (lower panel) in AD and HLJDD‐treated mice. (C) GSEA enrichment plot of astrocyte differentiation. (D) Heatmap showing the expression of genes involved in astrocyte differentiation. (E) qRT‐PCR analysis of immune‐related genes in brain tissues from 5xFAD mice treated with HLJDD (*n* = 3). (F, G) Quantification of Aβ, IBA1, and GFAP immunofluorescence in the cortex (F) and hippocampus (G) (*n* = 3 mice/group; each *n* represents the average of three technical replicates). (H, I) Representative immunofluorescence images of Aβ, IBA1, and GFAP in the hippocampus (Scale bar = 50 µm) (H) and cortex (Scale bar = 50 µm) (I); Aβ is displayed as pink, the microglial marker Ionized Calcium‐Binding Adapter Molecule 1 (IBA1) is displayed as red, the astrocyte marker Glial Fibrillary Acidic Protein (GFAP) is displayed as green, and the nuclear marker 4',6‐diamidino‐2‐phenylindole (DAPI) is displayed as blue. Quantitative data are presented as mean ± SEM.

Immunofluorescence results clearly showed significantly elevated levels of Aβ, IBA1 (microglia), and GFAP (astrocytes) in the hippocampal and cortical regions of 5xFAD mice (Figure 8F,G). Treatment with HLJDD markedly reduced Aβ accumulation and attenuated the aberrant activation of both microglia and astrocytes (Figure 8H,I). These findings suggested that HLJDD might improve cognitive function and mitigate neuroinflammation, potentially by modulating the interactions between innate immune cells and Aβ plaques.

### 2.9 Restoration of Central and Peripheral Energy Metabolism and Redox Balance by HLJDD

When microglia shifted toward an inflammatory phenotype, they released pro‐inflammatory cytokines such as IL‐1β, TNF‐α, and IL‐6, which exerted neurotoxic effects within the nervous system. GSEA analysis of brain tissue transcriptomics revealed significantly elevated TNFα production in the AD group (Figure S7A), with heatmap analysis confirming enhanced expression of related genes (Figure S7B). RT‐PCR validation demonstrated markedly increased mRNA levels of pro‐inflammatory factors including TNFα, IFN‐γ, IL‐1β, and IL‐6 in AD model brain tissue (Figure S7C–E). Notably, HLJDD treatment substantially reduced mRNA expression of these cytokines. The mRNA levels of IL‐6 in both the brain and liver tissues of AD mice were significantly increased (Figure S7D,E), indicating that the central and peripheral systems were consistent in the inflammatory response of AD. Microglial activation and adoption of an inflammatory phenotype would trigger the release of reactive oxygen species (ROS). Biochemical analyses revealed decreased nitric oxide (NO) levels accompanied by reduced glutathione (GSH), increased superoxide dismutase (SOD), and elevated 4‐hydroxynonenal (4‐HNE) content in both central and peripheral systems of AD mice (Figure S7F). These findings collectively indicated an oxidative stress state characterized by diminished antioxidant capacity and accumulation of lipid peroxidation products. However, HLJDD intervention significantly restored NO levels (Figure S7F), particularly in liver tissue with statistical significance, while concurrently reducing oxidative stress biomarkers in brain tissue.

### 2.10 Impact of HLJDD on Central and Peripheral Energy Metabolism in 5xFAD Mice

Microglia shifted toward an energetic phenotype, characterized by enhanced glycolysis and dysfunctional mitochondrial respiration. This transformation revealed the close connection between neuroinflammation and metabolic dysregulation. Transcriptomic and metabolomic analyses of brain and liver tissues jointly revealed disturbances in energy metabolism pathways (Figure S8A–D). Biochemical assays further confirmed this point (Figure 9D). The level of glucose was significantly reduced in the brain and liver of the AD group, while lactate was elevated compared to the WT group. The treatment of HLJDD normalized these perturbed glucose and lactate levels, with parallel recovery trends observed in both central and peripheral systems. Based on the top 30 protein‐protein interaction (PPI) networks of DEGs between HLJDD and AD groups (Figure 9B), the key targets were validated via PCR. Results demonstrated significantly elevated expression of *Adipoq*—a gene critically linked to insulin resistance—in both brain and liver tissues of AD mice. HLJDD intervention effectively suppressed its overexpression. Furthermore, HLJDD upregulated expression of glucose metabolism‐related genes (*Pck1*, *Fbp1*, *Atp2a1*) in brain tissue while normalizing the dysregulation of *Fabp4*, *Lep*, and *Elk1*. In liver tissue, HLJDD significantly modulated the expression of *Ugt1a9*, *Hes1*, and *Obp2a* (Figure 9F; Figure S8E).

**FIGURE 9:**
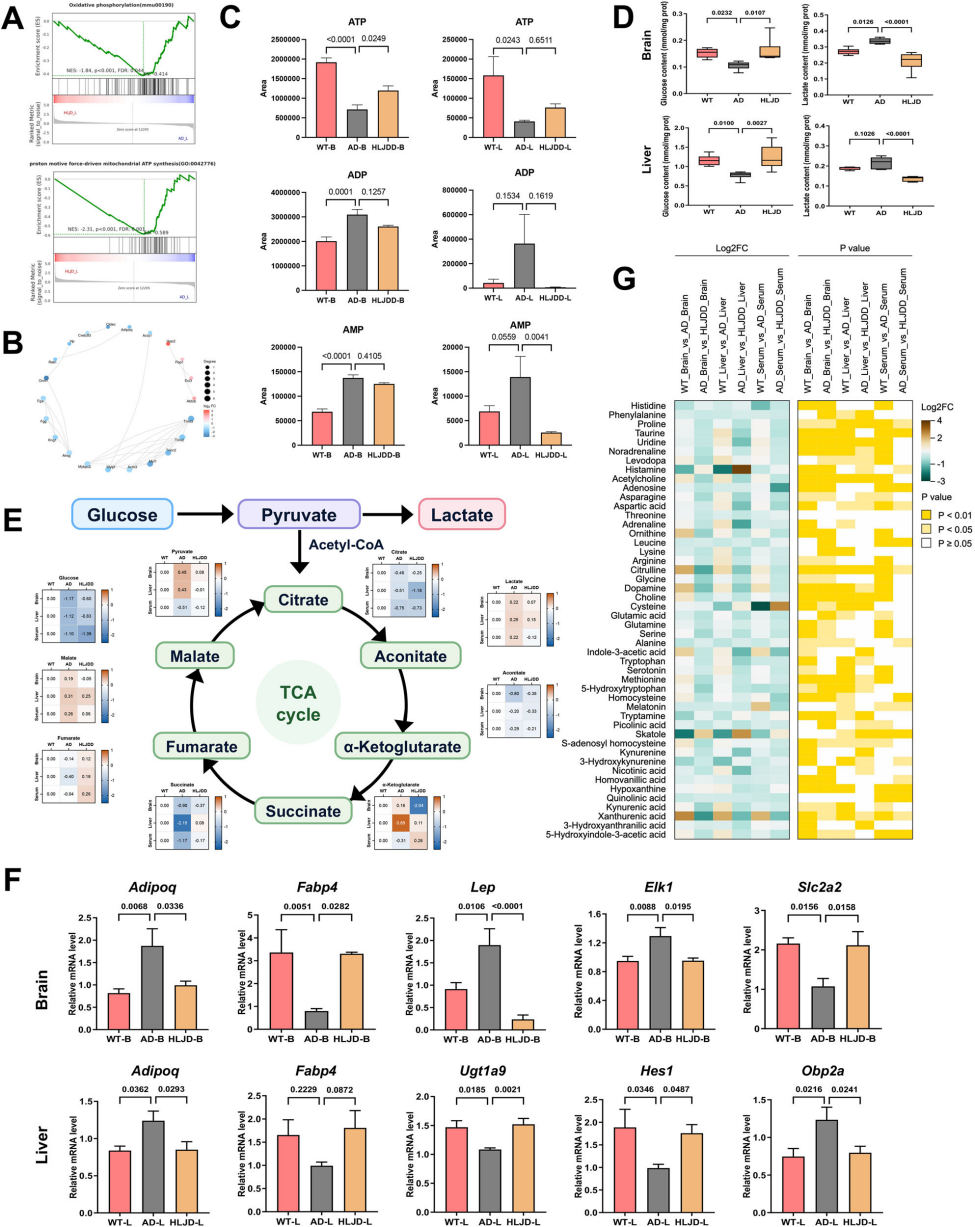
Effects of HLJDD on energy metabolism and metabolism‐related gene expression in 5xFAD mice. (A) Gene set enrichment analysis (GSEA) of liver transcriptomic data comparing the AD and HLJDD groups. (B) Protein‐protein interaction (PPI) network of selected metabolism‐related proteins. Nodes represent proteins, and edges represent interactions between proteins. (C) ATP, ADP, and AMP signals in brain and liver tissues measured by targeted metabolite analysis and expressed as peak areas (*n* = 6 per group). (D) Glucose and lactate contents in brain and liver tissues determined using biochemical assays (*n* = 6 per group). (E) Schematic representation of glycolysis and the tricarboxylic acid (TCA) cycle, accompanied by heatmaps showing the indicated metabolites in brain, liver, and serum across the WT, AD, and HLJDD groups (*n* = 6 per group). (F) Relative mRNA expression of the indicated genes in brain and liver tissues measured by quantitative PCR (*n* = 3 per group). (G) Integrated heatmaps showing log_2_ fold changes and corresponding *p‐*values for the indicated comparisons in brain, liver, and serum. Rows represent metabolites, and columns represent the indicated comparisons. Colors follow the respective legends (*n* = 6 per group). Bar plots show mean ± SEM. *p‐*Values are indicated in the panels. WT, wild‐type mice; AD, 5xFAD model mice; HLJDD, HLJDD‐treated 5xFAD mice.

To elucidate the therapeutic mechanism of HLJDD, we measured metabolites involved in central carbon metabolism—encompassing the pentose phosphate pathway, tricarboxylic acid (TCA) cycle, and glycolysis. Brain tissue metabolomics revealed HLJDD's significant modulation of central carbon metabolites in 5xFAD mice, including changes in NAD+, succinate, AMP, 3‐phosphoglycerate, and citraconate levels (Figure 9C,E). In liver tissue, HLJDD altered citrate, aconitate, and succinate levels. Notably, succinate—a key immunometabolite modulating innate immunity through IL‐1β production and critically linked to oxidative stress and energy metabolism—was synchronously normalized by HLJDD in both brain and liver tissues. This regulation underscores HLJDD's systemic restoration of metabolic homeostasis (Figures S9–S11).

### 2.11 HLJDD Regulates the PPAR‐γ, PI3K/Akt, NF‐κB Signaling Pathway in the Brain and Liver

Gene expression analysis via heatmap revealed a clear distinction in the PPAR signaling pathway between the WT and AD groups (Figure 10A). Peroxisome proliferator‐activated receptor gamma (PPAR‐γ) was a key modulator of inflammation, playing an indispensable role, particularly in promoting an anti‐inflammatory microglial phenotype. Based on the exploratory multi‐omics findings, PPAR‐γ and selected components of PI3K/Akt and NF‐κB signaling were further examined using targeted molecular and histological assays.

**FIGURE 10:**
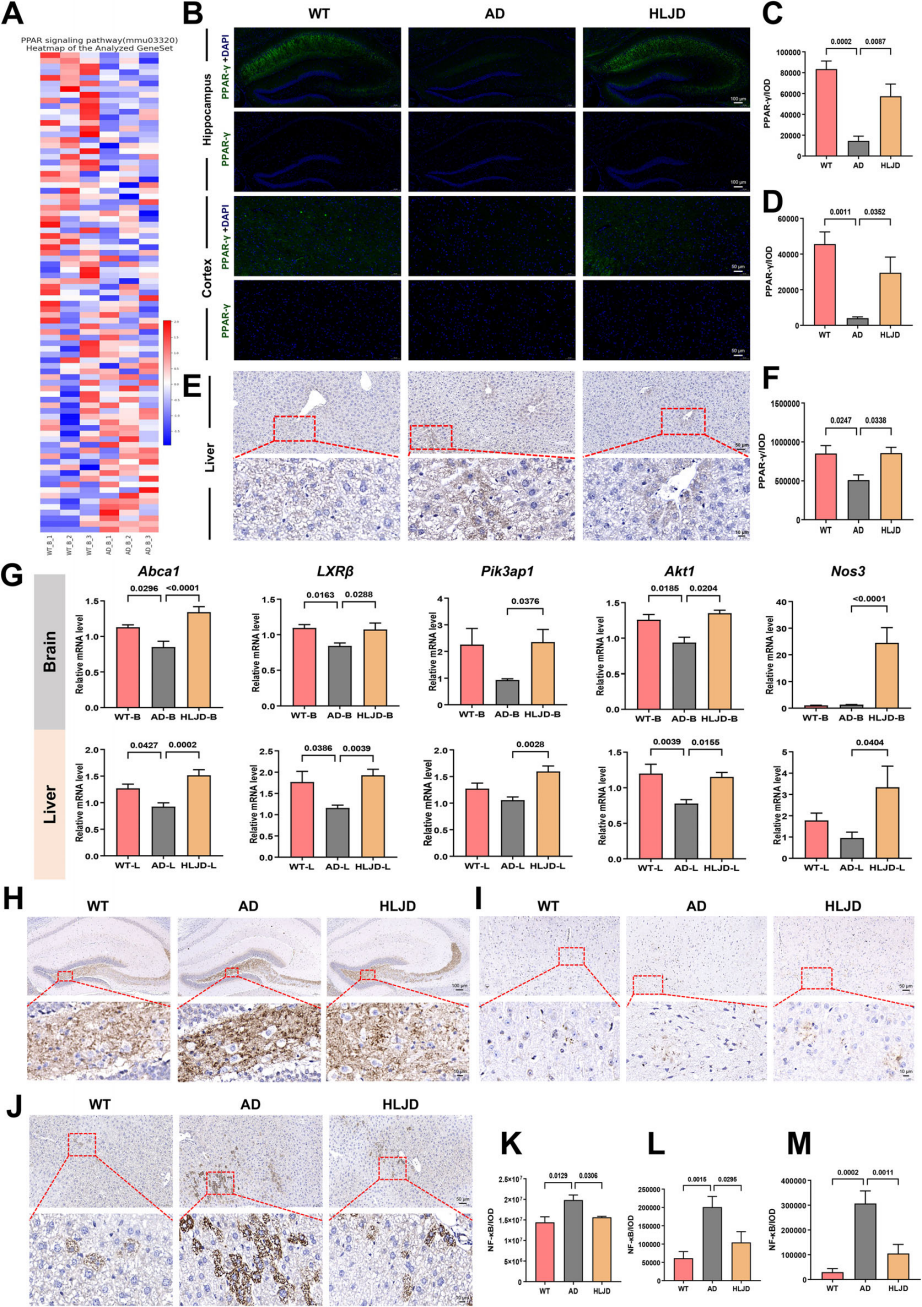
Effects of HLJDD on PPAR‐γ, PI3K/Akt, and NF‐κB signaling. (A) Heatmap showing the expression patterns of genes associated with the PPAR signaling pathway. (B) Representative PPAR‐γ immunofluorescence images of the hippocampus (Scale bar = 100 µm) and cortex (Scale bar = 50 µm) of the brain tissue. PPAR‐γ is shown in green, and nuclei are counterstained with DAPI (blue). (C, D) Quantification of PPAR‐γ immunofluorescence in hippocampus (C) and cortex (D) (*n* = 3 mice/group; each *n* represents the average of three technical replicates). (E) Representative immunohistochemical images of PPAR‐γ expression in liver tissue (Scale bar = 50 µm and 10 µm). (F) Quantification of PPAR‐γ immunohistochemical staining in the liver (*n* = 3). (G) Relative expression levels of genes associated with the PI3k/AKT signaling pathway in brain and liver tissues of 5xFAD mice following HLJDD treatment (*n* = 3). (H–J) Representative immunohistochemical images of NF‐κB expression in the hippocampus (Scale bar = 100 µm and 10 µm) (H), cortex (Scale bar = 50 µm and 10 µm) (I), and liver (Scale bar = 50 µm and 10 µm) (J). (K–M) Quantification of NF‐κB immunohistochemical staining in the hippocampus (K), cortex (L), and liver (M) (*n* = 3 mice/group; each *n* represents the average of three technical replicates).

Immunofluorescence results demonstrated significantly reduced PPARγ‐positive staining intensity in the hippocampal and cortical regions of the AD group compared to the WT group (Figure 10B–D). HLJDD intervention effectively restored PPAR‐γ expression in these brain regions. Furthermore, the results revealed markedly decreased PPAR‐γ staining intensity in the liver tissue of the AD group, which was significantly increased following HLJDD treatment (Figure 10E,F). Collectively, immunohistochemical and immunofluorescence analyses showed that the reduced PPAR‐γ expression observed in 5xFAD mice was partially restored following HLJDD treatment in both brain and liver tissues.

Building upon transcriptomic and metabolomic findings, we focused our investigation on the PPAR‐γ and PI3K/Akt signaling pathway. RT‐PCR validation of key pathway genes demonstrated that HLJDD not only increased the expression of *Abca1* and *Lxrβ*, but also significantly upregulated the levels of *Pik3ap1*, *Akt1*, and *Nos3* genes in both brain and liver tissues (Figure 10G).

Independent research indicated that mitochondrial dysfunction elevated the production of reactive oxygen species (ROS). ROS could activate critical signaling pathways, including NF‐κB. Our immunohistochemical analysis revealed that HLJDD effectively reduced NF‐κB expression in both brain and liver tissues (Figure 10H–M). These findings indicated that HLJDD treatment was associated with coordinated alterations in PPAR‐γ, PI3K/Akt, and NF‐κB signaling in both brain and liver tissues.

### 2.12 HLJDD Attenuates Inflammatory Responses in an AML12‐BV2 Conditioned‐Medium Model with Possible Involvement of Hepatocytes

Cross‐organ multi‐omics profiling and tissue‐level validation identified PPAR‐related immune‐metabolic alterations in both the liver and brain of 5xFAD mice. Additional pathway scoring showed tissue‐ and omics‐dependent directional changes in PPAR‐, PI3K‐AKT‐mTOR‐, and inflammation‐related signatures following HLJDD treatment (Figure S12A,B). Exploratory ligand‐receptor and serum‐metabolite analyses further nominated potential links between liver and brain molecular responses (Figure S12C–F). To investigate whether the hepatic response to HLJDD could influence microglial inflammation, we established an AML12 hepatocyte‐BV2 microglial cell conditioned‐medium model. On the basis of a CCK‐8 assessment, HLJDD concentrations of 2.5, 7.5, and 25 µg/mL were selected for the cell experiments. In LPS‐challenged AML12 cells, HLJDD reduced the release of TNF‐α, IL‐1β, and IL‐6 in a concentration‐dependent manner (Figure 11A). Immunoblot analysis further showed increased PPAR‐γ expression and decreased iNOS, p‐AKT, and p‐NF‐κB p65 signals after HLJDD treatment (Figure 11B). These findings indicate that the anti‐inflammatory effects associated with HLJDD treatment in hepatocytes were accompanied by alterations in PPAR‐γ expression and AKT/NF‐κB signaling.

**FIGURE 11:**
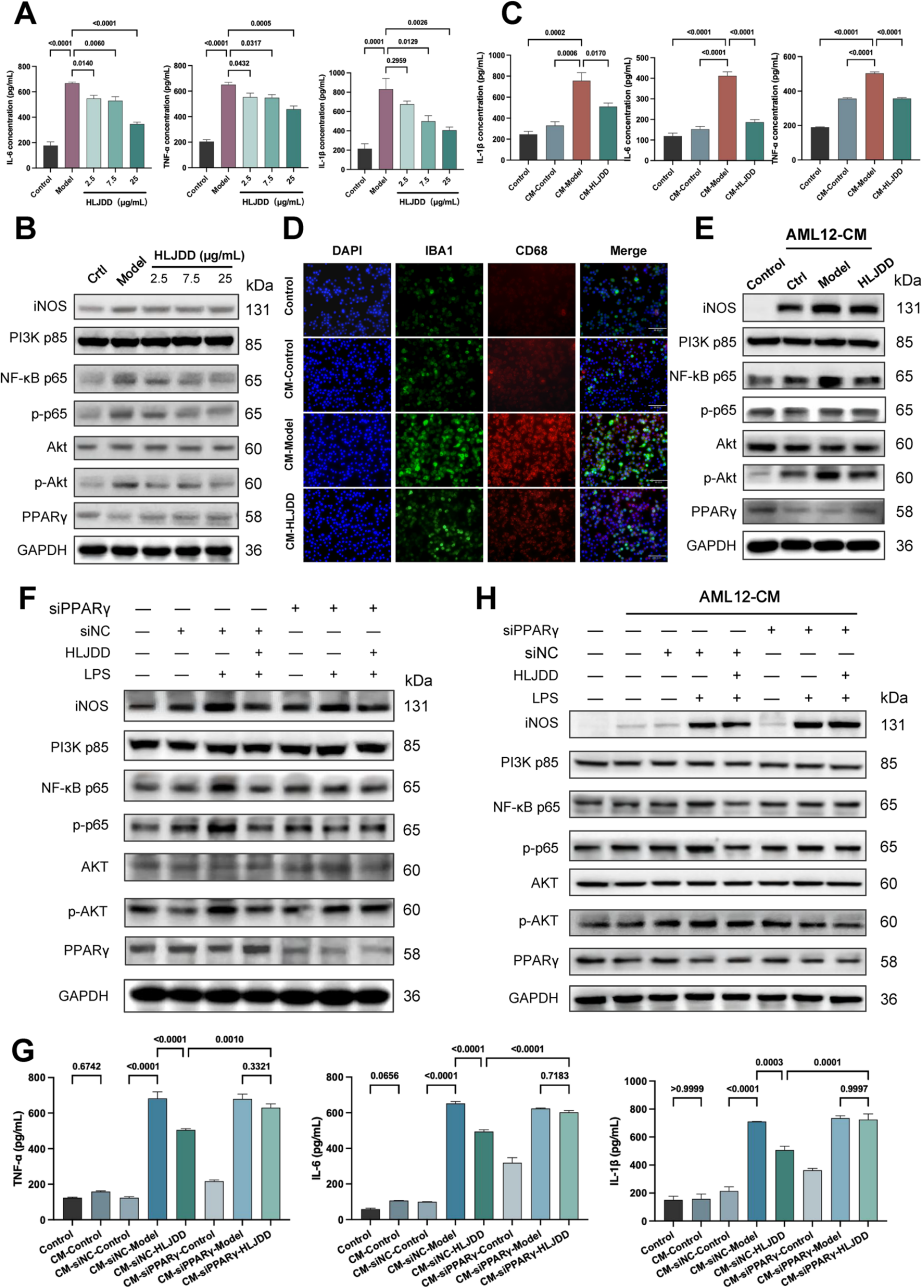
Hepatocyte PPAR‐γ contributes to HLJDD‐associated attenuation of inflammatory responses in the AML12‐BV2 conditioned‐medium model. (A) TNF‐α, IL‐1β, and IL‐6 concentrations in AML12 culture supernatants measured by ELISA. AML12 cells were treated with LPS (0.5 µg/mL) alone or simultaneously with HLJDD (2.5, 7.5, or 25 µg/mL) for 24 h (*n* = 3). (B) Representative western blots of PPAR‐γ, PI3K p85, AKT, p‐AKT, NF‐κB p65, p‐p65, and iNOS in AML12 cells from the control group, model group, and HLJDD group. (C) TNF‐α, IL‐1β, and IL‐6 concentrations in BV2 culture supernatants after 24 h of exposure to AML12‐derived conditioned medium (CM), measured by ELISA (*n* = 3). (D) Representative immunofluorescence images of IBA1 (green) and CD68 (red) in BV2 cells from the indicated groups. Nuclei were counterstained with DAPI (blue). Scale bars: 90 µm. Immunofluorescence staining was performed in a single experiment with five replicate wells per condition. (E) Representative western blots of PPAR‐γ, PI3K p85, AKT, p‐AKT, NF‐κB p65, p‐p65, and iNOS in BV2 cells from the Control, CM‐Control, CM‐Model, and CM‐HLJDD groups. (F) Representative western blots of the indicated proteins in AML12 cells following transfection with PPAR‐γ‐targeting siRNA (siPPAR‐γ) or nontargeting control siRNA (siNC) and subsequent treatment as indicated. (G) TNF‐α, IL‐1β, and IL‐6 concentrations in BV2 cells treated with conditioned media derived from AML12 cells transfected with siPPAR‐γ or siNC (*n* = 3). (H) Representative western blots of PPAR‐γ, PI3K p85, AKT, p‐AKT, NF‐κB p65, p‐p65, and iNOS in BV2 cells treated with conditioned media derived from AML12 cells transfected with siPPAR‐γ or siNC. Except for the immunofluorescence assay in D, the experiments shown were performed three times independently. Quantitative data are presented as mean ± SEM.

To determine whether HLJDD‐associated changes in hepatocytes were accompanied by altered microglial responses, conditioned medium (CM) from the indicated AML12 treatment groups was transferred to BV2 cells. Relative to CM from untreated AML12 cells (CM‐Control), CM from LPS‐challenged hepatocytes (CM‐Model) increased BV2 secretion of TNF‐α, IL‐1β, and IL‐6 (Figure 11C). It also increased IBA1 and CD68 immunofluorescence signals and was associated with greater iNOS expression and AKT and NF‐κB p65 phosphorylation, alongside lower PPAR‐γ expression (Figure 11D,E). In contrast, CM from HLJDD‐treated, LPS‐challenged hepatocytes (CM‐HLJDD) attenuated cytokine release and the microglial inflammatory‐marker response. These changes were accompanied by higher BV2 PPAR‐γ abundance and lower iNOS, p‐AKT, and p‐p65 signals than those observed with CM‐Model (Figure 11C–E). Thus, HLJDD treatment of hepatocytes was associated with a less pro‐inflammatory response in recipient microglia.

To test the contribution of hepatocyte PPAR‐γ to these effects, we silenced PPAR‐γ in AML12 cells before LPS challenge and HLJDD treatment. In cells transfected with non‐targeting siRNA, HLJDD increased PPAR‐γ expression, while iNOS abundance and the p‐AKT/AKT and p‐p65/p65 ratios showed a trend toward reduction relative to the siNC‐Model group (Figure 11F). PPAR‐γ knockdown attenuated these HLJDD‐associated changes, with higher iNOS expression and AKT and p65 phosphorylation than in the siNC‐HLJDD group (Figure 11F). These results implicate hepatocyte PPAR‐γ in the regulation of inflammatory signaling by HLJDD.

We next examined whether silencing PPAR‐γ in donor hepatocytes altered the response of recipient BV2 cells. Compared with CM‐siNC‐Model, CM‐siNC‐HLJDD decreased BV2 release of TNF‐α, IL‐1β, and IL‐6 and was associated with increased PPAR‐γ expression, reduced iNOS abundance, and lower p‐AKT/AKT and p‐p65/p65 ratios (Figure 11G,H). When PPAR‐γ was silenced in the donor AML12 cells, the ability of HLJDD‐associated CM to limit BV2 cytokine release and inflammatory signaling was weakened (Figure 11G,H). Together, these conditioned‐medium and knockdown results indicate that hepatocyte PPAR‐γ contributes, at least in part, to the suppression of microglial inflammatory responses associated with HLJDD treatment. These findings complement the liver‐brain immune‐metabolic changes observed in 5xFAD mice.

## 3 Discussion

AD is a neurodegenerative disorder characterized by progressive cognitive decline. In 5xFAD mice, mutant APP and PS1 overexpression induced Aβ accumulation and neuronal degeneration. The major finding of this study was the marked pathological convergence between brain and liver in 5xFAD mice. Integrated transcriptomic, proteomic, phosphoproteomic, and metabolomic analyses identified PPAR signaling and insulin‐related pathways as central nodes of dysregulation in both organs. The brain displayed a neuroinflammatory phenotype marked by microglial Itgax/CD11c and astrocytic GFAP activation, whereas the liver underwent major reprogramming of energy and lipid metabolism. These data support the view that AD is associated with coordinated central and peripheral metabolic alterations involving both the brain and liver.

Multi‐omics integration further showed coordinated inflammatory and metabolic abnormalities across tissues. Serum metabolomics revealed amino acid and lipid disturbances that paralleled tissue‐specific defects in insulin sensitivity and energy homeostasis. Phosphoproteomic analysis showed altered phosphorylation of IRS family members in the brain and Insr Ser1054 in the liver, consistent with dysregulation of upstream insulin‐related signaling. This pattern would suppress PI3K/Akt and downstream effectors such as mTOR and AMPK, suggesting that insulin resistance in this model involves post‐translational rewiring rather than ligand deficiency alone. Concurrent phosphorylation‐level activation of NF‐κB components in both tissues further supports a reciprocal relationship between inflammation and metabolic resistance. Integration of transcriptomics and metabolomics also defined a metabolic‐immune nexus: increased Itgax expression and Aβ‐adjacent glial activation coincided with purine and lipid disturbances, implying that immune dysfunction both responds to and aggravates a failing metabolic microenvironment. Enrichment of phosphatidylinositol signaling at the phosphorylation level may also explain impaired phagocytosis and defective Aβ clearance. Together, these data support the “Type 3 diabetes” hypothesis of AD while extending it to a synchronized multi‐organ process.

Behavioral and histopathological findings were consistent with these molecular changes. Compared with WT mice, 5xFAD mice had lower body weight, impaired spatial learning and memory, and increased anxiety‐like behavior. Although brain weight did not differ significantly, lower terminal body weight in the AD group increased the brain‐to‐body weight ratio. Histology and immunohistochemistry confirmed neuronal injury and increased Aβ‐ and PS1‐associated pathology. HLJDD, particularly at the high dose, and Donepezil improved behavioral performance, and ameliorated neuronal degeneration. High‐dose HLJDD also reduced plaque deposition, and abnormal Aβ and PS1 expression. Some outcomes did not reach statistical significance, likely due to sample size, dose, or treatment duration, but the overall pattern supported therapeutic benefit. The high‐dose HLJDD group, approximately one‐tenth of the human clinical dose, showed stronger efficacy than the low‐dose group, consistent with our previous APP/PS1 study [14].

Immune dysregulation was a major feature of the model group. Brain transcriptomics showed that DEGs between WT and AD mice were enriched mainly in immune and inflammatory pathways, consistent with evidence implicating immune dysfunction in AD pathogenesis [16]. DEGs between HLJDD and AD mice were enriched in lymphocyte chemotaxis, suggesting that altered chemokine signaling may affect immune‐cell distribution in the brain and influence disease progression [17]. Although inflammation is unlikely to be the primary etiology of AD, immune processes clearly contribute to pathology, as immunoglobulins have been identified within senile plaques [18]. Activated microglia can promote tau spread and initiate inflammatory cascades that exacerbate neurodegeneration [19, 20], and the observation that anti‐inflammatory drugs may delay AD onset further supports their pathogenic importance [21]. In this study, multiplex immunofluorescence showed colocalization of Aβ, IBA1, and GFAP in the hippocampus and cortex, whereas HLJDD reduced positive staining for all three markers. At the gene level, *Ly86*, *Cd68*, and *Itgax* were significantly upregulated in the AD brain [22, 23] and downregulated by HLJDD. Pro‐inflammatory cytokines, including TNFα, IFNγ, IL‐12A, IL‐1β, and IL‐6, were elevated in both brain and liver in the AD group and reduced after HLJDD intervention, reinforcing the importance of systemic inflammation in AD pathophysiology.

PPAR signaling emerged as one of the pathways concurrently altered in the brain and liver. PPARs are nuclear receptors involved in the regulation of metabolism, differentiation, inflammation, and oxidative stress [24]. Transcriptomic analysis showed enrichment of PPAR‐related signaling after HLJDD treatment, while previous studies have demonstrated that PPAR agonists can attenuate inflammation and oxidative stress and ameliorate AD‐related pathology [25]. In the liver, PPAR‐α and PPAR‐γ are important regulators of lipid and glucose homeostasis, and disruption of PPAR signaling may contribute to systemic metabolic abnormalities [26]. The parallel enrichment of PPAR‐related genes in brain and liver therefore suggests coordinated central and peripheral alterations rather than establishing direct inter‐organ communication. PPAR‐γ also has neuroprotective and anti‐inflammatory functions; for example, rosiglitazone inhibits LPS‐induced microglial activation [27]. In this study, PPAR‐γ expression was reduced in both the brain and liver of 5xFAD mice compared with WT controls and was partially restored following HLJDD treatment.

This pathway also intersected with cholesterol homeostasis. PPAR‐γ regulates genes involved in cholesterol efflux, including Abca1 and Abcg1 [28]. Here, Abca1 and Lxrβ were significantly reduced in both the brain and liver of the AD group. ABCA1 is essential for reverse cholesterol transport [29], whereas LXRβ functions as a cholesterol sensor and regulator of inflammatory responses [30]. Their downregulation would be expected to promote intracellular cholesterol accumulation, membrane dysfunction, neuronal injury, and inflammatory signaling. HLJDD significantly upregulated Abca1 and Lxrβ, suggesting that improved cholesterol transport contributes to its protective effect. In parallel, HLJDD enhanced expression of Pik3ap1, Akt1, and Nos3 in brain and liver, indicating changes of genes related to the PI3K/Akt pathway, which is central to cell survival, metabolism, and glucose/lipid homeostasis [31].

The insulin‐resistance phenotype identified by multi‐omics integration was further supported by transcriptomic and metabolomic changes. Serum metabolomics showed that DEMs between AD and WT mice were concentrated in energy‐metabolism pathways associated with insulin resistance, whereas HLJDD affected glycolysis, insulin resistance, and lipid metabolism more broadly. Brain DEGs between HLJDD and AD mice were enriched in insulin‐resistance pathways, and liver DEGs were enriched in diabetes‐related signaling. These findings are highly relevant because insulin resistance promotes tau hyperphosphorylation, exacerbates Aβ neurotoxicity, impairs cerebral glucose metabolism, and accelerates neuronal loss [32]. This interpretation is supported by evidence that individuals with type 2 diabetes have increased AD risk and that markers of brain insulin resistance rise early in disease progression [33, 34]. Adipoq was upregulated, whereas Fabp4, which supports fatty‐acid transport and neuronal energy homeostasis, was downregulated; HLJDD reversed these trends [35]. HLJDD also reduced Lep, Elk1, and Csf3‐related signaling, suggesting partial restoration of energy balance and neuroinflammatory homeostasis.

Metabolic pathway analysis further revealed coordinated metabolic alterations in the brain and liver. In the AD group, lipid, amino acid, and energy metabolism showed marked alterations, consistent with previous reports of abnormal cholesterol and phospholipid metabolism [36], dysregulated amino acid metabolism affecting neurotransmitter synthesis [37], and early metabolic disturbances that precede overt disease [38]. Impaired brain glucose metabolism has also been linked to Aβ accumulation [39]. HLJDD upregulated genes associated with energy metabolism and cellular homeostasis, including Pck1, Fbp1, and Atp2a1, suggesting improved metabolic flexibility, calcium handling, and neuronal survival [40]. Common enrichment of purine metabolism and vitamin B6 metabolism in brain and liver further indicated a shared pathological network. Targeted metabolomics provided additional support for an energy‐centered mechanism: HLJDD regulated TCA‐cycle metabolites in both brain and liver, modulated phenylalanine, asparagine, 5‐hydroxytryptophan, and melatonin, and increased succinate in both tissues. Because pro‐inflammatory cytokines can promote glutamine uptake and succinate accumulation, and inflammatory mitochondrial reprogramming can further promote succinate‐linked cytokine production, this finding likely reflects close coupling between energy metabolism and immune activation [41, 42]. The multi‐omics analyses in the present study were exploratory and hypothesis‐generating. Accordingly, pathway enrichment was interpreted as reflecting coordinated molecular alterations rather than direct pathway activation or causal involvement. Subsequent targeted analyses, including qRT‐PCR, biochemical assays, immunohistochemistry, immunofluorescence, and targeted metabolomics, provided additional support for selected omics‐derived findings. Although the low‐dose HLJDD and donepezil groups were included for pharmacodynamic comparison and positive‐control evaluation, full multi‐omics analyses were performed mainly on the AD model group and the high‐dose HLJDD group; therefore, further studies incorporating comparable multi‐omics analyses across all treatment groups are needed to more comprehensively define the active components and tissue‐specific mechanisms of HLJDD.

Several limitations of the present study should be acknowledged. First, only male mice were included, limiting the generalizability of the findings to females. Future studies should include both sexes to assess potential sex‐dependent differences in disease phenotypes and treatment responses. Second, unequal group sizes and relatively small numbers of biological replicates in some omics and validation analyses may have limited statistical power and precision. In addition, the four‐day MWM acquisition design may have limited sensitivity for detecting later‐stage or asymptotic differences in spatial learning compared with longer training paradigms. Future studies should incorporate prospective sample‐size calculations, more balanced group allocation where feasible, and extended behavioral training paradigms when appropriate. Third, the multi‐omics findings were exploratory, and pathway enrichment and parallel brain‐liver alterations do not establish pathway activation or causal interorgan relationships. The absence of equivalent multi‐omics profiling across all treatment groups also limits comparisons of dose‐related responses and treatment mechanisms. Fourth, the conditioned‐medium experiments support hepatocyte‐to‐microglia effects in vitro and suggest possible involvement of hepatocyte PPAR‐γ, but the responsible mediators and their causal contribution in vivo remain unresolved. Liver‐specific interventions are needed to clarify their relevance to HLJDD‐associated neuroprotection, while the cellular immunofluorescence findings require confirmation in independent experiments. Finally, the contributions of individual HLJDD constituents and their interactions remain unclear, and tissue metabolite measurements at single time points cannot establish metabolic flux or temporal relationships. Constituent‐level experiments, longitudinal profiling, and metabolic tracing are needed to address these questions.

This study integrated multi‐omics profiling with behavioral, histopathological, and cellular analyses to characterize brain and liver alterations in 5xFAD mice and their responses to HLJDD. HLJDD improved cognitive performance and neuropathological outcomes, accompanied by changes in inflammatory signaling and metabolic profiles in both tissues. Complementary conditioned‐medium experiments showed that HLJDD treatment of LPS‐exposed hepatocytes modified subsequent inflammatory responses in BV2 cells, with knockdown experiments suggesting possible involvement of hepatocyte PPAR‐γ. Together, these findings connect treatment‐responsive brain and liver alterations with functional evidence of hepatocyte‐to‐microglia effects in vitro. They support further investigation of hepatic contributions to HLJDD‐associated neuroprotection, while the mediators and in vivo relevance of these cellular effects remain to be established.

## 4 Experimental Section

### 4.1 Materials

Scutellaria baicalensis (1912002, Shanxi), Coptis chinensis (2001001, Sichuan), Phellodendron amurense (2001001, Sichuan), and Gardenia jasminoides (1909001, Jiangxi) were all found to have met the relevant regulations of the 2020 edition of the Pharmacopoeia of the People's Republic of China. 1 kg of Scutellaria baicalensis, 1.5 kg of Coptis chinensis, 1 kg of Phellodendron amurense, and 1.5 kg of Gardenia jasminoides were taken and crushed into the coarsest powder. The mixture was soaked in 50 L of distilled water for 3 h and decocted for 1.5 h. The residue was re‐boiled in 40 L of water for another 1.5 h after filtration. The supernatant was taken to be concentrated. It was dried under reduced pressure at 80°C until dry. A total of 1700 g of Huanglian Jiedu Decoction powder was obtained with a yield of 34%. It was crushed to obtain HLJDD powder. Donepezil hydrochloride tablets 10 mg (production batch number: 2109110) were purchased from Eisai Pharmaceutical Co., Ltd. (Japan). MS‐grade formic acid, methanol, and acetonitrile were obtained from Thermo Fisher Scientific Inc. (USA). Wahaha pure water was purchased from Hangzhou Wahaha Group Co., Ltd. (China).

### 4.2 Animals and Treatments

Fifty‐three 4‐month‐old male 5xFAD transgenic mice were purchased from Shanghai Southern Model Organisms Co., Ltd. with the animal production license number SCXK (Hu) 2019‐0002. Fifteen 4‐month‐old male C57BL/6J wild‐type (WT) mice were purchased from Beijing Vital River Laboratory Animal Technology Co., Ltd. with the animal production license number SCXK (Jing) 2021‐0006. The WT control mice were age‐matched and genetically matched to the 5xFAD mice on the C57BL/6 genetic background to minimize potential confounding effects caused by genetic background differences. The experimental animals were all reared in the specific pathogen‐free (SPF) animal house of the Experimental Animal Center of the Basic Research Institute of the Chinese Academy of Medical Sciences. During the experiment, the animals were fed ordinary feed under the conditions of 12 h intermittent lighting during the day and night, and constant temperature and humidity (20 ± 1°C, 50 ± 10%). The bedding was replaced regularly, and the animals were fed adaptively for one week before drug administration. The animal ethics approval number was IACUC‐2022100811. WT mice served as the control group (WT, *n* = 15). The 5xFAD mice were randomly divided into four groups: the model group (AD, *n* = 16), the high‐dose Huanglian Jiedu group (H‐H, *n* = 16, 116 mg·kg^−1^·d^−1^), the low‐dose Huanglian Jiedu group (H‐L, *n* = 11, 58 mg·kg^−1^·d^−1^), and the positive drug donepezil group (Don, *n* = 10, 1 mg·kg^−1^·d^−1^). Both the WT and AD groups were given equal amounts of normal saline. The body weight of each mouse was measured weekly, and the administration was continued for 10 weeks.

Male C57BL/6 mice were randomly allocated to the vehicle control, clinically equivalent‐dose HLJDD, and high‐dose HLJDD groups (*n* = 6 per group). Following a single oral administration, blood was collected at 2 h after dosing. The animals were subsequently transcardially perfused with pre‐chilled heparinized PBS, and the liver and brain tissues were harvested to minimize residual blood contamination. Plasma, liver, and brain samples were analyzed by LC MS. HLJDD‐derived constituents were putatively identified based on retention time, accurate precursor mass, mass error, and MS/MS fragment ions, together with database and literature matching. Compound signals were evaluated against those in vehicle control samples. All animal procedures were approved by the Animal Ethics Committee (Ethics approval No. 2026B189).

### 4.3 Behavioral Evaluation

The Morris water maze (MWM) was conducted with the JLBehv‐MWMG Universal Morris water maze video analysis system (Shanghai Jiliang Software Science & Technology Co., Ltd., China). The MWM experiment consisted of a four‐day place‐navigation acquisition phase (days 1–4) followed by a platform‐free spatial probe trial on day 5. During the acquisition phase, a circular platform (10 cm in diameter) was positioned 20 cm from the pool wall in a designated target quadrant and submerged approximately 1–2 cm below the water surface. The platform location remained unchanged throughout the four‐day acquisition phase. The walls of the pool and the hidden platform were painted white, and the water was dyed with a food whitener to create a white background. The water temperature was maintained at 20–22°C. Opaque blue curtains were hung around the perimeter to block out light, and different shaped patterns were attached to the curtains as landmarks. During each of the four consecutive acquisition days, each mouse underwent four trials, with one trial initiated from each of the four quadrants. The starting position varied among trials, whereas the platform remained fixed in the designated target quadrant throughout acquisition. The trials were spaced 15–20 min apart and lasted for 60 s each. Once a mouse found the platform, it was allowed to stay for 15 s. If the mouse failed to find or climb onto the platform within 60 s of entering the water, it was placed on the platform for 20 s. The average latency time of the four trials each day was used as a measure of the mouse's learning performance. For the spatial probe test on day 5, the platform was removed, and each mouse was released from the quadrant opposite the former platform location, facing the pool wall. Swimming behavior was observed for 60 s.

The elevated plus maze (EPM) was conducted with the DigBehv‐EPMG elevated plus maze (Shanghai Jiliang Software Science & Technology Co., Ltd., China), consisting of two open arms and two closed arms intersecting perpendicularly in a cruciform shape. At the start of the experiment, the mouse was placed in the center of the maze facing one of the open arms, and its behavior was recorded for 5 min. After the experiment, the mouse was removed, and the arms were cleaned and sprayed with alcohol to remove any scent.

The Open Field Test (OFT) was conducted with the JLBehv‐LA Universal video analysis system (Shanghai Jiliang Software Science & Technology Co., Ltd., China). Mice were randomly placed in the center of an open box with sides and a bottom measuring 50 cm in length, all painted white, and walls 50 cm high. Their movement trajectory was recorded for 5 min. The number of fecal pellets produced by each mouse during the 5‐min experiment was manually recorded. After 5 min, the mice were removed, and the box was cleaned and sprayed with alcohol to remove any scent.

The ANY maze behavioral analysis system for small animals (Stoelting Co., USA) was utilized for data analysis. Behavioral parameters were automatically recorded and calculated using the corresponding tracking software.

### 4.4 Tissue Collection

The mice were anesthetized with 10% chloral hydrate, and blood samples were collected from the orbit. After standing for 2 h at room temperature, the blood samples were centrifuged at 3000 rpm for 15 min at 4°C. The serum was transferred, aliquoted, and stored at −80°C.

The skulls of the mice were cut at the connection with the cervical vertebrae. Using ophthalmic scissors, the skull was opened from the occipital foramen upward, and the brain was separated, weighed, washed with saline solution, and the surface water was dried with qualitative filter paper. Three samples from each group were randomly selected and fixed in 4% formaldehyde solution. The liver and spleen tissues of the mice were also removed and weighed.

### 4.5 Histological Assays

Tissues were fixed in 4% paraformaldehyde, dehydrated using gradient ethanol, cleared in xylene, embedded in paraffin, and sectioned. The paraffin sections were subsequently dewaxed using a dewaxing solution and ethanol of different concentrations. Hematoxylin‐eosin (H&E) and Congo red staining were then performed. Hematoxylin staining solution (G1140), eosin staining solution (G1100), and Congo red staining solution (G1531) were purchased from Beijing Solarbio Science & Technology Co., Ltd. Antigen retrieval was conducted using citrate buffer (PH 6.0), and endogenous enzyme activity was blocked by incubating the sections with 3% H_2_O_2_ solution. After adding serum and incubating at 37°C, immunohistochemical staining and immunofluorescence staining were performed. For quantitative image analysis, predefined regions of interest, including the hippocampal and cortical regions, were analyzed using consistent thresholding parameters across all groups. For each group, three animals were included. Three representative fields per section were analyzed, and the mean value of the fields from each animal was used as one biological replicate for statistical comparison. Image quantification was performed by investigators blinded to group allocation. H&E staining was used for representative morphological assessment and was not subjected to quantitative analysis.

### 4.6 Immunohistochemical Staining

The following antibodies were used: anti‐Presenilin 1/PS‐1 antibody (1:100, Abcam, EP2000Y, ab76083), anti‐beta Amyloid 1–42 antibody (1:1000, Abcam, mOC98, ab201061), NF‐κB p65 (1:1000, Cell Signaling Technology, 8242), and PPAR gamma Rabbit pAb (1:200, Chengdu Zhengneng Biotechnology Co., Ltd., 340844). These primary antibodies were incubated overnight at 4°C. Subsequently, an HRP‐labeled secondary antibody (Beijing Solarbio Science & Technology Co., Ltd., PN0046) was incubated for 1 h at 37°C, followed by three washes with PBST. The sections were developed using DAB working solution, counterstained with hematoxylin, differentiated with 0.5% hydrochloric acid‐alcohol solution, and rinsed in running water after immersion in the blue back liquid. After dehydration, the sections were mounted with neutral balsam.

### 4.7 Immunofluorescence Staining

#### 4.7.1 Tissue Immunofluorescence

The following antibodies were used: anti‐beta Amyloid 1–42 antibody (1:2000, Abcam, mOC98, ab201061), anti‐IBA1 antibody (1:2000, Abcam, EPR16589, ab178847), GFAP Polyclonal antibody (1:2000, Proteintech, 16825‐1‐AP), and anti‐PPAR‐γ antibody (1:200; Cat. No. 340844; ZEN‐BIOSCIENCE, Chengdu, China). Aβ, IBA1, GFAP, and PPAR‐γ signals were visualized using TSA‐CY5, TSA‐CY3, TSA‐488, and TSA‐488, respectively. The primary antibodies were incubated overnight at 4°C. An HRP‐labeled secondary antibody was incubated for 1 h at 37°C. CY3 tyramide was then added to the tissue, and TSA incubation was performed at 37°C. Antigen retrieval and serum blocking were subsequently conducted. DAPI working solution was applied to the tissue. After drying, an anti‐fluorescence mounting medium was added to the tissue. The sections were recorded and analyzed using a scanner.

#### 4.7.2 Cell Immunofluorescence Staining

BV2 cells were fixed with 4% paraformaldehyde (P0099, Beyotime, China) at room temperature for 30 min, washed with PBS, and then treated with 0.1% Triton X‐100 (P0096, Beyotime, China) for 15 min to permeabilize the cell membranes. The cells were incubated with an immunofluorescence blocking solution (P0102, Beyotime, China) for 1 h at room temperature; after washing with PBS, they were incubated with primary antibodies against IBA1 (ab178846, Abcam, UK) and CD68 (ab53444, Abcam, UK) overnight at 4°C. Following washing, the cells were incubated with secondary antibodies: Goat anti‐Rabbit IgG (H+L) Highly Cross‐Adsorbed Secondary Antibody, Alexa FluorTM 488 (a‐11034, Thermo Fisher Scientific, China) and Goat anti‐Rat IgG (H+L) Cross‐Adsorbed Secondary Antibody, Alexa FluorTM 594(a‐11007, Thermo Fisher Scientific, China) at room temperature in the dark for 1 h. After washing with PBS, the cells were stained for nuclei using 4′,6‐diamidino‐2‐phenylindole (DAPI) (C1006, Beyotime, China) at room temperature in the dark. After 15 min, the cells were washed with PBS, treated with a fluorescence quenching inhibitor (P0127S, Beyotime, China), and imaged under a fluorescence microscope. For quantitative analysis, blue staining indicates DAPI‐stained nuclei, while red staining indicates CD68, and green staining indicates IBA1.

### 4.8 RNA Sequencing Analysis

RNA sequencing was performed using three independent biological replicates per group. Total RNA was extracted from brain and liver tissues using TRIzol reagent according to the manufacturer's instructions. RNA purity and concentration were determined using a NanoDrop 2000 spectrophotometer (Thermo Scientific, USA), and RNA integrity was evaluated using an Agilent 2100 Bioanalyzer (Agilent Technologies, USA). Transcriptome libraries were constructed using the VAHTS Universal V5 RNA‐seq Library Prep Kit. Library preparation, sequencing, and primary bioinformatic analysis were performed by Shanghai OE Biotech Co., Ltd. (China).

Libraries were sequenced on an Illumina NovaSeq 6000 platform to generate 150‐bp paired‐end reads. Raw‐read quality was assessed using FastQC v0.11.9 and RSeQC v4.0.0. Adapter sequences and low‐quality reads were removed using fastp v0.20.1 with automatic paired‐end adapter detection. The principal filtering parameters were n_base_limit = 5, cut_window_size = 4, cut_mean_quality = 20, length_required = 75, and qualified_quality_phred = 15.

Clean reads were aligned to the NCBI *Mus musculus* GRCm39 reference genome assembly GCF_000001635.27 using HISAT2 v2.1.0 with paired‐end and reverse‐strand‐specific settings. Alignment files were converted, sorted, and indexed using SAMtools v1.9. Ribosomal RNA‐derived reads were assessed using SortMeRNA v2.1, and gene‐level raw read counts were generated using HTSeq‐count v0.11.2. FPKM values were calculated for expression‐abundance visualization, whereas the raw gene‐count matrix was used for differential expression analysis.

Principal component analysis (PCA) was performed in R v3.2.0. Differential expression analysis was conducted in R v3.5.1 using DESeq2 v1.22.2. Genes with *p* < 0.05 and |log2FC| ≥ 1 were considered differentially expressed genes (DEGs). Volcano plots, Venn diagrams, clustered expression heatmaps, and other statistical visualizations were generated using the OECloud platform (https://cloud.oebiotech.cn/task) and ggplot2 v3.4.0. GO and KEGG enrichment analyses of DEGs, as well as gene set enrichment analysis (GSEA), were performed using clusterProfiler v4.6.0.

### 4.9 Proteomic and Phosphoproteomic Analysis

#### 4.9.1 Protein Extraction and Digestion

Proteomic and phosphoproteomic analyses were performed using five independent biological replicates per group. Brain and liver tissues were pulverized under liquid nitrogen and lysed in ice‐cold lysis buffer supplemented with phosphatase inhibitors and 1 mm phenylmethylsulfonyl fluoride. Samples were homogenized at −35°C and 60 Hz for 120 s and subsequently centrifuged at 12000 rpm for 10 min at 4°C. The supernatants were collected, and protein concentrations were determined using a BCA assay kit (Thermo Fisher Scientific, USA). Protein quality was assessed by SDS‐PAGE followed by Coomassie Brilliant Blue staining. For proteomic analysis, protein extracts were adjusted to the same concentration and volume using lysis buffer. Prewashed SP3 magnetic beads were added to each protein sample, followed by acetonitrile. After incubation at room temperature for 20 min, the bead‐bound proteins were washed twice with 70% ethanol and twice with 70% acetonitrile. After removing the final wash, proteins were resuspended in 50 mM ammonium bicarbonate. Proteins were reduced with dithiothreitol at 55°C for 30 min and alkylated with chloroacetamide. Protein digestion was performed using trypsin and Lys‐C at 37°C. The resulting peptides were desalted using SOLA SPE 96‐well plates, dried under vacuum, and stored at −80°C until analysis.

For phosphoproteomic analysis, protein extracts were adjusted to the same concentration and volume, reduced with 5 mm dithiothreitol at 55°C for 30 min, and alkylated with 10 mm iodoacetamide for 15 min at room temperature in the dark. Proteins were precipitated with six volumes of acetone at −20°C for at least 4 h, centrifuged at 8000 g for 10 min at 4°C, and resuspended in 50 mM ammonium bicarbonate. Digestion was performed overnight at 37°C using Trypsin‐TPCK at an enzyme‐to‐protein mass ratio of 1:50 and terminated by acidification to approximately pH 3 with formic acid.

#### 4.9.2 Phosphopeptide Enrichment

For phosphoproteomic analysis, the digested peptides were desalted using Sep‐Pak C18 cartridges. After washing with 0.1% formic acid in water, the peptides were eluted with 50% acetonitrile containing 0.1% formic acid, lyophilized, and stored at −80°C. The lyophilized peptides were resuspended in 200 µL of binding/wash buffer. Phosphopeptide enrichment spin columns were centrifuged at 1000 g for 30 s to remove the storage buffer and equilibrated twice with 200 µL of binding/wash buffer. The peptide samples were loaded onto the columns and incubated for 30 min, with gentle mixing every 10 min. After centrifugation at 1000 g for 30 s, the flow‐through was discarded. The columns were washed three times with 200 µL of binding/wash buffer and once with 200 µL of water. The enriched phosphopeptides were eluted twice with 100 µL of elution buffer. The combined eluates were dried using a vacuum concentrator and stored at −80°C until analysis.

#### 4.9.3 LC MS Analysis

Proteomic and phosphoproteomic samples were analyzed using a Vanquish Neo ultra‐high‐performance liquid chromatography (UHPLC) system coupled to an Orbitrap Astral high‐resolution mass spectrometer (Thermo Fisher Scientific, USA). Peptides were separated on an EASY‐Spray PepMap Neo UHPLC analytical column of ES906 (150 µm × 15 cm, 2 µm, Thermo Fisher Scientific). Mobile phase: 0.1% (*v/v*) formic acid‐water (A) and 0.1% (*v/v*) formic acid‐80% acetonitrile/20% water (B).

For proteomic analysis, the elution program was as follows: 0–4 min, 4%–25% B; 4–5.8 min, 25%–35% B; 5.8–6.2 min, 35%–99% B; 6.2–6.9 min, 99%B. The flow rate was maintained at 2.5 µL·min^−1^. For phosphoproteomics analysis, the elution program was as follows: 0–0.5 min, 1%–4% B; 0.5–0.6 min, 4%–8% B; 0.6–0.9 min, 8% B; 0.9–13.9 min, 8%–22.5% B; 13.9–20.8 min, 22.5%–35% B; 20.8–21.2 min, 35%–55% B; 21.2–21.7 min, 55%–99% B; 21.7–22.6 min, 99% B; The flow rate was 1.3 µL·min^−1^ for the initial 0.5 min, decreased to 0.8 µL·min^−1^ at 0.6 min, and increased to 2.5 µL·min^−1^ from 21.2 to 22.6 min.

Both proteomic and phosphoproteomic data were acquired in data‐independent acquisition mode. Full‐scan MS spectra were acquired over an *m/z* range of 380–980 at a resolution of 240000, with an automatic gain control target of 500% and a maximum injection time of 5 ms. For MS/MS acquisition, precursor ions were fragmented by higher‐energy collisional dissociation at a normalized collision energy of 25%. The MS/MS automatic gain control target was set to 500%, with a maximum injection time of 3 ms. The RF lens level was set to 50%, and the total cycle time was 0.6 s.

#### 4.9.4 Data Processing and Database Searching

The DIA raw files were processed using Spectronaut Pulsar version v19.3. Database searching was performed against a reviewed *Mus musculus* UniProt FASTA database (taxonomy ID 10090; database file dated 7 February 2025). Trypsin/P was specified as the proteolytic enzyme, with up to two missed cleavages allowed. Carbamidomethylation of cysteine was defined as a fixed modification, whereas methionine oxidation and protein N‐terminal acetylation were defined as variable modifications. For phosphoproteomic analysis, phosphorylation of serine, threonine, and tyrosine was additionally specified as a variable modification. The precursor‐ and protein‐level q‐value cutoffs were both set to 0.01, and quantification was performed at the MS^2^ level. Proteomic and phosphoproteomic analyses were performed by Shanghai Lu‐Ming Biotech Co., Ltd., China.

#### 4.9.5 Statistical and Bioinformatic Analysis

Downstream statistical analyses were performed in R v4.2.2. Fold changes were calculated from the protein‐ and phosphosite‐abundance matrices exported from Spectronaut, and statistical significance was evaluated using Student's *t*‐test. PCA was performed using the ropls package v1.30.0. Volcano plots and functional‐enrichment bar and bubble plots were generated using ggplot2 v3.4.2. Functional enrichment analysis was performed using an in‐house computational workflow. Differential proteins and phosphoproteins were matched against locally downloaded GO, KEGG, Reactome, and WikiPathways annotation files. Enrichment significance was evaluated using a hypergeometric test, and the resulting *p‐*values were adjusted using the Benjamini‐Hochberg procedure. Kinase activity analysis was performed using KSEAapp based on kinase‐substrate relationships obtained from the PhosphoSitePlus database (http://www.phosphosite.org). Protein‐protein interaction information was retrieved from STRING v12, and interaction networks were visualized using Cytoscape v3.9.1.

### 4.10 Untargeted Metabolomics Analysis

Untargeted metabolomic analysis was performed using six independent biological replicates per group. Approximately 50 mg of brain tissue or 100 mg of liver tissue was extracted with 400 µL of pre‐cooled 75% methanol in water and homogenized. Dichloromethane (300 µL) was added, vortexed for 3 min, sonicated for 10 min, and centrifuged at 12000 rpm for 10 min at 4°C. For brain samples, 200 µL of the upper aqueous fraction (strongly polar fraction) and 150 µL of the lower organic fraction (weakly polar fraction) were collected, whereas 200 µL of each fraction was collected for liver samples. The collected fractions were dried under nitrogen and reconstituted in acetonitrile/water (4:1, *v/v*, 70 µL for brain and 120 µL for liver) for the strongly polar fraction or in acetonitrile (90 µL) for the weakly polar fraction. For serum samples, 100 µL of serum was mixed with 190 µL of methanol, followed by addition of 380 µL of dichloromethane. After vortexing and centrifugation at 12000 rpm for 10 min, the upper strongly polar and lower weakly polar fractions were separately collected and dried under nitrogen. The strongly polar fraction was reconstituted in 200 µL of acetonitrile/water (4:1, *v/v*), whereas the weakly polar fraction was reconstituted in 200 µL of acetonitrile.

For brain, liver, and serum samples, the strongly polar extracts were divided into aliquots for both untargeted and targeted metabolomic analyses, whereas the weakly polar extracts were used only for untargeted metabolomic analysis. For targeted metabolomic analysis, an internal standard solution was added to the strongly polar extracts prior to analysis.

Untargeted metabolomic analysis was performed using a Dionex Ultimate 3000 ultra‐high‐performance liquid chromatography system coupled to an LTQ Orbitrap Velos Pro mass spectrometer equipped with an ESI source (Thermo Fisher Scientific, USA). Instrument control and data acquisition were performed using Xcalibur v2.1 (Thermo Fisher Scientific). The strongly and weakly polar fractions were analyzed using hydrophilic interaction chromatography (HILIC) and reversed‐phase chromatography, respectively. For both chromatographic modes, mobile phase A consisted of 0.05% (*v/v*) formic acid in water and mobile phase B consisted of 0.05% (*v/v*) formic acid in acetonitrile.

For HILIC analysis, separation was performed on a Waters ACQUITY UPLC BEH Amide chromatographic column (2.1 mm×100 mm, 1.7 µm, Waters Corporation, USA). Elution program: 0–1 min, 97% B; 1–5 min, 97%–92% B; 5–10 min, 92%–80% B; 10–15 min, 80%–60% B; 15–16 min, 60%–50% B; 16–16.1 min, 50%–97% B; 16.1–23 min, 97% B. The flow rate was 0.2 mL·min^−1^, column temperature was 25°C. For reversed‐phase analysis, separation was performed on a Waters ACQUITY UPLC HSS T3 chromatographic column (2.1 mm × 100 mm, 1.8 µm, Waters Corporation, USA). Elution program: 0–2 min, 5%–55% B; 2–10 min, 55%–95% B; 10–15 min, 95% B; 15–15.1 min, 95%–5% B; 15.1–20 min, 5% B. The flow rate was 0.3 mL·min^−1^, and the column temperature was 35°C. The injection volume was 3 µL, and the temperature of the automatic sampler tray was set to 7°C.

Electrospray ionization (ESI) was performed in both positive and negative ion modes. Ultra‐high purity helium and nitrogen (N_2_) were used as collision and nebulizing gas, respectively. In positive ion mode, the capillary temperature was 350°C, with sheath gas and auxiliary gas flow rates of 60 arb and 10 arb, respectively. In negative ion mode, the capillary temperature was 350°C, and the sheath‐ and auxiliary‐gas flow rates were 35 and 10 arb, respectively. S‐Lens RF Level was set to 60%. Full scan spectra were acquired over an *m/z* scan range of 50–1500 at a resolution of 30000. Data‐dependent MS^2^ and MS^3^ acquisition was performed using pooled QC samples to obtain fragmentation information for metabolite annotation. Dynamic exclusion was applied with a repeat duration of 30 s, an exclusion list size of 50, and an exclusion duration of 180 s.

Raw LC‐MS data were processed using Compound Discoverer v3.3. Metabolite annotation was performed by matching accurate‐mass and MS/MS fragmentation information against online databases integrated within Compound Discoverer. The minimum peak area was set to 10000, the maximum retention‐time shift to 0.5 min, and the alignment window to 2.0 min. Retention time, *m/z*, and peak‐area information were extracted, and peak areas were normalized to the total ion current. The normalized peak areas were used to represent the relative abundance of metabolic features. QC samples were used to monitor the stability and reproducibility of the entire system. PCA was performed in R v4.1.2 with unit‐variance scaling. Orthogonal partial least squares discriminant analysis (OPLS‐DA) was performed using MetaboAnalystR v1.0.1 in R v3.5.1 after log2 transformation and mean centering. Model robustness was evaluated using seven‐fold cross‐validation and 200 permutation tests.

Differential metabolites were identified based on variable importance in projection (VIP), fold change (FC), and statistical significance. Metabolites with VIP ≥ 1.0, FC ≥ 1.20 or ≤ 0.83, and *p* < 0.05 were considered significantly altered. Two‐group comparisons were performed using Student's *t*‐test. Heatmaps were generated in R v4.2.0 using ComplexHeatmap v2.12.0 after row‐wise Z‐score normalization, with Euclidean distance and complete‐linkage clustering. KEGG pathway enrichment analysis was performed using MetaboAnalystR v1.0.1 with the built‐in *Mus musculus* KEGG pathway library. Enrichment significance was assessed using a hypergeometric test. Pathways with *p* < 0.05 were considered significantly enriched.

The multi‐omics analyses were conducted as exploratory analyses, and selected omics‐derived candidates were subsequently evaluated using qRT‐PCR, immunohistochemistry, immunofluorescence, and targeted metabolomic analysis.

### 4.11 Targeted Metabolomics Analysis

For the detection of derivatized metabolites, the tissue was accurately weighed, and an 80% methanol‐water solution was added at a ratio of weight (mg): volume (µL) = 1:10. Steel balls were added, and the mixture was homogenized using a high‐speed frozen tissue grinder. It was centrifuged at 12000 rpm for 10 min at 4°C. 20 µL of the supernatant of the tissue homogenate was taken, vacuum centrifuged to dryness, and then reconstituted with 100 µL of water. 50 µL of 175 mm 3‐NPH (75% methanol‐water solution), 50 µL of 105 mm EDC (methanol), and 50 µL of 2.5% pyridine (methanol) were added and vortex mixed. The mixture was reacted at 4°C for 30 min, then vacuum centrifuged to dryness. The dried derivatized metabolites were dissolved in methanol, and 3 volumes of water were added to make a final volume of 100 µL. For the derivatization detection, a Waters ACQUITY UPLC HSS T3 column (2.1 mm × 100 mm, 1.8 µm, Waters Corporation, USA) was used. The mobile phases were 0.03% (v/v) formic acid‐water (A) and 0.03% (v/v) formic acid‐acetonitrile (B). The elution program was: 0–1 min, 5% B; 1–15 min, 5%–73% B; 15–15.1 min, 73%–90% B; 15.1–16.5 min, 90% B; 16.5–16.6 min, 90%–5% B; 16.6–19 min, 5% B. The flow rate was 0.3 mL·min^−1^, and the column temperature was 40°C. An ESI source and multiple reaction monitoring mode were adopted. The analysis parameters of the indicators to be tested could be found in the references [15].

### 4.12 Western Blot Analysis

Total proteins from AML12 and BV2 cells were extracted using RIPA lysis buffer containing protein phosphatase inhibitor (P1275, Beijing Applygen Technology Inc., China) and protease inhibitors (P1260, Beijing Applygen Technology Inc., China). The protein samples were subjected to 10% SDS‐PAGE gel electrophoresis and then blotted onto polyvinylidene fluoride membranes. The membranes were then blocked for 2 h (5% BSA) and incubated at 4°C with PI3K p85 (4257, Cell Signaling Technology, USA), AKT (2938, Cell Signaling Technology, USA), p‐AKT (9018, Cell Signaling Technology, USA), NF‐κB p65 (9936, Cell Signaling Technology, USA), p‐p65 (3033, Cell Signaling Technology, USA), iNOS (ab178945, Abcam, UK), PPAR‐γ (16643‐1‐AP, Proteintech, China), or GAPDH (ab181602, Abcam, UK) antibody overnight. Horseradish peroxidase (HRP)‐labeled sheep anti‐rabbit IgG(7074, Cell Signaling Technology, USA) was then added, and the mixture was incubated on a shaker for 2 h. An ECL hypersensitive luminescence detection kit (P1010, Beijing Applygen Technology Inc., China) was then used for chemiluminescence detection and visualised on an imaging system (Nikon, Japan).

### 4.13 Quantitative Real‐Time PCR Analysis

RNA extraction from tissue samples was performed using the E.Z.N.A. Total RNA Kit II (OMEGA, R6934‐01). The OD260 and OD280 values were measured using a DS‐11 Denovix Ultra‐Micro Spectrophotometer (Denovix, USA), and the OD260/OD280 ratio was calculated to determine the concentration and purity of the extracted RNA. Reverse transcription was conducted using the ReverTra Ace qPCR RT Master Mix with gDNA Remover (TOYOBO, FSQ‐301) kit on a Biometra TAdvanced 96 SG gene amplification instrument (Analytik Jena, Germany), following the manufacturer's instructions. qPCR reactions were performed according to the protocol provided by the TransStart Top Green qPCR SuperMix (TransGen Biotech, AQ131‐03). Detection was carried out using an ABI QuantStudio 1 Plus Real‐time fluorescence quantitative PCR instrument (Thermo, USA), and the final data were analyzed using the 2^‐(∆∆Ct) method. The primer sequences are provided in Table S10.

### 4.14 Biochemical Markers Assay

The following assay kits were supplied by Nanjing Jiancheng Bioengineering Institute and used according to the manufacturer's protocols: the 4‐hydroxynonenal (4‐HNE) assay kit (H268‐1‐2), glucose assay kit (A154‐1‐1), total superoxide dismutase (T‐SOD) assay kit (A001‐3‐2), malondialdehyde (MDA) assay kit (A003‐1‐2), lactate assay kit (A019‐2‐1), total glutathione (T‐GSH)/oxidized glutathione (GSSG) assay kit (A061‐1‐2), and reduced glutathione (GSH) assay kit (A006‐2‐1).

### 4.15 Analytical Conditions for the Identification of HLJDD Constituents

A total of 100 mg of HLJDD powder was weighed and extracted with 500 µL of 70% methanol aqueous solution via ultrasonic extraction for 60 min. The extract was then centrifuged at 12000 rpm and 4°C for 10 min. The resulting supernatant was collected for analysis. Chromatographic separation was performed on an ACQUITY UPLC HSS T3 column (100 mm × 2.1 mm, 1.8 µm) maintained at 45°C. The mobile phase consisted of 0.1% formic acid in water (A) and acetonitrile (B), delivered at a flow rate of 0.35 mL/min. The injection volume was 2 µL. The gradient elution program was as follows: 0–3.0 min, 0% B; 3.0–18.5 min, 0–20% B; 18.5–20 min, 20%–35% B; 20–26 min, 35%–40% B; 26–35 min, 40%–95% B; 35–38 min, 95% B; 38–40 min, re‐equilibration to initial conditions. Mass spectrometric analysis was conducted using a heated electrospray ionization (HESI) source in both positive and negative ion modes. Data were acquired in data‐dependent acquisition (DDA) mode, which included a full MS scan (resolution: 60000; scan range: *m/z* 100‐1500) followed by dd‐MS^2^ scans (resolution: 15000) of the top 10 most intense ions with dynamic exclusion. The spray voltage was set to 3800 V for positive mode and −3200 V for negative mode. The capillary temperature and auxiliary gas heater temperature were 320°C and 350°C, respectively. The sheath gas and auxiliary gas flow rates were 35 arb and 8 arb, respectively. Stepped normalized collision energies (NCE) of 10, 20, and 40 were applied for fragmentation.

### 4.16 Cell Culture and Experimental Procedures

#### 4.16.1 Cell Culture and Treatment

**Cell culture**: The mouse liver parenchymal cell line Alpha Mouse Liver 12 (AML12) was obtained from the Institute of Chinese Materia Medica, China Academy of Chinese Medical Sciences (Beijing, China), for in vitro studies. AML12 cells were cultured in DMEM/F12 (CM‐0602, Procell system, China) containing 10% FBS, 1% insulin‐transferrin‐selenium (ITS), 40 ng/mL dexamethasone, 1% penicillin, and 1% streptomycin. Cells were cultured in a sterile incubator at 37°C with 5% CO_2_. Cells with good growth morphology and a confluence of 60%–70% were selected for experimental use. BV2 cells were routinely cultured in high‐glucose DMEM medium (P11965092, Gibco, USA) containing 10% FBS (10099141C, Gibco, USA), 1% penicillin, and 1% streptomycin (P6277156, Gibco, USA), under sterile conditions at 37°C with 5% CO_2_. Cells with good morphology and a confluence of 60%–70% were selected for experimentation. All reagents and procedures were performed in strict accordance with aseptic techniques, and all operations were conducted within a biosafety cabinet.

**Treatment protocol**: AML12 cells were harvested at the logarithmic growth stage (the third‐10th generations) and then transferred onto cell culture plates. When the cells reached 50%–70% confluence, they were treated with 0.5 µg/mL LPS (L2630, Sigma, Germany) for 24 h to establish the in vitro hepatocyte injury model. The HLJDD groups received LPS (0.5 µg/mL) and HLJDD (2.5, 7.5, or 25 µg/mL) simultaneously for 24 h. HLJDD at 25 µg/mL was used in the conditioned‐medium and siRNA experiments.

Small interfering RNA (siRNA) transfection: Gene silencing was performed by transfecting AML12 cells with synthetic siRNA using Lipofectamine 3000 (L3000015, Thermo Fisher Scientific, China). Cells were transfected with 30 nm of either PPARγ‐targeting siRNA (sc29456, L1922, Santa Cruz Biotechnology, USA) or a nontargeting control siRNA (siNC) (sc‐37007, Santa Cruz Biotechnology, USA) according to the manufacturer's instructions. After transfection, the cells were incubated in an incubator at 37°C with 5% CO_2_ for 6–8 h, then replaced with fresh medium containing LPS (0.5 µg/mL), either alone or together with HLJDD (25 µg/mL), and cultured for an additional 24 h. For CM‐transfer experiments, the treatment medium was subsequently removed, and CM was prepared as described above. Subsequently, cells and cell supernatants were collected for further analysis. The gene knockdown efficiency was confirmed by Western blot.

#### 4.16.2 Preparation and Transfer of Conditioned Medium

AML12 cells were treated with LPS (0.5 µg/mL), either alone or together with HLJDD (25 µg/mL), for 24 h. The treatment medium was then removed, the cells were washed with PBS (P1020, Solarbio, China), and fresh medium was added for another 24 h of culture; at this point, the supernatant of AML12 cells was collected as conditioned medium (CM). BV2 cells were exposed to the CM for 24 h, after which the cells and their culture supernatants were collected for analysis. For experiments involving PPAR‐γ knockdown, AML12 cells were transfected before LPS and HLJDD treatment.

#### 4.16.3 Cell Viability and Cytokine Measurements

Cell viability was assessed using the CCK‐8 assay (CA1210, Solarbio, China). AML12 and BV2 cells were seeded in 96‐well plates at a density of 1 × 10^4^ cells per well. After treatment, CCK‐8 reagent was added, and the cells were incubated at 37°C for 2 h. Absorbance at 450 nm was measured using a microplate reader.

For cytokine measurements, AML12 cells were treated with LPS (0.5 µg/mL) alone or in combination with HLJDD (25 µg/mL). TNF‑α, IL‑1β, and IL‐6 concentrations in AML12 culture supernatants and in BV2 culture supernatants collected after CM exposure were measured using mouse ELISA kits according to the manufacturers’ instructions. The ELISA kits were purchased from Elabscience Biotechnology Co., Ltd. (China): TNF‐α (E‐EL‐M3063), IL‐6 (E‐EL‐M0044), and IL‐1β (E‐EL‐M0037).

### 4.17 Statistical Analysis

No formal a priori power calculation was performed. Sample sizes were determined based on preliminary observations, commonly used sample sizes in 5xFAD studies, anticipated variability, analytical feasibility, planned allocation across assays, and animal‐welfare considerations. Statistical analyses of behavioral, biochemical, histological, qRT‐PCR, and cell‐based validation data were performed using IBM SPSS Statistics and GraphPad Prism. For animal experiments, each animal was considered an independent biological replicate, and technical replicates were averaged before analysis. For cell‐based Western blot and ELISA analyses, independent experiments were treated as biological replicates. Unless otherwise indicated, data are presented as mean ± SEM. Normality and homogeneity of variance were assessed using the Shapiro‐Wilk and Levene's tests, respectively.

For comparisons among more than two independent groups at a single time point, one‐way ANOVA with Tukey's post hoc test was used for normally distributed data with equal variances, Welch's ANOVA with Games‐Howell test for normally distributed data with unequal variances, and Kruskal‐Wallis test with Dunn's multiple‐comparisons test for non‐normally distributed data. For two‐group comparisons, unpaired two‐tailed Student's *t*‐test, Welch's *t*‐test, or Mann–Whitney U test was used as appropriate. Morris water maze escape latency was analyzed using two‐way repeated‐measures ANOVA, with Greenhouse‐Geisser correction when necessary, followed by Šídák's multiple‐comparisons test. All applicable tests were two‐sided. Statistical significance was defined as *p* < 0.05, using multiplicity‐adjusted *p*‐values for post hoc comparisons. Omics‐specific statistical methods and false discovery rate control are described in the corresponding Methods subsections.

## Author Contributions

**Wenya Gao**: Writing – Original Draft, Writing – Review & Editing, Methodology, Software, Validation, Formal analysis, Investigation, Visualization. **Jing Gao**: Methodology, Investigation, Validation, Formal analysis. **Xiaohui Su**: Methodology, Investigation, Validation, Formal analysis. **Xiangying Kong**: Methodology. **Xiaolu Wei**: Methodology and Formal analysis. **Tunan Ding**: Methodology and Formal analysis. **Tiantian Guo**: Methodology and Formal analysis. **Shuangrong Gao**: Supervision. **Yanyan Zhou**: Investigation. **Junyi Zhou**: Methodology, Investigation, Validation, Writing – review & editing. **Haiyu Zhao**: Project administration, Resources and Funding acquisition. All authors reviewed the manuscript.

## Funding

The work was aided financially by the Scientific and Technological Innovation Project of China Academy of Chinese Medical Sciences (CI2023C020YL, CI2023E001TS09, CI2026A04509); the National Natural Science Foundation of China (grant numbers 81974523).

## Conflicts of Interest

The authors declare no conflicts of interest.

## Supporting information




**Supporting File**: advs78442‐sup‐0001‐SuppMat.docx.

## Data Availability

The data that support the findings of this study are openly available in the National Genomics Data Center (NGDC), China National Center, at https://ngdc.cncb.ac.cn/, reference numbers PRJCA067098; OMIX019542; OMIX018709; PRJCA069400; OMIX018963.
