# Supplementary material for: Cross‐Organ Multi‐Omics Profiling of Systemic Alterations in 5xFAD Mice and Changes Associated with Huanglian Jiedu Decoction Treatment
Source: Adv Sci (Weinh). 2026 Oct 31:e78442. Online ahead of print. doi: 10.1002/advs.78442 (PMC13723713; doi:10.1002/advs.78442)
Supplement: Supplementary file 1 [file ADVS-9999-e78442-s001.docx]

# ***Supporting Information***

# Cross-Organ Multi-Omics Profiling of Systemic Alterations in 5xFAD Mice and Changes Associated with Huanglian Jiedu Decoction Treatment

***Authors:*** *Wenya Gao^1,2#^, Jing Gao^1#^, Xiaohui Su^1#^, Xiangying Kong^1^, Xiaolu Wei^1^, Tunan Ding^2^, Tiantian Guo^2^, Shuangrong Gao^1^, Yanyan Zhou^1^, Junyi Zhou^1*^, Haiyu Zhao^1*^*

***Affiliations:***

1 Institute of Chinese Materia Medica, China Academy of Chinese Medical Sciences, Beijing 100700, China.

2 Beijing Academy of Traditional Chinese Medicine, Beijing University of Chinese Medicine, Beijing 100029, China.

Author E-mail address:

Wenya Gao, [wenyagao@bucm.edu.cn;](mailto:wenyagao@bucm.edu.cn;) Jing Gao, [gj9805@126.com;](mailto:gj9805@126.com;) Xiaohui Su, [xhsu@icmm.ac.cn;](mailto:xhsu@icmm.ac.cn;) Xiangying Kong, [xykong@icmm.ac.cn;](mailto:xykong@icmm.ac.cn;) Xiaolu Wei, [xlwei@icmm.ac.cn;](mailto:xlwei@icmm.ac.cn;) Tunan Ding, [20220123072@bucm.edu.cn;](mailto:20220123072@bucm.edu.cn,) Tiantian Guo, [slhsxguotiantian@126.com;](mailto:slhsxguotiantian@126.com;) Shuangrong Gao, [srgao@icmm.ac.cn;](mailto:srgao@icmm.ac.cn;) Yanyan Zhou, [yyzhou@icmm.ac.cn;](mailto:yyzhou@icmm.ac.cn;) Junyi Zhou, [zzjjyyy@163.com;](mailto:zzjjyyy@163.com;) Haiyu Zhao, [hyzhao@icmm.ac.cn](mailto:hyzhao@icmm.ac.cn)

Correspondence: Haiyu Zhao and Junyi Zhou

Institute of Chinese Materia Medica, China Academy of Chinese Medical Sciences, No.16, Nan St., Dongcheng Dist., Beijing 100700, China.

^#^Wenya Gao, Jing Gao, and Xiaohui Su contributed equally to this work and shared the first author.

**Supplementary Table**

**Table S1.** Differential metabolites in brain tissues of 5xFAD mice (WT group vs AD group).

**Table S2.** Differential metabolites in liver tissues of 5xFAD mice (WT group vs AD group).

**Table S3.** Tentative identification of compounds in HLJDD.

**Table S4.** Putatively identified prototype compounds and metabolites of HLJDD in mouse plasma, liver, and brain samples collected 2 h after a single oral administration.

**Table S5.** Comparisons of body weight between groups at each time point.

**Table S6.** The visceral-body ratio of mice in each group after drug administration.

**Table S7.** Differential metabolites in brain tissues of 5xFAD mice (HLJDD group vs AD group).

**Table S8.** Differential metabolites in liver tissues of 5xFAD mice (HLJDD group vs AD group).

**Table S9.** Concentration-response standard curve of neurotransmitters and tricarboxylic acid cycle metabolite.

**Table S10.** List of primer sequences for the target gene.

**Table S11.** Animal allocation and attrition.

**Table S12.** Assay-specific biological sample sizes.

**Table S13.** Randomization, blinding, batch allocation, and sample-size rationale.

**Supplementary Figure**

**Figure S1. Integrated proteomic and phosphoproteomic analyses of liver tissues from WT and AD mice.**

(A) Summary of proteins and peptides identified by DIA-based proteomics in liver tissue. (B) Venn diagram showing the overlap between identified total proteins and phosphoproteins. (C) PCA of DIA proteomic profiles in liver tissues from WT and AD mice. (D, E) Volcano plot (D) and clustering heatmap (E) of DEPs in liver tissues between WT and AD mice. (F) PCA of phosphoproteomic profiles in liver samples from WT and AD mice. (G, H) Volcano plot (G) and clustering heatmap (H) of DPPs in liver tissues between WT and AD mice. (I, J) KEGG pathway enrichment (I) and GO term enrichment (J) analyses of DEPs identified by DIA-based proteomics. (K) PPI network and hub protein ranking of DEPs. (L) Venn diagram of overlapping DEGs and DEPs in liver tissue. (M) Quadrant scatter plot comparing fold changes at the RNA and protein levels in liver tissues. (N) PPI network and multi-omics clustering heatmap of overlapping DEGs and DEPs. (O, P) GO term enrichment (O) and KEGG pathway enrichment (P) analyses of DPPs identified by phosphoproteomics. (Q) Bubble plot of the top 20 kinase-substrate activity in WT and AD mice. Proteomic and phosphoproteomic analyses in A-K and O-Q were performed using *n* = 5 biological replicates per group. Integrated transcriptomic-proteomic analyses in L-N were based on transcriptomic data with *n* = 3 biological replicates per group and proteomic data with *n* = 5 biological replicates per group.

**Figure S2. Chromatographic profiles and multivariate statistical analysis of liver and serum samples in WT and AD model mice**

(A) Overlay of BPC from QC samples of 5xFAD mice liver samples under different detection conditions. (B) Overlay of BPC from QC samples of 5xFAD mice serum samples under different detection conditions. (C) The OPLS-DA plots of serum samples between WT and AD groups. Untargeted metabolomic analyses were performed using *n* = 6 biological replicates per group.

**Figure S3. UHPLC-HRMS chromatographic profiles of brain, liver, and plasma samples after single-dose oral administration of HLJDD in mice.**

Vertically offset base peak chromatograms (BPCs) of brain tissue (A), liver tissue (B), and plasma (C) samples acquired in positive and negative ionization modes. Male C57BL/6 mice were randomly assigned to the vehicle control group (V), clinically equivalent-dose HLJDD group (CE, 1160 mg/kg), or HLJDD high-dose group (H, 116 mg/kg) (*n* = 6 per group). After overnight fasting, mice received a single oral gavage of vehicle or HLJDD, and samples were collected 2 h after administration. Blood was collected before perfusion for plasma preparation, whereas liver and brain tissues were harvested after systemic perfusion with pre-chilled PBS to minimize residual blood contamination. V1-V6, CE1-CE6, and H1-H6 denote individual biological samples from the corresponding groups.

**Figure S4. Integrated proteomic and phosphoproteomic profiling of liver tissue samples between HLJDD and AD groups.**

(A) PCA of DIA proteomic profiles in liver tissue samples between HLJDD and AD groups. Volcano plot (B) and clustering heatmap (C) of DEPs in liver tissue samples between HLJDD and AD groups. (D) PCA of phosphoproteomic profiles in liver tissue samples between HLJDD and AD groups. Volcano plot (E) and clustering heatmap (F) of DPPs in liver tissue samples between HLJDD and AD groups. KEGG pathway (G) and GO enrichment (H) of DEPs from DIA proteomics in liver tissue samples between HLJDD and AD groups. (I) PPI network and hub protein ranking of DEPs from DIA proteomics in liver tissue samples between HLJDD and AD groups. KEGG pathway (J) and GO enrichment (K) of DPPs from phosphoproteomics in liver tissue samples between HLJDD and AD groups. (L) PPI network and hub protein ranking of DPPs from phosphoproteomics in liver tissue samples between HLJDD and AD groups. (M) KEGG pathway enrichment analysis of integrated proteomic and phosphoproteomic data in liver tissue samples between HLJDD and AD groups. (N) Bar plot of kinase activity enrichment in liver tissue samples between HLJDD and AD groups. (O) GSEA bar plot of integrated transcriptomic and proteomic analysis in liver tissue samples between HLJDD and AD groups. Proteomic and phosphoproteomic analyses were performed using *n* = 5 biological replicates per group.

**Figure S5. Metabolomic profiling and pathway enrichment analysis of brain, liver, and serum samples following HLJDD treatment in 5xFAD mice.**

(A) PCA score plots of brain metabolomic profiles from WT, AD, and HLJDD-treated mice. (B) Clustering heatmap of differential metabolites in brain tissues from 5×FAD mice under different analytical conditions. (C) KEGG pathway enrichment analysis of differential metabolites in brain tissues between HLJDD-treated and AD mice. (D) OPLS-DA score plots of liver metabolomic profiles between HLJDD-treated and AD mice. (E) KEGG pathway enrichment analysis of differential metabolites in liver tissues between HLJDD-treated and AD mice. (F) OPLS-DA score plot of serum metabolomic profiles between HLJDD-treated and AD mice. (G) KEGG pathway enrichment analysis of differential metabolites in serum samples between HLJDD-treated and AD groups (G). Untargeted metabolomic analyses in A-G were performed using *n* = 6 biological replicates per group.

**Figure S6. Multivariate statistical analysis of liver and serum metabolomic profiles in 5xFAD mice following HLJDD treatment.**

(A) The PCA score plots of liver tissue samples from WT/AD/HLJDD. (B) The PCA score plots of serum samples from WT/AD/HLJDD. (C) The cluster heatmap of 5xFAD mice serum samples under different detection conditions. Untargeted metabolomic analyses were performed using *n* = 6 biological replicates per group.

**Figure S7. Effect of HLJDD on inflammatory and oxidative stress markers in 5xFAD mice.**

(A) GSEA enrichment plot of positive regulation of tumor necrosis factor production. (B) Heatmap showing the expression patterns of genes involved in positive regulation of tumor necrosis factor production. (C) qRT-PCR analysis of pro-inflammatory cytokines (*Tnfa*, *Ifnγ*, *Il12a*, *Il1β*) in brain tissues (*n* = 3). (D) qRT-PCR analysis of *Il6* expression in brain tissues (*n* = 3). (E) qRT-PCR analysis of *Il6* mRNA expression in liver tissues (*n* = 3). (F) Biochemical quantification of oxidative stress markers, including nitric oxide (NO), glutathione (GSH), superoxide dismutase (SOD), and lipid peroxidation product 4-hydroxynonenal (4HNE) in brain, liver, and serum samples (*n* = 6). (G) Heatmap showing the expression patterns of genes involved in nitrogen metabolism. Quantitative data are presented as mean ± SEM.

**Figure S8. Multi-omics integration reveals the mechanism of action after HLJDD intervention.**

(A) Nine-quadrant analysis of AD vs WT and AD vs HLJDD in brain tissue. (B) Nine-quadrant analysis of AD vs WT and AD vs HLJDD in liver tissue. (C) Kmeans plot of multi-omics correlation between transcriptome and metabolome in brain tissue. (D) Kmeans plot of multi-omics correlation between transcriptome and metabolome in liver tissue. (E) Relative mRNA expressions of *Hnf4a*, *Pck1*, *Fbp1*, *Atp2a1*, *G6pc*, *Csf3r* in brain tissue (*n* = 3). All the results are expressed as the mean ± SEM.

**Figure S9. Targeted metabolomic profiling of amino acids and nitrogen metabolism-related metabolites in 5xFAD mice.**

Targeted metabolomic analysis of amino acids and nitrogen metabolism-related metabolites in the (A) brain, (B) liver, and (C) serum of WT, AD, and HLJDD groups. Data are presented as the mean ± SEM (*n* = 6 biologically independent mice per group). Exact P values for the indicated pairwise comparisons are shown above the brackets.

**Figure S10. Targeted metabolomic profiling of neurotransmitter- and nucleotide-related metabolites in 5xFAD mice.**

(A) Heatmap visualization of differential metabolites in brain, liver, and serum among WT, AD, and HLJDD groups.Targeted metabolomic analysis of choline, acetylcholine, catecholamines, γ-aminobutyric acid, histamine, adenosine, uridine, and related metabolites in the (B) brain, (C) liver, and (D) serum of WT, AD, and HLJDD groups. Data are presented as the mean ± SEM (*n* = 6 biologically independent mice per group). Exact P values for the indicated pairwise comparisons are shown above the brackets.

**Figure S11. Targeted metabolomic profiling of tryptophan metabolism in 5xFAD mice.**

Targeted metabolomic analysis of tryptophan and its downstream metabolites associated with the serotonin, indole, and kynurenine pathways in the (A) brain, (B) liver, and (C) serum of WT, AD, and HLJDD groups. Data are presented as the mean ± SEM (*n* = 6 biologically independent mice per group). Exact *P* values for the indicated pairwise comparisons are shown above the brackets. 5-HT, 5-hydroxytryptamine.

**Figure S12. Integrative analysis of brain–liver molecular signatures and candidate communication networks in response to HLJDD treatment.**

(A) GSVA effect estimates for AD versus WT and HLJDD versus AD across brain and liver transcriptomic and proteomic datasets. Red and blue indicate positive and negative effects, respectively. Point size represents -log10(P). Asterisks indicate FDR < 0.05, filled circles indicate nominal *P* < 0.05 without FDR significance, and open circles indicate nominal *P* ≥ 0.05. (B) Restoration evidence for the top prespecified metabolic and inflammatory pathways. Red indicates directional reversal, whereas blue indicates continuation of the disease-associated direction. Scores were truncated at ± 2 for visualization. Point size represents treatment -log10(P). (C) Integrated ranking of liver-tissue ligand candidates based on liver RNA/protein reversal evidence, extracellular or secreted-protein annotation, brain receptor support, module-specific NicheNet activity, and predicted target regulatory potential. Point size indicates the number of detected brain receptors assigned to the selected pathway modules. colors indicate candidate evidence tiers. (D) Compact liver candidate ligand-brain receptor/target network. Database-supported ligand-receptor interactions and NicheNet-predicted ligand-target relationships are shown for selected liver-derived candidate ligands with lower imputation sensitivity. Node colors indicate liver ligands, brain receptors, and brain response targets, whereas edge colors represent different interaction evidence or response modules. (E) Serum metabolite log2 fold changes in WT versus AD and HLJDD versus AD comparisons. Identical signs in these two AD-denominator contrasts indicate a treatment-associated change in the direction of the WT value. Dark red points identify core biological candidates, and crosses mark potential exogenous or contaminant features excluded from the core network. Point size represents the maximum VIP score across comparisons. (F) Candidate liver-serum-brain network linking directionally reversed serum metabolites to selected liver and brain pathway signatures through biochemical-class mapping. Edge colors indicate pathway modules.

**Figure S13 Assessment of cell viability, inflammatory markers, and signaling-related proteins in AML12 and BV2 cells under HLJDD treatment and hepatocyte PPAR-γ knockdown conditions.**

(A) AML12 cell viability assessed using the CCK-8 assay (*n* = 6). (B) Quantification of PPAR-γ, PI3K p85 and iNOS protein expression normalized to GAPDH, and the p-AKT/AKT and p-p65/p65 ratios in AML12 cells following simultaneous treatment with LPS and the indicated concentrations of HLJDD (*n* = 3). (C) BV2 cell viability assessed using the CCK-8 assay following exposure to AML12-derived conditioned medium (CM, *n* = 6). (D) Fluorescence intensity of Iba1 and CD68 in BV2 cell (*n* = 5). (E) Quantification of PPAR-γ, PI3K p85, and iNOS protein expression normalized to GAPDH, and the p-AKT/AKT and p-p65/p65 ratios in BV2 cells exposed to AML12-derived CM (*n* = 3). (F) Quantification of PPAR-γ, PI3K p85, and iNOS protein expression normalized to GAPDH, and the p-AKT/AKT and p-p65/p65 ratios in AML12 cells subjected to the indicated siRNA and treatment conditions (*n* = 3). (G) Quantification of PPAR-γ, PI3K p85, and iNOS protein expression normalized to GAPDH, and the p-AKT/AKT and p-p65/p65 ratios in BV2 cells exposed to CM from AML12 cells subjected to the indicated siRNA and treatment conditions. HLJDD was used at 25 μg/mL in the CM-transfer and siRNA experiments (*n* = 3). (H) Levels of TCA cycle-related metabolites and glucose in BV2 cells exposed to conditioned media derived from AML12 cells following PPAR-γ knockdown and the indicated treatments, including aconitiate, citrate, fumarate, glucose, isocitrate, malate, oxaloacetate, and succinate (*n* = 3). Data were presented as means ± SEM.

**Figure S14. Uncropped Western blot images of HLJDD-treated AML12 hepatocytes corresponding to Figure 11B.**

**Figure S15. Uncropped Western blot images of BV2 cells exposed to AML12-derived conditioned medium corresponding to Figure 11E.**

**Figure S16. Uncropped Western blot images of AML12 hepatocytes following PPAR-γ knockdown and HLJDD treatment corresponding to Figure 11F.**

**Figure S17. Uncropped Western blot images of BV2 cells exposed to conditioned medium from PPARγ-silenced AML12 hepatocytes corresponding to Figure 11H.**

**Figure S1. Integrated proteomic and phosphoproteomic analyses of liver tissues from WT and AD mice.**

(A) Summary of proteins and peptides identified by DIA-based proteomics in liver tissue. (B) Venn diagram showing the overlap between identified total proteins and phosphoproteins. (C) PCA of DIA proteomic profiles in liver tissues from WT and AD mice. (D, E) Volcano plot (D) and clustering heatmap (E) of DEPs in liver tissues between WT and AD mice. (F) PCA of phosphoproteomic profiles in liver samples from WT and AD mice. (G, H) Volcano plot (G) and clustering heatmap (H) of DPPs in liver tissues between WT and AD mice. (I, J) KEGG pathway enrichment (I) and GO term enrichment (J) analyses of DEPs identified by DIA-based proteomics. (K) PPI network and hub protein ranking of DEPs. (L) Venn diagram of overlapping DEGs and DEPs in liver tissue. (M) Quadrant scatter plot comparing fold changes at the RNA and protein levels in liver tissues. (N) PPI network and multi-omics clustering heatmap of overlapping DEGs and DEPs. (O, P) GO term enrichment (O) and KEGG pathway enrichment (P) analyses of DPPs identified by phosphoproteomics. (Q) Bubble plot of the top 20 kinase-substrate activity in WT and AD mice. Proteomic and phosphoproteomic analyses in A-K and O-Q were performed using *n* = 5 biological replicates per group. Integrated transcriptomic-proteomic analyses in L-N were based on transcriptomic data with *n* = 3 biological replicates per group and proteomic data with *n* = 5 biological replicates per group.

**Figure S2. Chromatographic profiles and multivariate statistical analysis of liver and serum samples in WT and AD model mice**

(A) Overlay of BPC from QC samples of 5xFAD mice liver samples under different detection conditions. (B) Overlay of BPC from QC samples of 5xFAD mice serum samples under different detection conditions. (C) The OPLS-DA plots of serum samples between WT and AD groups. Untargeted metabolomic analyses were performed using *n* = 6 biological replicates per group.

**Figure S3. UHPLC-HRMS chromatographic profiles of brain, liver, and plasma samples after single-dose oral administration of HLJDD in mice.**

Vertically offset base peak chromatograms (BPCs) of brain tissue (A), liver tissue (B), and plasma (C) samples acquired in positive and negative ionization modes. Male C57BL/6 mice were randomly assigned to the vehicle control group (V), clinically equivalent-dose HLJDD group (CE, 1160 mg/kg), or HLJDD high-dose group (H, 116 mg/kg) (*n* = 6 per group). After overnight fasting, mice received a single oral gavage of vehicle or HLJDD, and samples were collected 2 h after administration. Blood was collected before perfusion for plasma preparation, whereas liver and brain tissues were harvested after systemic perfusion with pre-chilled PBS to minimize residual blood contamination. V1-V6, CE1-CE6, and H1-H6 denote individual biological samples from the corresponding groups.

**Figure S4. Integrated proteomic and phosphoproteomic profiling of liver tissue samples between HLJDD and AD groups.**

(A) PCA of DIA proteomic profiles in liver tissue samples between HLJDD and AD groups. Volcano plot (B) and clustering heatmap (C) of DEPs in liver tissue samples between HLJDD and AD groups. (D) PCA of phosphoproteomic profiles in liver tissue samples between HLJDD and AD groups. Volcano plot (E) and clustering heatmap (F) of DPPs in liver tissue samples between HLJDD and AD groups. KEGG pathway (G) and GO enrichment (H) of DEPs from DIA proteomics in liver tissue samples between HLJDD and AD groups. (I) PPI network and hub protein ranking of DEPs from DIA proteomics in liver tissue samples between HLJDD and AD groups. KEGG pathway (J) and GO enrichment (K) of DPPs from phosphoproteomics in liver tissue samples between HLJDD and AD groups. (L) PPI network and hub protein ranking of DPPs from phosphoproteomics in liver tissue samples between HLJDD and AD groups. (M) KEGG pathway enrichment analysis of integrated proteomic and phosphoproteomic data in liver tissue samples between HLJDD and AD groups. (N) Bar plot of kinase activity enrichment in liver tissue samples between HLJDD and AD groups. (O) GSEA bar plot of integrated transcriptomic and proteomic analysis in liver tissue samples between HLJDD and AD groups. Proteomic and phosphoproteomic analyses were performed using *n* = 5 biological replicates per group.

**Figure S5. Metabolomic profiling and pathway enrichment analysis of brain, liver, and serum samples following HLJDD treatment in 5xFAD mice.**

(A) PCA score plots of brain metabolomic profiles from WT, AD, and HLJDD-treated mice. (B) Clustering heatmap of differential metabolites in brain tissues from 5×FAD mice under different analytical conditions. (C) KEGG pathway enrichment analysis of differential metabolites in brain tissues between HLJDD-treated and AD mice. (D) OPLS-DA score plots of liver metabolomic profiles between HLJDD-treated and AD mice. (E) KEGG pathway enrichment analysis of differential metabolites in liver tissues between HLJDD-treated and AD mice. (F) OPLS-DA score plot of serum metabolomic profiles between HLJDD-treated and AD mice. (G) KEGG pathway enrichment analysis of differential metabolites in serum samples between HLJDD-treated and AD groups (G). Untargeted metabolomic analyses in A-G were performed using *n* = 6 biological replicates per group.

**Figure S6. Multivariate statistical analysis of liver and serum metabolomic profiles in 5xFAD mice following HLJDD treatment.**

(A) The PCA score plots of liver tissue samples from WT/AD/HLJDD. (B) The PCA score plots of serum samples from WT/AD/HLJDD. (C) The cluster heatmap of 5xFAD mice serum samples under different detection conditions. Untargeted metabolomic analyses were performed using *n* = 6 biological replicates per group.

**Figure S7. Effect of HLJDD on inflammatory and oxidative stress markers in 5xFAD mice.**

(A) GSEA enrichment plot of positive regulation of tumor necrosis factor production. (B) Heatmap showing the expression patterns of genes involved in positive regulation of tumor necrosis factor production. (C) qRT-PCR analysis of pro-inflammatory cytokines (*Tnfa*, *Ifnγ*, *Il12a*, *Il1β*) in brain tissues (*n* = 3). (D) qRT-PCR analysis of *Il6* expression in brain tissues (*n* = 3). (E) qRT-PCR analysis of *Il6* mRNA expression in liver tissues (*n* = 3). (F) Biochemical quantification of oxidative stress markers, including nitric oxide (NO), glutathione (GSH), superoxide dismutase (SOD), and lipid peroxidation product 4-hydroxynonenal (4HNE) in brain, liver, and serum samples (*n* = 6). (G) Heatmap showing the expression patterns of genes involved in nitrogen metabolism. Quantitative data are presented as mean ± SEM.

**Figure S8. Multi-omics integration reveals the mechanism of action after HLJDD intervention.**

(A) Nine-quadrant analysis of AD vs WT and AD vs HLJDD in brain tissue. (B) Nine-quadrant analysis of AD vs WT and AD vs HLJDD in liver tissue. (C) Kmeans plot of multi-omics correlation between transcriptome and metabolome in brain tissue. (D) Kmeans plot of multi-omics correlation between transcriptome and metabolome in liver tissue. (E) Relative mRNA expressions of *Hnf4a*, *Pck1*, *Fbp1, Atp2a1, G6pc, Csf3r* in brain tissue (*n* = 3). All the results are expressed as the mean ± SEM.

**Figure S9. Targeted metabolomic profiling of amino acids and nitrogen metabolism-related metabolites in 5xFAD mice.**

Targeted metabolomic analysis of amino acids and nitrogen metabolism-related metabolites in the (A) brain, (B) liver, and (C) serum of WT, AD, and HLJDD groups. Data are presented as the mean ± SEM (*n* = 6 biologically independent mice per group). Exact *P* values for the indicated pairwise comparisons are shown above the brackets.

**Figure S10. Targeted metabolomic profiling of neurotransmitter- and nucleotide-related metabolites in 5xFAD mice.**

(A) Heatmap visualization of differential metabolites in brain, liver, and serum among WT, AD, and HLJDD groups.Targeted metabolomic analysis of choline, acetylcholine, catecholamines, γ-aminobutyric acid, histamine, adenosine, uridine, and related metabolites in the (B) brain, (C) liver, and (D) serum of WT, AD, and HLJDD groups. Data are presented as the mean ± SEM (*n* = 6 biologically independent mice per group). Exact P values for the indicated pairwise comparisons are shown above the brackets.

**Figure S11. Targeted metabolomic profiling of tryptophan metabolism in 5xFAD mice.**

Targeted metabolomic analysis of tryptophan and its downstream metabolites associated with the serotonin, indole, and kynurenine pathways in the (A) brain, (B) liver, and (C) serum of WT, AD, and HLJDD groups. Data are presented as the mean ± SEM (*n* = 6 biologically independent mice per group). Exact *P* values for the indicated pairwise comparisons are shown above the brackets. 5-HT, 5-hydroxytryptamine.

**Figure S12. Integrative analysis of brain–liver molecular signatures and candidate communication networks in response to HLJDD treatment.**

(A) GSVA effect estimates for AD versus WT and HLJDD versus AD across brain and liver transcriptomic and proteomic datasets. Red and blue indicate positive and negative effects, respectively. Point size represents −log10(P). Asterisks indicate FDR < 0.05, filled circles indicate nominal P < 0.05 without FDR significance, and open circles indicate nominal P ≥ 0.05. (B) Restoration evidence for the top prespecified metabolic and inflammatory pathways. Red indicates directional reversal, whereas blue indicates continuation of the disease-associated direction. Scores were truncated at ± 2 for visualization. Point size represents treatment −log10(P). (C) Integrated ranking of liver-tissue ligand candidates based on liver RNA/protein reversal evidence, extracellular or secreted-protein annotation, brain receptor support, module-specific NicheNet activity, and predicted target regulatory potential. Point size indicates the number of detected brain receptors assigned to the selected pathway modules. colors indicate candidate evidence tiers. (D) Compact liver candidate ligand-brain receptor/target network. Database-supported ligand-receptor interactions and NicheNet-predicted ligand-target relationships are shown for selected liver-derived candidate ligands with lower imputation sensitivity. Node colors indicate liver ligands, brain receptors, and brain response targets, whereas edge colors represent different interaction evidence or response modules. (E) Serum metabolite log2 fold changes in WT versus AD and HLJDD versus AD comparisons. Identical signs in these two AD-denominator contrasts indicate a treatment-associated change in the direction of the WT value. Dark red points identify core biological candidates, and crosses mark potential exogenous or contaminant features excluded from the core network. Point size represents the maximum VIP score across comparisons. (F) Candidate liver-serum-brain network linking directionally reversed serum metabolites to selected liver and brain pathway signatures through biochemical-class mapping. Edge colors indicate pathway modules.

**Figure S13 Assessment of cell viability, inflammatory markers, and signaling-related proteins in AML12 and BV2 cells under HLJDD treatment and hepatocyte PPAR-γ knockdown conditions.**

(A) AML12 cell viability assessed using the CCK-8 assay (*n* = 6). (B) Quantification of PPAR-γ, PI3K p85 and iNOS protein expression normalized to GAPDH, and the p-AKT/AKT and p-p65/p65 ratios in AML12 cells following simultaneous treatment with LPS and the indicated concentrations of HLJDD (*n* = 3). (C) BV2 cell viability assessed using the CCK-8 assay following exposure to AML12-derived conditioned medium (CM; *n* = 6). (D) Fluorescence intensity of Iba1 and CD68 in BV2 cell (*n* = 5). (E) Quantification of PPAR-γ, PI3K p85, and iNOS protein expression normalized to GAPDH, and the p-AKT/AKT and p-p65/p65 ratios in BV2 cells exposed to AML12-derived CM (*n* = 3). (F) Quantification of PPAR-γ, PI3K p85, and iNOS protein expression normalized to GAPDH, and the p-AKT/AKT and p-p65/p65 ratios in AML12 cells subjected to the indicated siRNA and treatment conditions (*n* = 3). (G) Quantification of PPAR-γ, PI3K p85, and iNOS protein expression normalized to GAPDH, and the p-AKT/AKT and p-p65/p65 ratios in BV2 cells exposed to CM from AML12 cells subjected to the indicated siRNA and treatment conditions. HLJDD was used at 25 μg/mL in the CM-transfer and siRNA experiments (*n* = 3). (H) Levels of TCA cycle-related metabolites and glucose in BV2 cells exposed to conditioned media derived from AML12 cells following PPAR-γ knockdown and the indicated treatments, including aconitiate, citrate, fumarate, glucose, isocitrate, malate, oxaloacetate, and succinate (*n* = 3). Data were presented as means ± SEM.

**Figure S14. Uncropped Western blot images of HLJDD-treated AML12 hepatocytes corresponding to Figure 11B.**

**Figure S15. Uncropped Western blot images of BV2 cells exposed to AML12-derived conditioned medium corresponding to Figure 11E.**

**Figure S16. Uncropped Western blot images of AML12 hepatocytes following PPAR-γ knockdown and HLJDD treatment corresponding to Figure 11F.**

**Figure S17. Uncropped Western blot images of BV2 cells exposed to conditioned medium from PPAR-γ-silenced AML12 hepatocytes corresponding to Figure 11H.**

**Table S1. Differential metabolites in brain tissues of 5xFAD mice (WT group vs AD group).**

| Identification | Formula | Reference ion | Measured [*m/z*] | Calculated [*m/z*] | Delta Mass [ppm] | RT [min] | Mode | Log2 FC: (WT) / (AD) | P-value: (WT) / (AD) | VIP: (WT) / (AD) |
| --- | --- | --- | --- | --- | --- | --- | --- | --- | --- | --- |
| Oleamide | C_18_H_35_NO | [M+H]^+^ | 282.2795 | 281.2722 | 1.15 | 1.687 | HILIC | 4.06 | 0.011586 | 1.24633 |
| Epinephrine | C_9_H_13_NO_3_ | [M+H]^+^ | 184.0965 | 183.0892 | -1.94 | 2.234 | HILIC | 1.21 | 0.000134 | 1.26408 |
| Dimethyl(2-tridecanyl)amine oxide | C_15_H_33_NO | [M+H]^+^ | 244.2631 | 243.2558 | -1.68 | 2.693 | HILIC | -0.58 | 0.017828 | 1.27238 |
| N-Acetyl-L-methionine | C_7_H_13_NO_3_S | [M-H]^-^ | 190.0533 | 191.0606 | -5.30 | 3.054 | HILIC | 1.96 | 0.002467 | 1.25195 |
| 4-Trimethylammoniobutanoic acid | C_7_H_15_NO_2_ | [M+H]^+^ | 146.1174 | 145.1101 | -1.35 | 3.115 | HILIC | 1.39 | 1.36E-08 | 1.75496 |
| Malonic acid | C_3_H_4_O_4_ | [M-H]^-^ | 103.0033 | 104.0106 | -3.40 | 3.254 | HILIC | -1.16 | 0.000253 | 1.09939 |
| 2-Amino-1,3,4-octadecanetriol | C_18_H_39_NO_3_ | [M+H]^+^ | 318.2998 | 317.2925 | -1.57 | 3.404 | HILIC | -0.86 | 5.25E-08 | 1.56062 |
| gamma-Thiomethyl glutamate | C_6_H_11_NO_4_S | [M-H]^-^ | 192.0326 | 193.0399 | -5.24 | 3.698 | HILIC | 2.12 | 0.000166 | 1.26833 |
| Butane-2,3-dione | C_4_H_6_O_2_ | [M+H]^+^ | 87.0439 | 86.0366 | -1.90 | 3.869 | HILIC | 2.21 | 0.002612 | 1.44061 |
| Dehydroascorbic acid | C_6_H_6_O_6_ | [M-H]^-^ | 173.0084 | 174.0157 | -4.40 | 4.154 | HILIC | -0.93 | 0.000453 | 1.51151 |
| D-Glucuronic acid | C_6_H_10_O_7_ | [M-H]^-^ | 193.0344 | 194.0417 | -5.13 | 4.253 | HILIC | -0.87 | 0.012556 | 1.28828 |
| Erythrose | C_4_H_8_O_4_ | [M-H]^-^ | 119.0346 | 120.0419 | -2.99 | 4.763 | HILIC | 0.39 | 0.036093 | 1.14509 |
| Uracil | C_4_H_4_N_2_O_2_ | [M-H]^-^ | 111.0197 | 112.0270 | -2.84 | 5.167 | HILIC | 1.00 | 1.15E-08 | 1.47233 |
| Ascorbic acid | C_6_H_8_O_6_ | [M-H]^-^ | 175.0239 | 176.0312 | -5.09 | 5.981 | HILIC | -0.70 | 0.000324 | 1.34647 |
| N-Acetyl-L-glutamic acid | C_7_H_11_NO_5_ | [M+H]^+^ | 190.0706 | 189.0633 | -2.41 | 6.485 | HILIC | -0.56 | 0.000347 | 1.01568 |
| Glutamic acid | C_5_H_9_NO_4_ | [M-H]^-^ | 146.0454 | 147.0527 | -3.41 | 6.809 | HILIC | 0.36 | 0.017723 | 1.46416 |
| N-Acetyl-L-2-aminoadipic acid | C_8_H_13_NO_5_ | [M-H]^-^ | 202.0710 | 203.0783 | -5.38 | 6.823 | HILIC | 0.91 | 2.75E-07 | 1.51022 |
| (2S,4S)-4-hydroxy-2,3,4,5-tetrahydrodipicolinic acid | C_7_H_9_NO_5_ | [M+H]^+^ | 188.0549 | 187.0476 | -2.49 | 7.878 | HILIC | 0.70 | 0.021561 | 1.32349 |
| Pyroglutamic acid | C_5_H_7_NO_3_ | [M+H]^+^ | 130.0500 | 129.0427 | 0.95 | 7.949 | HILIC | 2.15 | 0.049912 | 1.0674 |
| Thiomorpholine 3-carboxylate | C_5_H_9_NO_2_S | [M+H]^+^ | 148.0422 | 147.0349 | -3.31 | 8.249 | HILIC | 1.45 | 1.4E-05 | 1.54997 |
| Hydrocinnamic acid | C_9_H_10_O_2_ | [M+H]^+^ | 151.0749 | 150.0676 | -3.05 | 8.601 | HILIC | 1.33 | 0.005316 | 1.19372 |
| 2-Butynedioic acid | C_4_H_2_O_4_ | [M-H]^-^ | 112.9877 | 113.9948 | -4.40 | 8.790 | HILIC | -0.84 | 0.037807 | 1.46282 |
| Maleic acid | C_4_H_4_O_4_ | [M-H]^-^ | 115.0035 | 116.0102 | -6.78 | 8.984 | HILIC | 0.27 | 0.01763 | 1.1734 |
| L-Valinol | C_5_H_13_NO | [M+H]^+^ | 104.1067 | 103.0994 | -2.63 | 9.086 | HILIC | 0.58 | 0.01423 | 1.02479 |
| Xanthine | C_5_H_4_N_4_O_2_ | [M+H]^+^ | 153.0404 | 152.0331 | -2.20 | 9.842 | HILIC | 0.90 | 1.55E-05 | 1.39005 |
| Phenylephrine | C_9_H_13_NO_2_ | [M+H]^+^ | 168.1021 | 167.0948 | 1.08 | 10.016 | HILIC | 4.25 | 0.007661 | 1.37329 |
| Adenine | C_5_H_5_N_5_ | [M+H]^+^ | 136.0615 | 135.0543 | -1.82 | 10.203 | HILIC | 0.91 | 0.000324 | 1.26852 |
| Pyridoxal | C_8_H_9_NO_3_ | [M+H]^+^ | 168.0654 | 167.0581 | -1.03 | 10.468 | HILIC | 3.72 | 0.003396 | 1.33085 |
| Urocanic acid | C_6_H_5_N_2_O_2_ | [M+H]^+^ | 138.0425 | 137.0352 | 0.50 | 10.773 | HILIC | 1.25 | 3.86E-10 | 1.63813 |
| L-Prolyl-L-leucine | C_11_H_20_N_2_O_3_ | [M+H]^+^ | 229.1541 | 228.1468 | -2.44 | 10.812 | HILIC | 2.24 | 0.016003 | 1.34703 |
| Threonic acid | C_4_H_8_O_5_ | [M-H]^-^ | 135.0308 | 136.0380 | 6.34 | 11.328 | HILIC | 1.55 | 6.47E-13 | 1.74317 |
| Cinnamic acid | C_9_H_8_O_2_ | [M-H]^-^ | 147.0443 | 148.0516 | -5.63 | 12.512 | HILIC | 0.90 | 0.0002 | 1.48391 |
| D-Phenylalanine | C_9_H_11_NO_2_ | [M-H]^-^ | 164.0709 | 165.0782 | -4.75 | 12.514 | HILIC | 0.86 | 9.62E-06 | 1.47551 |
| Furan | C_4_H_4_O | [M+NH_4_]^+^ | 86.0600 | 68.0262 | -0.90 | 12.626 | HILIC | 1.62 | 0.017474 | 1.16285 |
| 8-Hydroxy-deoxyguanosine | C_10_H_13_N_5_O_5_ | [M-H]^-^ | 282.0824 | 283.0897 | -6.88 | 13.169 | HILIC | 0.75 | 0.007556 | 1.0439 |
| Benzoic acid | C_7_H_6_O_2_ | [M+H]^+^ | 123.0442 | 122.0369 | 0.84 | 13.410 | HILIC | 2.45 | 0.006375 | 1.2225 |
| Citrulline | C_6_H_13_N_3_O_3_ | [M+H]^+^ | 176.1029 | 175.0956 | -0.53 | 13.579 | HILIC | 4.60 | 0.000257 | 1.4708 |
| Cytidine 5'-diphosphate | C_9_H_15_N_3_O_11_P_2_ | [M-H]^-^ | 402.0092 | 403.0165 | -4.25 | 13.748 | HILIC | 0.55 | 7.55E-05 | 1.05455 |
| Cytosine | C_4_H_5_N_3_O | [M+H]^+^ | 112.0503 | 111.0430 | -2.11 | 13.865 | HILIC | -0.45 | 0.012543 | 1.17742 |
| Erythrose-4-phosphate | C_4_H_9_O_7_P | [M-H]^-^ | 199.0005 | 200.0077 | -4.27 | 14.129 | HILIC | 1.39 | 2.49E-06 | 1.65382 |
| L-Threonine | C_4_H_9_NO_3_ | [M-H]^-^ | 118.0509 | 119.0582 | -0.70 | 15.127 | HILIC | 0.34 | 0.000272 | 1.5657 |
| Glycerol 3-phosphate | C_3_H_9_O_6_P | [M-H]^-^ | 171.0060 | 172.0132 | -2.53 | 15.847 | HILIC | 1.81 | 2.2E-07 | 1.60792 |
| L-Aspartic acid | C_4_H_7_NO_4_ | [M-H]^-^ | 132.0302 | 133.0375 | -0.28 | 16.451 | HILIC | 1.14 | 0.005973 | 1.56859 |
| Adenosine monophosphate | C_10_H_14_N_5_O_7_P | [M-H]^-^ | 347.0574 | 348.0647 | -2.77 | 17.261 | HILIC | -0.72 | 1.33E-05 | 1.24939 |
| Ornithine | C_5_H_12_N_2_O_2_ | [M-H]^-^ | 131.0822 | 132.0894 | -3.35 | 17.348 | HILIC | 1.16 | 3.01E-12 | 1.48273 |
| 2-(6'-Methylthio)hexylmalic acid | C_11_H_20_O_5_S | [M+H]^+^ | 265.1116 | 264.1043 | 4.43 | 0.867 | RP-C18 | 1.94 | 0.031281 | 2.48369 |
| 4-Hydroxy-L-threonine | C_4_H_9_NO_4_ | [M+H]^+^ | 136.0615 | 135.0542 | 7.78 | 1.214 | RP-C18 | 0.67 | 0.024917 | 1.43299 |
| Hypoxanthine | C_5_H_4_N_4_O | [M+H]^+^ | 137.0457 | 136.0384 | -0.67 | 1.252 | RP-C18 | 2.67 | 0.004601 | 2.61683 |
| Adenosine | C_10_H_13_N_5_O_4_ | [M+H]^+^ | 268.1038 | 267.0965 | -0.86 | 1.913 | RP-C18 | 0.97 | 0.03716 | 2.45077 |
| p-Xylene | C_8_H_10_ | [M+H]^+^ | 107.0852 | 106.0779 | -3.40 | 5.713 | RP-C18 | -0.35 | 0.002229 | 1.42021 |
| Indole | C_8_H_7_N | [M+H]^+^ | 118.0648 | 117.0575 | -2.77 | 5.714 | RP-C18 | -0.40 | 0.049555 | 1.20425 |
| 7-Ethoxy-4-methyl-2H-1-benzopyran-2-one | C_12_H_12_O_3_ | [M+H]^+^ | 205.0853 | 204.0780 | -3.08 | 8.476 | RP-C18 | 0.31 | 0.001884 | 1.61212 |
| Eicosapentaenoic acid | C_20_H_30_O_2_ | [M-H]^-^ | 301.2154 | 302.2227 | -6.16 | 9.822 | RP-C18 | 1.12 | 2.59E-07 | 1.96713 |
| Docosahexaenoic acid | C_22_H_32_O_2_ | [M+H]^+^ | 329.2462 | 328.2390 | -3.85 | 10.533 | RP-C18 | 1.16 | 1.17E-08 | 2.42178 |
| Androstane | C_19_H_32_ | [M-H]^-^ | 259.2419 | 260.2492 | -4.79 | 10.736 | RP-C18 | 0.71 | 5.26E-06 | 1.83095 |
| Cinnamaldehyde | C_9_H_8_O | [M+H]^+^ | 133.0643 | 132.0570 | -3.96 | 10.919 | RP-C18 | -0.40 | 0.030873 | 1.20548 |
| Docosatetraenoic acid | C_22_H_36_O_2_ | [M-H]^-^ | 331.2629 | 332.2702 | -3.96 | 11.842 | RP-C18 | 2.06 | 2.15E-11 | 1.96988 |
| N-Hexadecanoylpyrrolidine | C_20_H_39_NO | [M+H]^+^ | 310.3091 | 309.3018 | -4.38 | 12.606 | RP-C18 | -0.39 | 0.039691 | 1.47385 |

**Table S2. Differential metabolites in liver tissues of 5xFAD mice (WT group vs AD group).**

| Identification | Formula | Reference ion | Measured [*m/z*] | Calculated [*m/z*] | Delta Mass [ppm] | RT [min] | Mode | Log2 FC: (WT) / (AD) | P-value: (WT) / (AD) | VIP: (WT) / (AD) |
| --- | --- | --- | --- | --- | --- | --- | --- | --- | --- | --- |
| 3,5-Di-tert-butyl-4-hydroxybenzaldehyde | C_15_H_22_O_2_ | [M-H]^-^ | 233.1532 | 234.1605 | -6.43 | 1.533 | HILIC | -0.66 | 1.77E-05 | 1.24026 |
| Docosapentaenoic acid | C_22_H_34_O_2_ | [M-H]^-^ | 329.2459 | 330.2532 | -8.13 | 1.646 | HILIC | -0.67 | 0.013601 | 1.51818 |
| Stearic acid | C_18_H_36_O_2_ | [M-H]^-^ | 283.2623 | 284.2696 | -6.75 | 1.688 | HILIC | -1.51 | 1.02E-06 | 1.3461 |
| 5-(Methylthio)-2,3-dioxopentyl phosphate | C_6_H_11_O_6_PS | [M-H]^-^ | 240.9928 | 242.0000 | -5.63 | 1.699 | HILIC | -1.34 | 0.005736 | 2.1169 |
| Lauramide | C_12_H_25_NO | [M+H]^+^ | 200.2001 | 199.1928 | -4.12 | 1.725 | HILIC | -1.04 | 1.98E-05 | 1.10302 |
| Styrene | C_8_H_8_ | [M+H]^+^ | 105.0693 | 104.0621 | -5.22 | 1.792 | HILIC | -0.92 | 4.51E-10 | 2.1768 |
| p-Xylene | C_8_H_10_ | [M+H]^+^ | 107.0850 | 106.0777 | -4.81 | 1.796 | HILIC | -1.08 | 7.24E-12 | 1.91448 |
| Arachidoyl Ethanolamide | C_22_H_45_NO_2_ | [M+H]^+^ | 356.3510 | 355.3437 | -3.62 | 2.190 | HILIC | -1.75 | 5.99E-05 | 2.09919 |
| Indole | C_8_H_7_N | [M+H]^+^ | 118.0647 | 117.0574 | -3.75 | 2.234 | HILIC | -0.32 | 0.02204 | 1.89798 |
| Threo-sphingosine | C_18_H_37_NO_2_ | [M+H]^+^ | 300.2886 | 299.2813 | -3.63 | 3.667 | HILIC | -1.89 | 1.36E-11 | 1.05223 |
| Nicotinamide | C_6_H_6_N_2_O | [M+H]^+^ | 123.0548 | 122.0476 | -3.82 | 4.231 | HILIC | -0.78 | 0.022706 | 1.18763 |
| D-Glucuronic acid | C_6_H_10_O_7_ | [M-H]^-^ | 193.0346 | 194.0418 | -4.18 | 4.267 | HILIC | -1.15 | 4.78E-05 | 1.51692 |
| Pyridine | C_5_H_5_N | [M+H]^+^ | 80.0490 | 79.0418 | -5.69 | 4.356 | HILIC | -5.90 | 1.46E-12 | 1.1981 |
| Laurylamine | C_12_H_27_N | [M+H]^+^ | 186.2211 | 185.2138 | -3.03 | 4.415 | HILIC | -2.56 | 1.68E-10 | 1.09271 |
| Palmitic amide | C_16_H_33_NO | [M+H]^+^ | 256.2628 | 255.2556 | -2.60 | 4.431 | HILIC | -3.61 | 4.73E-06 | 1.35035 |
| 2-Methylbutyrylcarnitine | C_12_H_23_NO_4_ | [M+H]^+^ | 246.1693 | 245.1621 | -2.70 | 4.516 | HILIC | -2.40 | 9.19E-08 | 1.7947 |
| Diethyl dicarbonate | C_6_H_10_O_5_ | [M-H]^-^ | 161.0449 | 162.0522 | -4.09 | 4.585 | HILIC | -0.61 | 0.011995 | 1.65726 |
| Sphinganine | C_18_H_39_NO_2_ | [M+H]^+^ | 302.3044 | 301.2971 | -3.23 | 4.586 | HILIC | -3.33 | 1.53E-12 | 2.0302 |
| Pyrimidine | C_4_H_4_N_2_ | [M+H]^+^ | 81.0443 | 80.0370 | -5.28 | 4.608 | HILIC | -2.80 | 1.94E-08 | 1.94674 |
| Zeatin | C_10_H_13_N_5_O | [M+H]^+^ | 220.1173 | 219.1100 | -9.25 | 5.250 | HILIC | -0.82 | 0.032031 | 1.12714 |
| Lactic acid | C_3_H_6_O_3_ | [M-H]^-^ | 89.0241 | 90.0314 | -3.83 | 5.570 | HILIC | 0.91 | 0.002817 | 1.55864 |
| Succinic acid | C_4_H_6_O_4_ | [M-H]^-^ | 117.0190 | 118.0263 | -2.92 | 5.609 | HILIC | 1.44 | 0.007165 | 1.2561 |
| DL-2-Aminoadipic acid | C_6_H_11_NO_4_ | [M-H]^-^ | 160.0611 | 161.0684 | -2.50 | 6.015 | HILIC | 0.59 | 0.043396 | 1.51176 |
| Glutaral | C_5_H_8_O_2_ | [M-H]^-^ | 99.0450 | 100.0522 | -1.90 | 6.040 | HILIC | -0.78 | 0.030585 | 1.9904 |
| Purine | C_5_H_4_N_4_ | [M-H]^-^ | 119.0361 | 120.0433 | -2.25 | 6.121 | HILIC | -4.24 | 0.043999 | 2.25251 |
| Glutamic acid | C_5_H_9_NO_4_ | [M-H]^-^ | 146.0454 | 147.0526 | -3.49 | 6.850 | HILIC | 0.77 | 0.00014 | 2.16039 |
| Adenine | C_5_H_5_N_5_ | [M-H]^-^ | 134.0466 | 135.0539 | -4.59 | 7.797 | HILIC | -1.17 | 3.01E-08 | 2.07615 |
| (R)-3-Hydroxybutyrylcarnitine | C_11_H_21_NO_5_ | [M+H]^+^ | 248.1484 | 247.1411 | -3.49 | 7.894 | HILIC | -1.82 | 0.00031 | 1.89609 |
| Oleamide | C_18_H_35_NO | [M+H]^+^ | 282.2777 | 281.2705 | -5.01 | 8.617 | HILIC | -1.42 | 1.44E-05 | 2.17852 |
| L-Alanine | C_3_H_7_NO_2_ | [M+H]^+^ | 90.0544 | 89.0471 | -6.23 | 9.044 | HILIC | -1.03 | 0.0118 | 1.27942 |
| Pterin | C_6_H_5_N_5_O | [M-H]^-^ | 162.0416 | 163.0489 | -3.30 | 9.795 | HILIC | -0.90 | 0.001891 | 1.57191 |
| Butane-2,3-dione | C_4_H_6_O_2_ | [M+H]^+^ | 87.0436 | 86.0363 | -5.42 | 9.975 | HILIC | 0.86 | 0.000878 | 1.5389 |
| Pyridoxal | C_8_H_9_NO_3_ | [M-H]^-^ | 166.0501 | 167.0574 | -5.02 | 9.983 | HILIC | -0.80 | 0.00092 | 1.46237 |
| Hypoxanthine | C_5_H_4_N_4_O | [M+H]^+^ | 137.0454 | 136.0381 | -2.99 | 10.088 | HILIC | 0.93 | 0.043402 | 1.03577 |
| 5-Hydroxyisourate | C_5_H_4_N_4_O_4_ | [M-H]^-^ | 183.0151 | 184.0224 | -4.63 | 10.213 | HILIC | 3.24 | 2.12E-08 | 1.79698 |
| Pregnanetriol | C_21_H_36_O_3_ | [M+H]^+^ | 337.2721 | 336.2649 | -4.74 | 10.428 | HILIC | -0.52 | 0.010315 | 1.59856 |
| Methyl 9,10-epoxystearate | C_19_H_36_O_3_ | [M+H]^+^ | 313.2723 | 312.2650 | -4.60 | 10.452 | HILIC | -1.84 | 8.86E-09 | 1.69905 |
| D-Mannoheptulose | C_7_H_14_O_7_ | [M-H]^-^ | 209.0656 | 210.0728 | -5.34 | 10.644 | HILIC | 0.68 | 6.3E-05 | 1.6155 |
| 4-Trimethylammoniobutanoic acid | C_7_H_15_NO_2_ | [M+H]^+^ | 146.1170 | 145.1097 | -3.98 | 11.044 | HILIC | 2.58 | 0.001544 | 1.59727 |
| 4-Guanidinobutanoic acid | C_5_H_11_N_3_O_2_ | [M+H]^+^ | 146.0919 | 145.0846 | -3.79 | 11.136 | HILIC | 2.36 | 0.010839 | 1.99791 |
| Valine | C_5_H_11_NO_2_ | [M+H]^+^ | 118.0857 | 117.0784 | -4.60 | 11.758 | HILIC | 2.43 | 4.29E-06 | 1.69923 |
| Malic acid | C_4_H_6_O_5_ | [M-H]^-^ | 133.0138 | 134.0210 | -3.61 | 11.919 | HILIC | 1.16 | 0.007944 | 2.314 |
| L-Carnitine | C_7_H_15_NO_3_ | [M+H]^+^ | 162.1120 | 161.1048 | -2.70 | 11.998 | HILIC | -5.50 | 0.017067 | 2.26922 |
| Leucine | C_6_H_13_NO_2_ | [M-H]^-^ | 130.0868 | 131.0941 | -3.89 | 12.071 | HILIC | -4.22 | 0.003045 | 1.71827 |
| Tranexamic acid | C_8_H_15_NO_2_ | [M+H]^+^ | 158.1169 | 157.1095 | -4.75 | 12.537 | HILIC | 0.91 | 0.016347 | 2.29002 |
| Threonic acid | C_4_H_8_O_5_ | [M+H]^+^ | 137.0452 | 136.0379 | 5.57 | 12.923 | HILIC | -0.65 | 6.43E-05 | 2.06011 |
| 4-Hydroxy-L-threonine | C_4_H_9_NO_4_ | [M+H]^+^ | 136.0612 | 135.0539 | 5.63 | 12.961 | HILIC | -0.51 | 2.91E-06 | 2.54034 |
| p-Aminobenzoic acid | C_7_H_7_NO_2_ | [M+H]^+^ | 138.0544 | 137.0471 | -4.18 | 13.068 | HILIC | 2.01 | 4.32E-07 | 1.98581 |
| 3-Hydroxy-L-proline | C_5_H_9_NO_3_ | [M+H]^+^ | 132.0650 | 131.0577 | -3.86 | 14.681 | HILIC | 0.86 | 0.016494 | 1.52012 |
| Gluconic acid | C_6_H_12_O_7_ | [M-H]^-^ | 195.0504 | 196.0577 | -3.24 | 14.731 | HILIC | 1.09 | 0.026576 | 2.44254 |
| Pyroglutamic acid | C_5_H_7_NO_3_ | [M-H]^-^ | 128.0350 | 129.0423 | -2.63 | 15.417 | HILIC | 0.57 | 0.009163 | 1.83053 |
| Glutamine | C_5_H_10_N_2_O_3_ | [M-H]^-^ | 145.0614 | 146.0687 | -2.79 | 15.424 | HILIC | 0.38 | 0.048845 | 1.96662 |
| Phosphorylcholine | C_5_H_14_NO_4_P | [M+H]^+^ | 184.0727 | 183.0654 | -3.37 | 15.921 | HILIC | -1.14 | 0.048027 | 1.77377 |
| Taurine | C_2_H_7_NO_3_S | [M-H]^-^ | 124.0074 | 125.0147 | 0.37 | 0.974 | RP-C18 | -1.73 | 0.00274 | 1.47236 |
| L-Sorbose | C_6_H_12_O_6_ | [M+Cl]^-^ | 215.0325 | 180.0632 | -1.34 | 1.000 | RP-C18 | -1.56 | 0.001065 | 1.69988 |
| gamma-Aminobutyric acid | C_4_H_9_NO_2_ | [M+H]^+^ | 104.0705 | 103.0632 | -1.49 | 1.020 | RP-C18 | 0.54 | 0.001127 | 1.51793 |
| Xanthine | C_5_H_4_N_4_O_2_ | [M-H]^-^ | 151.0264 | 152.0336 | 1.31 | 1.214 | RP-C18 | -0.39 | 0.004871 | 1.91836 |
| Valinol | C_5_H_13_NO | [M+H]^+^ | 104.1067 | 103.0994 | -2.62 | 1.248 | RP-C18 | -1.45 | 1.11E-07 | 1.5125 |
| Piperidine | C_5_H_11_N | [M+H]^+^ | 86.0962 | 85.0889 | -3.21 | 2.600 | RP-C18 | 0.52 | 0.000172 | 1.62517 |
| Azelaic acid | C_9_H_16_O_4_ | [M-H]^-^ | 187.0974 | 188.1047 | -0.86 | 3.443 | RP-C18 | -0.45 | 2.73E-09 | 2.38719 |
| Sebacic acid | C_10_H_18_O_4_ | [M-H]^-^ | 201.1129 | 202.1202 | -1.53 | 3.679 | RP-C18 | -0.69 | 2.55E-07 | 1.6129 |
| 4-Nitrophenol | C_6_H_5_NO_3_ | [M-H]^-^ | 138.0197 | 139.0269 | -0.06 | 3.810 | RP-C18 | -0.44 | 0.002521 | 1.37556 |
| 5-Hydroxymethyl-2-furancarboxaldehyde | C_6_H_6_O_3_ | [M+H]^+^ | 127.0386 | 126.0313 | -2.82 | 5.652 | RP-C18 | 0.45 | 0.001464 | 1.40698 |
| 11-(Dimethylamino)-2-ethoxy-3-hydroxypregnan-20-one | C_25_H_43_NO_3_ | [M+H]^+^ | 406.3306 | 405.3233 | -2.49 | 5.659 | RP-C18 | 0.82 | 0.029811 | 1.10595 |
| Eicosapentaenoic acid | C_20_H_30_O_2_ | [M-H]^-^ | 301.2163 | 302.2236 | -3.27 | 9.737 | RP-C18 | -0.92 | 0.000121 | 1.64568 |
| Docosahexaenoic acid | C_22_H_32_O_2_ | [M-H]^-^ | 327.2326 | 328.2399 | -1.15 | 10.380 | RP-C18 | -0.44 | 0.016976 | 1.00928 |
| Isopropyl 6-O-[(2R,3R,4R)-3,4-dihydroxy-4-(hydroxymethyl)tetrahydro-2-furanyl]-D-glucopyranoside | C_14_H_26_O_10_ | [M-H]^-^ | 353.1419 | 354.1492 | -9.74 | 10.462 | RP-C18 | -2.06 | 0.000138 | 1.4325 |
| (2S)-2-({N-[(2S)-2-Ammonio-4-methylpentanoyl]-L-leucyl}amino)-3-phenylpropanoate | C_21_H_33_N_3_O_4_ | [M+H]^+^ | 392.2569 | 391.2496 | 6.46 | 10.502 | RP-C18 | 0.45 | 0.001445 | 1.53359 |

**Table S3. Tentative identification of compounds in HLJDD.**

| **No** | **Ion mode** | **Retention time (min)** | **Formula** | **Theoretical *m/z*** | **Measured *m/z*** | **Mass Error (ppm)** | **Fragment Ions** | **Adducts** | **Compound** | **Sourse** |
| --- | --- | --- | --- | --- | --- | --- | --- | --- | --- | --- |
| 1 | POS | 0.75 | C6H13NO5 | 180.0867 | 180.0866 | -0.56 | 60.0449, 72.0450, 84.0452, 85.0288, 96.0450, 98.0605, 114.0558, 126.0544, 144.0653, 162.0756 | M+H | Manosamine | P, C, G, S |
| 2 | POS | 0.77 | C8H15NO6 | 204.0872 | 204.0863 | -4.65 | 70.0656, 103.0390, 114.0557, 158.0821 | M+H-H2O | *N*-Acetyl-D-glucosamine | P |
| 3 | POS | 0.77 | C5H11NO2 | 118.0863 | 118.0864 | 0.85 | 58.0660, 59.0735 | M+H | Norvaline | P, S |
| 4 | NEG | 0.78 | C4H8O5 | 135.0299 | 135.0289 | -7.78 | 59.0127, 72.9922, 75.0079, 87.0080, 89.0236 | M-H | Threonic acid | P, C, S |
| 5 | POS | 0.79 | C6H12O6 | 203.0526 | 203.0527 | 0.3 |  | M+Na | Glucose | P, C, G, S |
| 6 | POS | 0.81 | C5H9NO2 | 116.0706 | 116.0707 | 1.21 | 70.0657 | M+H | Pyrrolidine-2-carboxylic acid | P |
| 7 | NEG | 0.83 | C12H22O11 | 341.1089 | 341.1085 | -1.2 | 59.0128, 71.0132, 89.0236, 101.0232, 113.0239, 119.0333, 161.0450, 179.0547, 221.0677 | M-H | Turanose | P, G, S |
| 8 | NEG | 0.97 | C4H6O5 | 133.0143 | 133.0132 | -7.67 | 71.0131, 72.9923, 89.0236, 115.0025 | M-H | Malic acid | P |
| 9 | NEG | 1 | C12H18O11 | 383.0831 | 383.0838 | 1.83 |  | M+FA-H | 2-*O*-β-D-Glucopyranosyl-L-ascorbic acid | G, S, P |
| 10 | POS | 1.04 | C6H11NO2 | 130.0863 | 130.0866 | 2.54 | 70.0656, 71.0498, 84.0815 | M+H | Pipecolinic acid | P |
| 11 | NEG | 1.11 | C7H10O5 | 173.0456 | 173.0448 | -4.33 | 73.0284, 83.0494, 93.0332, 99.0079, 99.0439, 111.0081, 111.0445, 137.0243, 155.0341 | M-H | Shikimic acid | G |
| 12 | NEG | 1.16 | C10H18O9 | 281.0878 | 281.088 | 0.53 | 59.0130, 71.0127, 85.0285, 101.0235, 113.0229, 205.0698 | M-H | Xylobiose | G, S |
| 13 | NEG | 1.24 | C6H12O5 | 327.1297 | 327.1299 | 0.7 | 59.0129, 71.0126, 89.0237, 101.0233, 119.0335, 133.0133, 165.0770, 179.0550 | 2M-H | Fucose | P |
| 14 | POS | 1.41 | C6H5NO2 | 124.0393 | 124.0394 | 0.89 | 80.0501, 96.0451 | M+H | Niacin | P |
| 15 | POS | 1.63 | C6H6O6 | 157.0137 | 157.0131 | -4.27 | 68.9977, 97.0286, 111.0078, 112.0877, 129.0179, 139.0020, 152.0341, 152.9944 | M+H-H2O | Aconitic acid | P |
| 16 | NEG | 3.7 | C7H6O5 | 169.0143 | 169.0137 | -3.31 | 122.8932, 125.0234 | M-H | Gallic acid | G, S, P, C |
| 17 | NEG | 7.13 | C7H6O4 | 153.0193 | 153.0186 | -4.57 | 109.0288 | M-H | Protocatechuic acid | G, S, P, C |
| 18 | NEG | 7.76 | C8H10O3 | 153.0557 | 153.0547 | -6.6 | 109.0288, 123.0439 | M-H | Hydroxytyrosol | S, C |
| 19 | NEG | 7.8 | C16H22O10 | 373.114 | 373.1139 | -0.29 | 59.0129, 71.0126, 89.0237, 101.0234, 119.0336, 123.0439, 149.0603, 167.0714, 193.0507 | M-H | Gardoside | G |
| 20 | POS | 7.91 | C15H14O7 | 289.0713 | 289.0703 | -3.25 |  | M+H-H2O | Gallocatechin | S |
| 21 | NEG | 7.98 | C16H18O9 | 353.0878 | 353.0879 | 0.11 |  | M-H | 1-Caffeoylquinic acid | G |
| 22 | NEG | 8.72 | C16H22O10 | 373.114 | 373.1139 | -0.32 | 59.0129, 71.0126, 89.0237, 123.0438, 149.0602, 167.0711, 193.0505, 211.0615 | M-H | Geniposidic acid | G, C |
| 23 | POS | 8.72 | C14H18O9 | 353.0843 | 353.0845 | 0.51 |  | M+Na | Vanillic acid 4-β-D-glucopyranoside | P, C, G, S |
| 24 | NEG | 9.13 | C17H24O11 | 449.1301 | 449.1302 | 0.24 | 101.0234, 139.0386, 179.0550, 241.0701, 403.1205 | M+FA-H | Methyl deacetylasperulosidate | G, P |
| 25 | NEG | 9.35 | C16H18O9 | 353.0878 | 353.0877 | -0.23 | 135.0437, 179.0343, 191.0562 | M-H | Neochlorogenic acid | P, C, G, S |
| 26 | POS | 9.41 | C10H13NO3 | 213.1234 | 213.1235 | 0.7 |  | M+NH4 | *N*-Acetyldopamine | P, C |
| 27 | NEG | 9.58 | C17H24O11 | 449.1301 | 449.1302 | 0.33 | 89.0237, 101.0233, 127.0388, 191.0336, 223.0609, 241.0699, 403.1203 | M+FA-H | Gardenoside | P, G, S |
| 28 | POS | 9.64 | C10H12O5 | 213.0758 | 213.0758 | 0.38 |  | M+H | Geniposidic acid aglycone | G |
| 29 | NEG | 9.77 | C16H24O10 | 375.1297 | 375.1291 | -1.47 | 59.0129, 71.0126, 89.0237, 107.0495, 125.0594, 151.0752, 169.0853, 213.0772 | M-H | Mussaenosidic acid | C, G |
| 30 | NEG | 9.87 | C14H18O8 | 313.0929 | 313.0936 | 2.33 |  | M-H | Glucovanillin | P, C |
| 31 | POS | 10.04 | C11H14O6 | 225.0763 | 225.0757 | -2.80 | 67.0542, 91.0543, 95.0491, 119.0492, 123.0440, 147.0441, 165.0545, 175.0392, 189.0548, 193.0496 | M+H-H2O | Genipinic acid | G |
| 32 | NEG | 10.21 | C17H24O11 | 449.1301 | 449.1301 | 0.16 | 101.0232, 139.0385, 241.0698, 403.1287 | M+FA-H | Feretoside | G |
| 33 | POS | 10.28 | C16H26O8 | 369.152 | 369.1519 | -0.35 | 227.6267, 369.1520 | M+Na | Jasminoside | G |
| 34 | NEG | 10.36 | C15H16O9 | 339.0722 | 339.0718 | -1.09 |  | M-H | Cichoriin | G, P |
| 35 | NEG | 10.49 | C8H8O5 | 183.0299 | 183.0295 | -2.19 |  | M-H | Methyl gallate | S |
| 36 | NEG | 10.78 | C9H10O4 | 227.0561 | 227.0563 | 0.7 |  | M+FA-H | Dihydrocaffeic acid | G |
| 37 | NEG | 11.26 | C16H20O9 | 355.1035 | 355.1036 | 0.42 | 85.0286, 134.0370, 149.0604, 178.0256, 191.0196, 193.0505 | M-H | Trans-ferulic acid-4-β-glucoside | G, S |
| 38 | NEG | 11.46 | C7H6O2 | 121.0295 | 121.0286 | -7.11 | 94.0286 | M-H | p-Hydroxybenzaldehyde | G |
| 39 | NEG | 11.52 | C17H24O11 | 403.1246 | 403.1245 | -0.25 | 59.0128, 61.9870, 89.0228, 109.0288, 173.0430, 181.0493, 191.0563, 226.0480, 241.0702 | M-H | 10-Hydroxymajoroside | S, P, C |
| 40 | POS | 11.64 | C16H18O9 | 377.0843 | 377.0834 | -2.52 |  | M+Na | Chlorogenic acid | G, S, P, C |
| 41 | NEG | 11.72 | C21H26O12 | 515.1406 | 515.1415 | 1.75 |  | M+FA-H | Umbelliferone 7-*O*-rutinoside | G |
| 42 | NEG | 11.99 | C9H8O4 | 179.035 | 179.0342 | -4.58 | 135.0438 | M-H | Caffeic acid | G, S, P, C |
| 43 | NEG | 12.09 | C17H20O9 | 367.1035 | 367.1038 | 0.98 | 134.0369, 193.0505 | M-H | 5-Feruloylquinic acid | G, P, C |
| 44 | NEG | 12.1 | C16H18O9 | 353.0878 | 353.0878 | -0.08 | 93.0332, 135.0436, 173.0453, 179.0341, 191.0561 | M-H | Cryptochlorogenic acid | P, C, G, S |
| 45 | NEG | 12.22 | C23H34O15 | 595.188 | 595.1894 | 2.4 | 68.9971, 101.0233, 123.0438, 207.0669, 225.0767, 549.1825 | M+FA-H | Genipin 1-β-D-gentiobioside | G, P |
| 46 | POS | 12.7 | C9H12O4 | 185.0808 | 185.0808 | -0.11 | 121.1012, 125.0596, 125.0961, 137.0961, 139.1115, 153.0545, 154.0625, 167.1066, 185.0806, 185.1169 | M+H | 4-(Hydroxymethyl)-2,6-dimethoxyphenol | G |
| 47 | POS | 12.98 | C20H24NO4+ | 342.17 | 342.1692 | -2.34 | 192.1023 | M+ | Phellodendrine | P, C |
| 48 | POS | 12.99 | C17H20O9 | 391.1 | 391.0996 | -0.87 | 177.0555, 197.0429, 199.0355, 215.0549, 256.0779, 373.0914 | M+Na | 3-*O*-Caffeoylquinic acid methyl ester | G, S |
| 49 | NEG | 13.07 | C16H20O9 | 401.1089 | 401.1095 | 1.35 | 71.0128, 85.0286, 101.0236, 161.0454, 178.0256, 193.0506, 221.0821, 269.1026, 355.1023 | M+FA-H | Cis-Ferulic acid 4-*O*-β-D-glucopyranoside | P, C |
| 50 | POS | 13.08 | C10H10O5 | 211.0601 | 211.0604 | 1.52 |  | M+H | 5-Hydroxyferulic acid | G, S, C |
| 51 | POS | 13.33 | C17H24O10 | 411.1262 | 411.1268 | 1.46 |  | M+Na | Geniposide | G, S, P, C |
| 52 | NEG | 13.62 | C15H14O6 | 335.0772 | 335.0779 | 1.85 |  | M+FA-H | Epicatechin | P, S |
| 53 | NEG | 13.65 | C16H18O8 | 337.0929 | 337.0935 | 1.69 | 93.0332, 163.0399, 173.0452, 191.0561 | M-H | 5-*O*-(E)-p-Coumaroylquinic acid | G |
| 54 | POS | 13.82 | C8H8O3 | 153.0546 | 153.0548 | 0.91 |  | M+H | Methyl Salicylate | P, C |
| 55 | POS | 13.91 | C20H24NO4+ | 342.17 | 342.1691 | -2.57 |  | M+ | Magnoflorine | P, C |
| 56 | NEG | 14.06 | C17H24O10 | 433.1352 | 433.1353 | 0.39 | 101.0233, 123.0439, 225.0771, 387.1308 | M+FA-H | Verbenalin | P |
| 57 | NEG | 14.15 | C17H24O10 | 433.1352 | 433.1339 | -2.86 | 101.0236, 123.0448, 147.0448, 207.0660, 225.0765, 227.6272 | M+FA-H | Gardoside methyl ester | G |
| 58 | NEG | 14.22 | C16H16O8 | 335.0772 | 335.0778 | 1.7 | 135.0438, 161.0233, 179.0345, 213.5052 | M-H | 5-*O*-Caffeoylshikimic acid | G, C |
| 59 | NEG | 14.57 | C9H8O3 | 163.0401 | 163.0395 | -3.37 |  | M-H | p-Coumaric acid | C, S |
| 60 | NEG | 14.74 | C20H22O8 | 371.1131 | 371.1136 | 1.37 |  | M-H2O-H | Resveratroloside | S |
| 61 | NEG | 14.76 | C17H20O9 | 367.1035 | 367.1038 | 0.9 | 93.0332, 134.0368, 173.0453, 191.0562, 193.0504 | M-H | 4-Feruloylquinic acid | G, S, P, C |
| 62 | POS | 15.22 | C9H10O4 | 183.0652 | 183.0654 | 1.37 |  | M+H | Syringaldehyde | P, C, G, S |
| 63 | POS | 15.32 | C32H42O16 | 705.2365 | 705.2384 | 2.69 |  | M+Na | Pinoresinol diglucoside | P, C |
| 64 | NEG | 15.56 | C21H22O11 | 449.1089 | 449.1095 | 1.29 | 57.0333, 125.0238, 161.0238, 214.0378, 287.0563 | M-H | Taxifolin 7-*O*-rhamnoside | S |
| 65 | NEG | 15.72 | C21H20O11 | 447.0933 | 447.0938 | 1.03 |  | M-H | Isoorientin | S |
| 66 | POS | 15.8 | C26H28O14 | 587.1371 | 587.1394 | 3.82 | 569.1317 | M+Na | Schaftoside | S |
| 67 | POS | 15.83 | C21H20O11 | 449.1078 | 449.1082 | 0.85 |  | M+H | Orientin | S |
| 68 | NEG | 16.02 | C28H38O13 | 627.2294 | 627.2316 | 3.38 | 123.0439, 167.0342, 213.5454, 213.5722, 297.0627, 301.9368, 419.1712, 581.2175 | M+FA-H | Lyoniresinol 9'-*O*-glucoside | G, S |
| 69 | POS | 16.09 | C17H20O9 | 391.1 | 391.0999 | -0.05 |  | M+Na | Methyl chlorogenate | G |
| 70 | POS | 16.13 | C10H10O4 | 195.0652 | 195.0652 | 0.26 | 145.0280, 149.0602, 177.0554, 186.0561 | M+H | Ferulic acid | S, P, C |
| 71 | NEG | 16.14 | C15H10O7 | 301.0354 | 301.0359 | 1.79 | 125.0239, 139.0385, 148.0148, 149.0232, 151.0033, 161.0235, 193.0131, 220.7895 | M-H | Viscidulin I | S |
| 72 | NEG | 16.19 | C11H11NO3 | 204.0666 | 204.0662 | -1.96 | 72.9917, 116.0489, 142.0649, 158.0599, 160.9662, 186.0540 | M-H | Indole-3-lactic acid | P |
| 73 | POS | 16.32 | C20H23NO4 | 342.17 | 342.1705 | 1.61 |  | M+H | Isocorydine | P |
| 74 | POS | 16.35 | C19H21NO4 | 328.1543 | 328.1547 | 1.04 | 58.0651, 121.0284, 205.0649, 222.0679, 227.6224, 233.0595, 237.0907, 265.0853, 297.1125, 328.1548 | M+H | Norisocorydine | C |
| 75 | NEG | 16.46 | C30H24O12 | 621.125 | 621.1256 | 1.01 |  | M+FA-H | Procyanidin A2 | S |
| 76 | NEG | 16.47 | C15H12O6 | 333.0616 | 333.0623 | 2.1 |  | M+FA-H | 7,8,3',4'-Tetrahydroxyflavanone | S |
| 77 | NEG | 16.47 | C21H20O11 | 447.0933 | 447.0937 | 0.92 |  | M-H | Luteolin 5-*O*-glucoside | S |
| 78 | NEG | 16.55 | C27H30O16 | 609.1461 | 609.1471 | 1.61 | 215.4270, 271.0235, 300.0256, 301.0377 | M-H | Quercetin 3-*O*-neohesperidoside | S |
| 79 | POS | 16.61 | C15H10O6 | 287.055 | 287.0547 | -1.05 |  | M+H | 7,8,3′,4′-Tetrahydroxyflavone | S |
| 80 | POS | 16.63 | C11H12O5 | 225.0758 | 225.0758 | 0.13 | 119.0490, 147.0449, 175.0392, 181.1220, 192.0423, 207.0645 | M+H | Sinapinic acid | P, G, S |
| 81 | NEG | 16.66 | C33H40O19 | 739.2091 | 739.2087 | -0.58 | 227.0363, 255.0323, 284.0312, 285.0420, 301.9481 | M-H | Mauritianin | G |
| 82 | POS | 16.81 | C33H40O19 | 741.2237 | 741.2232 | -0.69 |  | M+H | Clitorin | S |
| 83 | NEG | 16.84 | C9H7NO | 144.0455 | 144.0447 | -5.83 |  | M-H | Indole-3-carboxaldehyde | P |
| 84 | POS | 17.02 | C17H17NO4 | 322.105 | 322.108 | 9.5 | 294.1104 | M+Na | *N*-p-Coumaroyl-octopamine | G |
| 85 | NEG | 17.05 | C25H26O13 | 579.1355 | 579.1374 | 3.23 |  | M+FA-H | Apigenin-6-*C*-β-D-xylopyranosyl-8-*C*-α-L-arabinopyranoside | G |
| 86 | POS | 17.1 | C21H20O12 | 487.0847 | 487.0852 | 0.94 | 311.0507, 352.0779 | M+Na | Myricitrin | S |
| 87 | POS | 17.14 | C15H10O7 | 303.0499 | 303.0499 | -0.16 | 167.0348 | M+H | Robinetin | S |
| 88 | NEG | 17.28 | C10H10O4 | 193.0506 | 193.0502 | -2.07 | 117.0336, 134.0369, 137.0229, 149.0603, 178.0254 | M-H | Trans-Isoferulic acid | P, G, S |
| 89 | NEG | 17.38 | C27H30O16 | 609.1461 | 609.147 | 1.38 | 271.0234, 300.0257, 301.0377 | M-H | Rutin | G, S |
| 90 | NEG | 17.45 | C26H28O13 | 547.1457 | 547.1467 | 1.74 | 337.0721, 367.0854 | M-H | Chrysin 6-*C*-arabinoside 8-*C*-glucoside | S, G, C |
| 91 | NEG | 17.53 | C21H20O11 | 493.0988 | 493.0997 | 1.89 |  | M+FA-H | 2-(3,4-Dihydroxyphenyl)-7-(β-D-glucopyranosyloxy)-8-hydroxy-4H-1-benzopyran-4-one | G |
| 92 | NEG | 17.57 | C21H20O10 | 431.0984 | 431.0984 | 0.14 | 165.0179, 216.8903, 233.0433, 283.0583, 311.0541, 327.0890, 387.1451, 413.1204 | M-H | Isovitexin | S |
| 93 | POS | 17.63 | C21H21NO6 | 406.1261 | 406.1289 | 6.87 | 332.0948 | M+Na | Hydrastine | C |
| 94 | POS | 17.81 | C20H23NO4 | 342.17 | 342.1704 | 1.32 | 178.0865, 192.1024 | M+H | Corypalmine | P |
| 95 | POS | 17.89 | C21H18O12 | 463.0871 | 463.0873 | 0.48 | 287.0555 | M+H | Luteolin 7-*O*-glucuronide | S |
| 96 | POS | 18.02 | C19H16NO4+ | 322.1074 | 322.1068 | -1.8 | 307.0851 | M+ | Groenlandicine | C |
| 97 | POS | 18.25 | C27H30O15 | 595.1658 | 595.1661 | 0.66 | 85.0291, 287.0558, 449.1099 | M+H | Biorobin | S |
| 98 | NEG | 18.26 | C29H36O15 | 623.1981 | 623.1995 | 2.21 | 113.0228, 133.0282, 135.0437, 161.0232, 461.1631 | M-H | Verbascoside | S, C |
| 99 | NEG | 18.29 | C26H28O13 | 547.1457 | 547.1466 | 1.66 | 337.0721, 367.0849 | M-H | Chrysin 6-*C*-glucoside 8-*C*-arabinoside | S, G |
| 100 | NEG | 18.39 | C26H36O11 | 523.2185 | 523.218 | -1.01 |  | M-H | Secoisolariciresinol monoglucoside | G, P, C |
| 101 | POS | 18.44 | C18H19NO5 | 352.1155 | 352.118 | 7.07 | 308.0928, 336.0864, 337.0940 | M+Na | *N*-Feruloyloctopamine | G |
| 102 | POS | 18.59 | C9H8O3 | 147.0446 | 147.0441 | -3.74 | 91.0542, 119.0491, 147.0441 | M+H-H2O | 2-Hydroxycinnamic acid | P |
| 103 | NEG | 18.65 | C21H20O12 | 463.0882 | 463.0866 | -3.52 | 151.0033, 227.6284, 243.0300, 255.0293, 271.0249, 300.0274, 301.0344, 463.0880 | M-H | Isoquercitrin | G, P |
| 104 | NEG | 18.7 | C8H8O4 | 335.0772 | 335.0779 | 1.82 |  | 2M-H | 2-Hydroxy-6-methoxybenzoic acid | G |
| 105 | POS | 18.7 | C27H30O14 | 601.1528 | 601.1526 | -0.35 |  | M+Na | Kaempferitrin | P, G |
| 106 | POS | 18.72 | C10H8O3 | 177.0546 | 177.0546 | 0.11 | 105.0703, 117.0336, 145.0294, 149.0598, 158.0607, 159.0444 | M+H | 4-Methylumbelliferone | G |
| 107 | POS | 18.82 | C27H32O14 | 563.1765 | 563.1754 | -2.06 | 125.0239, 207.0658, 213.5692, 383.1157, 401.1214, 407.1114, 509.1476, 527.1602, 545.1722 | M+H-H2O | Narirutin | G, S, P |
| 108 | NEG | 18.83 | C25H24O12 | 515.1195 | 515.1197 | 0.29 | 135.0440, 155.0344, 161.0234, 173.0455, 179.0345, 191.0564, 335.0756, 353.0868 | M-H | 3,4-Dicaffeoylquinic acid | G |
| 109 | POS | 18.87 | C21H25NO5 | 354.1706 | 354.1695 | -2.94 | 338.1392, 339.1495 | M+H-H2O | Epicorynoxidine | C |
| 110 | NEG | 18.9 | C26H32O11 | 519.1872 | 519.188 | 1.56 | 136.0160, 151.0394, 213.6455, 357.1376 | M-H | Pinoresinol 4-*O*-β-D-glucopyranoside | G, S, C |
| 111 | POS | 18.93 | C21H20O11 | 449.1078 | 449.1078 | -0.07 |  | M+H | Trifolin | S |
| 112 | NEG | 18.94 | C27H30O15 | 593.1512 | 593.1527 | 2.5 | 135.0437, 217.6572, 284.0319, 285.0425 | M-H | Nicotiflorin | G |
| 113 | NEG | 19.21 | C29H36O15 | 623.1981 | 623.2003 | 3.39 | 113.0228, 135.0438, 161.0232, 461.1626 | M-H | Isoacteoside | S, C |
| 114 | POS | 19.28 | C25H24O12 | 499.1241 | 499.1241 | 0.08 | 163.0387, 319.0825 | M+H-H2O | 3,5-*O*-Dicaffeoylquinic acid | G |
| 115 | POS | 19.29 | C27H30O15 | 617.1477 | 617.1471 | -1.02 | 100.1121 | M+Na | Oroxin B | S |
| 116 | POS | 19.34 | C28H32O16 | 625.1763 | 625.177 | 1.14 |  | M+H | Narcissin | G |
| 117 | NEG | 19.34 | C26H28O14 | 563.1406 | 563.1407 | 0.14 |  | M-H | Apiin | P, S |
| 118 | POS | 19.41 | C19H16NO4+ | 322.1074 | 322.1071 | -0.93 | 227.6256, 279.0890, 307.0842, 322.1066 | M+ | Thalifendine | P |
| 119 | NEG | 19.42 | C21H20O11 | 447.0933 | 447.0936 | 0.67 | 227.0360, 255.0282, 284.0315, 285.0413 | M-H | Astragalin | G, S |
| 120 | NEG | 19.45 | C17H17NO4 | 298.1085 | 298.1084 | -0.4 |  | M-H | N-trans-Caffeoyltyramine | P |
| 121 | POS | 19.48 | C11H14O3 | 177.0916 | 177.0908 | -4.52 |  | M+H-H2O | 3,4-Dimethoxycinnamyl alcohol | G |
| 122 | NEG | 19.48 | C28H36O13 | 579.2083 | 579.2102 | 3.18 |  | M-H | Episyringaresinol 4'-*O*-β-D-glncopyranoside | P |
| 123 | NEG | 19.48 | C21H20O10 | 431.0984 | 431.0983 | -0.14 |  | M-H | Apigenin 7-glucoside | S |
| 124 | POS | 19.51 | C21H23NO5 | 370.1649 | 370.1651 | 0.57 |  | M+H | Allocryptopine | P, C |
| 125 | POS | 19.53 | C22H22O12 | 461.1084 | 461.1094 | 2 |  | M+H-H2O | Isorhamnetin 3-*O*-galactoside | S |
| 126 | NEG | 19.59 | C15H12O6 | 333.0616 | 333.0623 | 2.07 |  | M+FA-H | Dihydrokaempferol | P, S |
| 127 | POS | 19.63 | C26H32O11 | 543.1837 | 543.1846 | 1.6 |  | M+Na | Epipinoresinol-4-*O*-β-D-glucoside | P, C |
| 128 | POS | 19.63 | C22H22O12 | 479.1184 | 479.12 | 3.4 |  | M+H | Isorhamnetin-3-*O*-glucoside | S |
| 129 | NEG | 19.63 | C15H10O6 | 285.0405 | 285.041 | 1.79 |  | M-H | Fisetin | S |
| 130 | POS | 19.7 | C21H20O11 | 431.0979 | 431.0984 | 1.14 |  | M+H-H2O | Kaempferol-7-*O*-β-D-glucopyranoside | S |
| 131 | POS | 19.95 | C22H27NO4 | 370.2013 | 370.2014 | 0.27 |  | M+H | Corydaline | C |
| 132 | NEG | 19.97 | C10H12O5 | 211.0612 | 211.0606 | -2.8 |  | M-H | Methyl syringate | G |
| 133 | NEG | 20.03 | C25H24O12 | 515.1195 | 515.1196 | 0.21 | 135.0438, 173.0455, 179.0344, 191.0564, 353.0866 | M-H | Isochlorogenic acid C | G |
| 134 | POS | 20.03 | C20H20NO4+ | 338.1387 | 338.1371 | -4.76 | 294.1100, 322.1069, 323.1146 | M+ | Columbamine | G, S, P, C |
| 135 | POS | 20.05 | C22H20O12 | 477.1028 | 477.1032 | 0.92 | 286.0458, 301.0701 | M+H | DiosMetin 7-*O*-β-D-Glucuronide | S |
| 136 | POS | 20.07 | C9H10O4 | 165.0552 | 165.0546 | -3.82 | 119.0488, 147.0447 | M+H-H2O | Methyl isovanillate | G |
| 137 | POS | 20.08 | C11H12O4 | 191.0709 | 191.0701 | -3.77 | 103.0550, 119.0855, 131.0488, 159.0446 | M+H-H2O | 3,4-Dimethoxycinnamic acid | G |
| 138 | POS | 20.09 | C21H22O10 | 435.1286 | 435.1276 | -2.21 |  | M+H | Isoengelitin | P |
| 139 | NEG | 20.17 | C36H36O18 | 755.1829 | 755.18 | -3.77 |  | M-H | Quercetin-3-*O*-[2-*O*-(6-*O*-p-hydroxyl-E-coumaroyl)-D-glucosyl]-(1-2)-L-rhamnoside | S |
| 140 | NEG | 20.2 | C27H30O14 | 559.1452 | 559.1455 | 0.57 |  | M-H2O-H | Chrysin 7-*O*-β-gentiobioside | S |
| 141 | NEG | 20.21 | C26H32O11 | 519.1872 | 519.1882 | 1.85 | 61.9875, 83.0128, 137.0606, 357.1368 | M-H | Matairesinoside | P, C |
| 142 | NEG | 20.22 | C15H10O4 | 299.0561 | 299.0565 | 1.4 |  | M+FA-H | 7,4'-Dihydroxyflavone | G, S |
| 143 | POS | 20.25 | C22H22O11 | 463.1235 | 463.1246 | 2.4 | 286.0453, 301.0695 | M+H | Diosmetin-7-*O*-β-D-glucopyranoside | S |
| 144 | NEG | 20.26 | C26H26O12 | 529.1352 | 529.1358 | 1.23 | 135.0443, 155.0348, 173.0437, 179.0353, 193.0515, 335.0768, 349.0952, 353.0882, 367.1024 | M-H | 4,5-Di-*O*-caffeoylquinic acid methyl ester | G, S |
| 145 | POS | 20.29 | C21H25NO5 | 354.1706 | 354.1696 | -2.85 |  | M+H-H2O | Corynoxidine | P |
| 146 | NEG | 20.32 | C26H30O15 | 581.1512 | 581.1529 | 2.89 |  | M-H | 1-*O*-[β-D-Xylopyranosyl-(1-6)-β-D-glucopyranosyl]-8-hydroxy-3,7-dimethoxyxanthone | G |
| 147 | POS | 20.35 | C19H16NO4+ | 322.1074 | 322.1067 | -1.99 |  | M+ | Berberrubine | P, C |
| 148 | POS | 20.37 | C19H21NO5 | 366.1312 | 366.1334 | 6.09 | 336.0864, 350.1008, 351.1087 | M+Na | *N*-trans-Sinapoyltyramine | C, G |
| 149 | NEG | 20.46 | C17H14O8 | 345.0616 | 345.0616 | 0.12 | 315.0134, 330.0366 | M-H | Viscidulin III | S |
| 150 | NEG | 20.47 | C27H34O11 | 533.2028 | 533.2039 | 2.06 |  | M-H | Phylliroside | P, C |
| 151 | NEG | 20.51 | C21H18O11 | 891.1626 | 891.1597 | -3.16 | 85.0284, 113.0240, 175.0243, 269.0456, 445.0754 | 2M-H | Baicalin | P, C, G, S |
| 152 | POS | 20.53 | C21H20O10 | 433.1129 | 433.1132 | 0.58 | 271.0612 | M+H | Oroxin A | S |
| 153 | POS | 20.55 | C15H10O6 | 287.055 | 287.0547 | -0.98 | 241.0516, 269.0461 | M+H | Isoscutellarein | S |
| 154 | POS | 20.58 | C20H18NO4+ | 336.123 | 336.1225 | -1.32 | 321.0997, 320.0920, 306.0763, 292.0970 | M-H | Berberine | C |
| 155 | NEG | 20.59 | C17H17NO3 | 282.1136 | 282.114 | 1.49 |  | M-H | p-Coumaroyltyramine | P |
| 156 | POS | 20.65 | C21H22NO4+ | 352.1543 | 352.1536 | -2.13 | 308.1273, 322.1071, 336.1257, 337.1334 | M+ | Palmatine | P, C, G, S |
| 157 | NEG | 20.66 | C22H20O11 | 505.0988 | 505.0998 | 1.98 |  | M+FA-H | Oroxylin A-7-*O*-glucuronide | S |
| 158 | NEG | 20.66 | C28H34O14 | 593.1876 | 593.1877 | 0.12 |  | M-H | Didymin | S |
| 159 | NEG | 20.7 | C32H40O17 | 695.2193 | 695.2170 | -3.25 | 101.0235, 117.0339, 119.0494, 123.0447, 145.0290, 163.0397, 225.0763, 227.6301, 469.1337, 695.2171 | M-H | 6′-O-p-Coumaroylgenipin gentiobioside | G |
| 160 | POS | 20.73 | C22H20O12 | 477.1028 | 477.1035 | 1.59 | 286.0452, 301.0695 | M+H | 5,8-Dihydroxy-6-methoxyflavone-7-*O*-glucuronide | S |
| 161 | NEG | 20.74 | C8H8O2 | 135.0452 | 135.0444 | -5.33 |  | M-H | Anisaldehyde | S, P, C |
| 162 | POS | 20.76 | C21H20O11 | 449.1078 | 449.109 | 2.54 | 169.0126, 271.0617, 272.0656, 273.0753 | M+H | Dihydrobaicalin | S |
| 163 | POS | 20.81 | C18H19NO4 | 314.1387 | 314.1378 | -2.67 | 121.0652, 145.0276, 177.0550, 186.0556 | M+H | N-Feruloyltyramine | C |
| 164 | POS | 20.85 | C15H10O6 | 269.045 | 269.0447 | -1.3 |  | M+H-H2O | Luteolin | S |
| 165 | NEG | 20.86 | C15H10O5 | 269.0456 | 269.0457 | 0.41 |  | M-H | 3,7,4'-Trihydroxyflavone | S |
| 166 | NEG | 20.88 | C24H22O13 | 517.0988 | 517.0988 | 0.12 |  | M-H | Apigenin-7-*O*-6''-Malonylglucoside | S |
| 167 | POS | 20.89 | C20H19NO4 | 338.1387 | 338.1389 | 0.62 | 227.6225, 307.0841, 308.0914, 322.1084, 323.1153, 338.1395 | M+H | Tetrahydrocorysamine | C |
| 168 | NEG | 20.9 | C31H40O15 | 651.2294 | 651.2298 | 0.6 | 113.0240, 134.0368, 160.0166, 175.0392, 193.0503 | M-H | Isomartynoside | S, G |
| 169 | NEG | 21 | C15H12O6 | 287.0561 | 287.0559 | -0.63 | 125.0237, 135.0447, 161.0244, 171.0449, 199.0763, 227.6311, 243.0666, 287.0457, 287.0559 | M-H | Eriodictyol | S |
| 170 | POS | 21.04 | C21H20NO4+ | 350.1387 | 350.139 | 0.91 |  | M+ | 13-Methylberberine | G, C |
| 171 | POS | 21.04 | C22H20O11 | 461.1078 | 461.1071 | -1.52 | 270.0534, 285.0767 | M+H | Wogonoside | S, C |
| 172 | NEG | 21.05 | C15H10O4 | 253.0506 | 253.0501 | -1.98 |  | M-H | 7,8-Dihydroxyflavone | S |
| 173 | POS | 21.06 | C9H8O2 | 131.0497 | 131.0493 | -3.59 | 103.0548 | M+H-H2O | Cinnamic acid | G |
| 174 | NEG | 21.06 | C21H18O10 | 429.0827 | 429.0831 | 0.96 | 59.0128, 85.0284, 99.0080, 113.0227, 175.0243, 253.0502 | M-H | Chrysin-7-*O*-glucuronide | S |
| 175 | NEG | 21.12 | C15H10O7 | 301.0354 | 301.0359 | 1.59 | 59.0128, 151.0033, 164.9269, 178.9982, 214.2307 | M-H | Quercetin | G, S, P, C |
| 176 | POS | 21.14 | C10H10O3 | 196.0968 | 196.0968 | -0.31 |  | M+NH4 | 4-Methoxycinnamic acid | G |
| 177 | POS | 21.14 | C9H10O2 | 168.1019 | 168.1018 | -0.77 |  | M+NH4 | 4-Vinylguaiacol | P |
| 178 | POS | 21.18 | C9H10O4 | 183.0652 | 183.0654 | 1.37 |  | M+H | 3,5-Dimethoxybenzoic acid | P |
| 179 | POS | 21.19 | C22H26O8 | 401.1601 | 401.1594 | -1.57 | 167.0695, 217.0859, 247.0975, 315.0880, 330.1094, 331.1156, 343.1194, 351.1234, 383.1474 | M+H-H2O | Syringaresinol | P, C, G, S |
| 180 | POS | 21.26 | C16H12O7 | 317.0656 | 317.0663 | 2.3 | 302.0423 | M+H | 3-*O*-Methylquercetin | S |
| 181 | NEG | 21.3 | C22H24O10 | 447.1297 | 447.1294 | -0.6 |  | M-H | Isosakuranin | S |
| 182 | POS | 21.39 | C23H22O12 | 491.1184 | 491.1187 | 0.61 | 285.0404, 300.0626, 315.0880 | M+H | 5,7-Dihydroxy-6,8-dimethoxyflavone-7-*O*-glucuronide | S |
| 183 | POS | 21.41 | C20H22O6 | 341.1389 | 341.1388 | -0.41 |  | M+H-H2O | Pinoresinol | C |
| 184 | POS | 21.41 | C15H10O5 | 271.0601 | 271.0602 | 0.26 | 271.0616 | M+H | Apigenin | G, S |
| 185 | NEG | 21.63 | C19H18O8 | 373.0929 | 373.0935 | 1.61 |  | M-H | Methyl rosmarinic acid | G |
| 186 | POS | 21.7 | C22H22O10 | 447.1286 | 447.1287 | 0.27 | 227.6253, 270.0517, 271.0600, 285.0759 | M+H | Wogonin 5-O-β-D-glucopyranoside | S |
| 187 | NEG | 21.9 | C16H14O6 | 301.0718 | 301.0724 | 2.09 |  | M-H | Homoeriodictyol | S |
| 188 | NEG | 21.92 | C15H12O5 | 317.0667 | 317.0663 | -1.26 |  | M+FA-H | Naringenin chalcone | S |
| 189 | POS | 22.08 | C17H18O5 | 320.1493 | 320.1499 | 2.09 |  | M+NH4 | 4,4'-Dihydroxy-2,6-dimethoxydihydrochalcone | G |
| 190 | NEG | 22.09 | C18H16O8 | 359.0772 | 359.0779 | 1.7 | 194.9944, 314.0076, 329.0282, 344.0544 | M-H | 6-Methoxytricin | P, S |
| 191 | POS | 22.23 | C16H12O6 | 301.0707 | 301.0702 | -1.49 | 286.0457 | M+H | Chrysoeriol | S |
| 192 | POS | 22.3 | C17H14O7 | 331.0812 | 331.0819 | 1.90 | 197.0445, 227.6253, 270.0517, 287.0550, 298.0480, 315.0494, 316.0586, 331.0808 | M+H | 5,7,2′-Trihydroxy-8,6′-dimethoxyflavone | S |
| 193 | POS | 22.38 | C16H14O4 | 271.0965 | 271.0958 | -2.66 |  | M+H | Alpinetin | S, G |
| 194 | POS | 22.4 | C15H10O5 | 271.0601 | 271.0595 | -2.1 |  | M+H | Norwogonin | S |
| 195 | NEG | 22.55 | C15H12O5 | 271.0612 | 271.061 | -0.92 |  | M-H | 2-Hydroxypinocembrin | S |
| 196 | POS | 22.59 | C17H14O7 | 331.0812 | 331.0813 | 0.09 | 169.0126, 316.0574 | M+H | Jaceosidin | S |
| 197 | POS | 22.71 | C15H10O5 | 271.0601 | 271.0596 | -1.92 |  | M+H | Baicalein | G, S, P, C |
| 198 | POS | 22.75 | C23H22O11 | 475.1235 | 475.1239 | 0.86 | 270.0536, 285.0770 | M+H | Oroxylin A 7-*O*-β-D-glucuronide methyl ester | S |
| 199 | POS | 22.85 | C16H12O6 | 301.0707 | 301.0707 | 0.07 |  | M+H | 5,7,2′-Trihydroxy-6-methoxyflavone | S |
| 200 | NEG | 23.16 | C15H10O4 | 299.0561 | 299.0567 | 1.97 |  | M+FA-H | 3,4'-Dihydroxyflavone | S |
| 201 | POS | 23.19 | C17H14O7 | 331.0812 | 331.0812 | -0.18 |  | M+H | Quercetin 3,4'-dimethyl ether | S |
| 202 | POS | 23.27 | C18H16O8 | 361.0918 | 361.0913 | -1.38 | 345.0587, 346.0653 | M+H | 5,7,3-Trihydroxy-6,4,5-trimethoxyflavone | S |
| 203 | POS | 23.53 | C20H20O8 | 389.1231 | 389.1226 | -1.23 | 359.0734 | M+H | 3'-Demethylnobiletin | S |
| 204 | POS | 23.56 | C16H12O6 | 283.0607 | 283.0602 | -1.63 |  | M+H-H2O | Hydroxygenkwanin | S |
| 205 | POS | 23.63 | C16H12O6 | 301.0707 | 301.0706 | -0.07 | 184.0003, 202.0106, 286.0474, 301.0709 | M+H | Scutevulin | S |
| 206 | POS | 24.33 | C18H16O5 | 313.1071 | 313.1066 | -1.37 |  | M+H | 5,7,8-Trimethoxyflavone | S |
| 207 | NEG | 24.4 | C32H44O14 | 651.2658 | 651.2661 | 0.4 | 59.0130, 71.0127, 89.0238, 101.0232, 179.0548, 213.8002, 239.1803, 283.1696, 327.1584 | M-H | Crocin IV | G |
| 208 | POS | 24.42 | C16H14O6 | 303.0863 | 303.0866 | 0.82 |  | M+H | 5,7,4′-Trihydroxy-6-methoxyflavanone | G, S |
| 209 | POS | 24.75 | C20H17NO5 | 352.118 | 352.118 | 0.17 | 308.0929, 308.1278, 324.1212, 336.1264, 337.1339 | M+H | 8-Oxoepiberberine | P, C |
| 210 | POS | 25.04 | C26H30O8 | 471.2013 | 471.2019 | 1.15 | 95.0129, 161.0595, 367.1925, 409.1985, 425.1925, 427.2138 | M+H | Limonin | P, C |
| 211 | POS | 25.48 | C18H16O7 | 345.0969 | 345.097 | 0.43 |  | M+H | Eupatorin | G, S |
| 212 | NEG | 25.91 | C16H12O5 | 283.0612 | 283.0612 | 0.07 | 268.0395 | M-H | Wogonin | P, C, G, S |
| 213 | NEG | 25.99 | C15H10O4 | 253.0506 | 253.0506 | -0.04 |  | M-H | 4',5-Dihydroxyflavone | S |
| 214 | NEG | 26.72 | C16H12O5 | 283.0612 | 283.0613 | 4.38 | 268.0378 | M-H | Oroxylin A | S |
| 215 | POS | 26.73 | C19H13NO5 | 358.0686 | 358.0684 | -0.42 |  | M+Na | 8-Oxocoptisine | C |
| 216 | NEG | 26.84 | C15H10O5 | 269.0456 | 269.046 | 1.78 |  | M-H | Galangin | C |
| 217 | POS | 26.88 | C16H14O5 | 287.0914 | 287.0913 | -0.31 |  | M+H | Isosakuranetin | S |
| 218 | NEG | 26.92 | C17H14O6 | 313.0718 | 313.0719 | 0.35 | 283.0265, 298.0488 | M-H | Skullcapflavone I | S |
| 219 | POS | 26.98 | C20H17NO5 | 352.118 | 352.1181 | 0.34 | 322.0701, 336.1261, 337.0932 | M+H | Oxyberberine | P, C |
| 220 | POS | 27.78 | C18H16O7 | 345.0969 | 345.097 | 0.38 | 297.0397, 315.0523 | M+H | 5,7-Dihydroxy-3,4',8-trimethoxyflavone | S |
| 221 | POS | 28.65 | C26H30O7 | 455.2064 | 455.2069 | 1.12 | 161.0594, 409.1979, 411.2157, 437.1948 | M+H | Obacunone | P, C |
| 222 | POS | 29.05 | C18H16O7 | 345.0969 | 345.0967 | -0.67 | 197.0079, 227.6250, 297.0392, 312.0634, 315.0494, 330.0727, 345.0973 | M+H | Tenaxin I | S |
| 223 | NEG | 29.93 | C16H14O2 | 283.0976 | 283.0982 | 2.15 |  | M+FA-H | 4'-Methoxychalcone | G |
| 224 | POS | 30.08 | C19H18O7 | 359.1125 | 359.1125 | 0 |  | M+H | Gardenin B | G, S |
| 225 | NEG | 30.13 | C17H14O5 | 297.0769 | 297.0777 | 2.86 |  | M-H | 7,4'-Di-*O*-methylapigenin | S |
| 226 | NEG | 30.43 | C18H36O4 | 315.2541 | 315.2544 | 0.95 |  | M-H | 9,10-Dihydroxystearic acid | P |
| 227 | POS | 30.46 | C17H14O6 | 297.0763 | 297.0761 | -0.91 |  | M+H-H2O | 3,5-Dihydroxy-4',7-dimethoxyflavone | S |
| 228 | NEG | 33.95 | C16H32O3 | 271.2279 | 271.2282 | 1.25 | 225.2223 | M-H | 2-Hydroxypalmitic acid | P |
| 229 | NEG | 34.37 | C30H48O3 | 455.3531 | 455.353 | -0.26 |  | M-H | Oleanolic acid | G, P |
| 230 | POS | 34.49 | C30H48O3 | 439.3577 | 439.3575 | -0.27 | 95.0863, 184.8900, 189.1643, 191.1796, 203.1799, 205.1971, 214.6318, 239.1503, 249.1862 | M+H-H2O | Ursolic acid | P, G |

S: Scutellaria baicalensis, C: Coptis chinensis, P: Phellodendron amurense, and G: Gardenia jasminoides

**Table S4. Putatively identified prototype compounds and metabolites of HLJDD in mouse plasma, liver, and brain samples collected 2 h after a single oral administration.**

| **No.** | **Compound** | **Type** | **Molecular formula** | **Ion mode** | **Adduct** | **RT (min)** | **Theoretical *m/z*** | **Measured *m/z*** | **Mass error (ppm)** | **Fragment Ions m/z** | **Plasma** | **Liver** | **Brain** |
| --- | --- | --- | --- | --- | --- | --- | --- | --- | --- | --- | --- | --- | --- |
| 1 | Geniposidic acid | Prototype | C_16_H_22_O_10_ | POS | M+Na | 9.02 | 397.1105 | 397.1106 | 0.15 | 227.6249, 397.1101 | Y | Y | N |
| 2 | Gardenoside | Prototype | C_17_H_24_O_11_ | POS | M+Na | 9.55 | 427.1211 | 427.1211 | -0.02 | 227.6234, 265.0686, 427.1216 | Y | Y | Y |
| 3 | Feretoside | Prototype | C_17_H_24_O_11_ | POS | M+Na | 10.04 | 427.1211 | 427.121 | -0.09 | 427.1216 | Y | N | N |
| 4 | Genipinic acid | Prototype | C_11_H_14_O_6_ | POS | M+H-H_2_O | 10.04 | 225.0763 | 225.0757 | -2.8 | 67.0542, 91.0543, 95.0491, 119.0492, 123.0440, 147.0441, 165.0545, 175.0392, 189.0548, 193.0496 | Y | N | N |
| 5 | Jasminoside | Prototype | C_16_H_26_O_8_ | POS | M+Na | 10.28 | 369.152 | 369.1519 | -0.35 | 227.6267, 369.1520 | Y | Y | Y |
| 6 | 4-(Hydroxymethyl)-2,6-dimethoxyphenol | Prototype | C_9_H_12_O_4_ | POS | M+H | 12.7 | 185.0808 | 185.0808 | -0.11 | 121.1012, 125.0596, 125.0961, 137.0961, 139.1115, 153.0545, 154.0625, 167.1066, 185.0806, 185.1169 | Y | Y | N |
| 7 | Gardoside methyl ester | Prototype | C_17_H_24_O_10_ | NEG | M+FA-H | 14.15 | 433.1352 | 433.1339 | -2.86 | 101.0236, 123.0448, 147.0448, 207.0660, 225.0765, 227.6272 | Y | Y | Y |
| 8 | Geniposide | Prototype | C17H24O10 | POS | M+Na | 14.15 | 411.1262 | 411.1268 | 1.46 |  | Y | Y | Y |
| 9 | Magnoflorine | Prototype | C20H24NO4+ | POS | M+ | 14.89 | 342.17 | 342.1691 | -2.57 |  | Y | Y | N |
| 10 | Norisocorydine | Prototype | C_19_H_21_NO_4_ | POS | M+H | 16.35 | 328.1543 | 328.1547 | 1.04 | 58.0651, 121.0284, 205.0649, 222.0679, 227.6224, 233.0595, 237.0907, 265.0853, 297.1125, 328.1548 | N | Y | N |
| 11 | 2-Hydroxycinnamic acid | Prototype | C_9_H_8_O_3_ | POS | M+H-H_2_O | 18.59 | 147.0446 | 147.0441 | -3.74 | 91.0542, 119.0491, 147.0441 | Y | N | N |
| 12 | Luteolin 7-*O*-β-D-glucopyranoside | Prototype | C_21_H_20_O_12_ | NEG | M-H | 18.65 | 463.0882 | 463.0866 | -3.52 | 151.0033, 227.6284, 243.0300, 255.0293, 271.0249, 300.0274, 301.0344, 463.0880 | Y | Y | N |
| 13 | Thalifendine | Prototype | C_19_H_16_NO_4_^+^ | POS | M+ | 19.41 | 322.1074 | 322.1071 | -0.93 | 227.6256, 279.0890, 307.0842, 322.1066 | Y | Y | N |
| 14 | Chrysin 6-*C*-arabinoside 8-*C*-glucoside | Prototype | C_26_H_28_O_13_ | POS | M+H | 19.52 | 549.1603 | 549.1597 | -0.97 | 227.6277, 279.0651, 291.0649, 309.0755, 321.0752, 333.0766, 363.0866, 381.0963, 495.1292, 513.1397 | Y | Y | N |
| 15 | Oroxin A | Prototype | C_21_H_20_O_10_ | NEG | M+FA-H | 20.7 | 477.1039 | 477.1032 | -1.43 | 110.0002, 113.0238, 165.9904, 166.9982, 175.0249, 227.6270, 227.6342, 286.0485, 287.0560, 301.0704 | Y | Y | N |
| 16 | Berberrubine | Prototype | C_19_H_16_NO_4_^+^ | POS | M+ | 20.75 | 322.1074 | 322.1074 | -0.03 | 279.0887, 307.0840, 322.1064 | Y | Y | Y |
| 17 | Tetrahydrocorysamine | Prototype | C_20_H_19_NO_4_ | POS | M+H | 20.89 | 338.1387 | 338.1389 | 0.62 | 227.6225, 307.0841, 308.0914, 322.1084, 323.1153, 338.1395 | Y | Y | Y |
| 18 | Eriodictyol | Prototype | C_15_H_12_O_6_ | NEG | M-H | 21 | 287.0561 | 287.0559 | -0.63 | 125.0237, 135.0447, 161.0244, 171.0449, 199.0763, 227.6311, 243.0666, 287.0457, 287.0559 | Y | Y | N |
| 19 | Oroxylin A-7-*O*_-_glucuronide | Prototype | C22H20O11 | NEG | M+FA-H | 21.23 | 505.0988 | 505.0998 | 1.98 |  | Y | Y | N |
| 20 | Berberine | Prototype | C20H18NO4 | POS | M+ | 21.51 | 336.123 | 336.1225 | -1.32 | 321.0997, 320.0920, 306.0763, 292.0970 | Y | Y | N |
| 21 | Wogonoside | Prototype | C22H20O11 | POS | M+H | 21.69 | 461.1078 | 461.1071 | -1.52 | 270.0534, 285.0767 | Y | Y | N |
| 22 | Wogonin 5-*O*-β-D-glucopyranoside | Prototype | C_22_H_22_O_10_ | POS | M+H | 21.7 | 447.1286 | 447.1287 | 0.27 | 227.6253, 270.0517, 271.0600, 285.0759 | Y | Y | N |
| 23 | Baicalin | Prototype | C21H18O11 | NEG | 2M-H | 21.89 | 891.1626 | 891.1597 | -3.16 | 85.0284, 113.0240, 175.0243, 269.0456, 445.0754 | Y | N | N |
| 24 | Oroxylin A 7-*O*-glucuronide | Prototype | C_22_H_20_O_11_ | POS | M+H | 22 | 461.1078 | 461.1077 | -0.35 | 270.0529, 285.0758 | Y | Y | Y |
| 25 | 5,7,2′-Trihydroxy-8,6′-dimethoxyflavone | Prototype | C_17_H_14_O_7_ | POS | M+H | 22.3 | 331.0812 | 331.0819 | 1.9 | 197.0445, 227.6253, 270.0517, 287.0550, 298.0480, 315.0494, 316.0586, 331.0808 | Y | Y | N |
| 26 | 5,7,2′-Trihydroxy-6-methoxyflavone | Prototype | C_16_H_12_O_6_ | POS | M+H | 22.85 | 301.0707 | 301.0707 | 0.07 |  | Y | Y | N |
| 27 | Scutevulin | Prototype | C_16_H_12_O_6_ | POS | M+H | 23.63 | 301.0707 | 301.0706 | -0.07 | 184.0003, 202.0106, 286.0474, 301.0709 | Y | Y | N |
| 28 | 5,7,4′-Trihydroxy-6-methoxyflavanone | Prototype | C_16_H_14_O_6_ | POS | M+H | 24.42 | 303.0863 | 303.0866 | 0.82 |  | N | Y | N |
| 29 | Wogonin | Prototype | C16H12O5 | NEG | M-H | 27.48 | 283.0612 | 283.0612 | 0.07 | 268.0395 | Y | Y | N |
| 30 | Oroxylin A | Prototype | C16H12O5 | NEG | M-H | 28.28 | 283.0612 | 283.0613 | 0.35 | 268.0378 | Y | Y | N |
| 31 | Tenaxin I | Prototype | C_18_H_16_O_7_ | POS | M+H | 29.05 | 345.0969 | 345.0967 | -0.67 | 197.0079, 227.6250, 297.0392, 312.0634, 315.0494, 330.0727, 345.0973 | N | Y | N |
| 32 | Geniposide sulfate | Metabolite | C_17_H_24_O_13_S | POS | M+H | 9.6 | 469.101 | 469.1057 | 10 | 104.0340, 130.0498, 162.0220, 164.0373, 177.0327, 193.9943, 227.6271, 237.0366, 340.0631, 394.0729 | N | Y | N |
| 33 | Phenolic sulfate conjugate | Metabolite | C_9_H_10_O_7_S | NEG | M-H | 10.52 | 261.0075 | 261.0075 | 0.11 | 121.0289, 135.0448, 137.0603, 181.0503, 227.6303, 261.0079 | Y | N | N |
| 34 | Ferulic acid sulfate | Metabolite | C_10_H_10_O_7_S | NEG | M-H | 12.63 | 273.0075 | 273.0073 | -0.55 | 134.0367, 149.0605, 178.0272, 193.0502, 227.6286, 273.0066 | Y | Y | Y |
| 35 | Pseudoprotopine-related metabolite | Metabolite | C_25_H_27_NO_11_ | POS | M+H | 13.41 | 518.1657 | 518.166 | 0.58 |  | Y | Y | N |

Y and N indicate detected and not detected, respectively, in the corresponding biological matrix. Prototype denotes an unchanged constituent derived from Huang-Lian-Jie-Du Decoction, whereas metabolite denotes a putative biotransformation product.

**Table S5. Comparisons of body weight between groups at each time point.**

| Week | Comparison | n (AD) | n (comparison group) | Mean difference (g) | 95% CI of difference (g) | adjusted P value |
| --- | --- | --- | --- | --- | --- | --- |
| 0W | AD vs WT | 16 | 15 | -1.278 | −3.0910 to 0.5359 | 0.2326 |
| 0W | AD vs H-H | 16 | 16 | 0.2358 | −1.4450 to 1.9170 | 0.9896 |
| 0W | AD vs H-L | 16 | 11 | -0.983 | −3.1110 to 1.1450 | 0.5687 |
| 0W | AD vs Don | 16 | 10 | 0.8325 | −0.6899 to 2.3550 | 0.439 |
| 1W | AD vs WT | 16 | 15 | -1.152 | −3.0630 to 0.7592 | 0.3565 |
| 1W | AD vs H-H | 16 | 16 | 0.8283 | −0.9215 to 2.5780 | 0.5696 |
| 1W | AD vs H-L | 16 | 11 | 0.02955 | −1.7440 to 1.8040 | >0.9999 |
| 1W | AD vs Don | 16 | 10 | 0.365 | −0.9030 to 1.6330 | 0.8721 |
| 2W | AD vs WT | 16 | 15 | -1.133 | −2.9410 to 0.6757 | 0.3243 |
| 2W | AD vs H-H | 16 | 16 | 2.254 | 0.5373 to 3.9700 | 0.0073 |
| 2W | AD vs H-L | 16 | 11 | -0.07898 | −2.0630 to 1.9050 | 0.9999 |
| 2W | AD vs Don | 16 | 10 | 0.6837 | −0.6503 to 2.0180 | 0.4965 |
| 3W | AD vs WT | 16 | 15 | -1.978 | −3.9420 to −0.0150 | 0.0479 |
| 3W | AD vs H-H | 16 | 16 | 1.835 | 0.0779 to 3.5920 | 0.0386 |
| 3W | AD vs H-L | 16 | 11 | 0.7841 | −1.1600 to 2.7290 | 0.6775 |
| 3W | AD vs Don | 16 | 10 | 0.455 | −0.9492 to 1.8590 | 0.8217 |
| 4W | AD vs WT | 16 | 15 | -2.026 | −3.9010 to −0.1513 | 0.0312 |
| 4W | AD vs H-H | 16 | 16 | 1.827 | 0.0067 to 3.6470 | 0.0489 |
| 4W | AD vs H-L | 16 | 11 | 0.5756 | −1.4850 to 2.6360 | 0.8747 |
| 4W | AD vs Don | 16 | 10 | 0.4337 | −0.9462 to 1.8140 | 0.8363 |
| 5W | AD vs WT | 16 | 15 | -1.823 | −3.4940 to −0.1528 | 0.0291 |
| 5W | AD vs H-H | 16 | 16 | 1.337 | −0.3089 to 2.9820 | 0.1387 |
| 5W | AD vs H-L | 16 | 11 | 0.5227 | −1.5740 to 2.6190 | 0.91 |
| 5W | AD vs Don | 16 | 10 | 0.34 | −1.1950 to 1.8750 | 0.9432 |
| 6W | AD vs WT | 16 | 15 | -1.855 | −3.7090 to −0.0007 | 0.0499 |
| 6W | AD vs H-H | 16 | 16 | 1.125 | −0.4818 to 2.7320 | 0.2386 |
| 6W | AD vs H-L | 16 | 11 | 0.4614 | −1.8410 to 2.7640 | 0.9551 |
| 6W | AD vs Don | 16 | 10 | 0.765 | −0.7455 to 2.2760 | 0.5056 |
| 7W | AD vs WT | 16 | 15 | -2.059 | −3.8150 to −0.3030 | 0.0177 |
| 7W | AD vs H-H | 16 | 16 | 0.9075 | −0.6209 to 2.4360 | 0.3749 |
| 7W | AD vs H-L | 16 | 11 | 0.06932 | −2.2140 to 2.3530 | 0.9999 |
| 7W | AD vs Don | 16 | 10 | 0.6875 | −0.7529 to 2.1280 | 0.5571 |
| 8W | AD vs WT | 16 | 15 | -2.084 | −3.8150 to −0.3536 | 0.0144 |
| 8W | AD vs H-H | 16 | 16 | 0.9025 | −0.6510 to 2.4560 | 0.3941 |
| 8W | AD vs H-L | 16 | 11 | 0.02614 | −2.3710 to 2.4230 | >0.9999 |
| 8W | AD vs Don | 16 | 10 | 0.5425 | −0.9442 to 2.0290 | 0.7559 |
| 9W | AD vs WT | 16 | 15 | -2.247 | −3.9650 to −0.5288 | 0.0074 |
| 9W | AD vs H-H | 16 | 16 | 0.7867 | −0.8402 to 2.4140 | 0.5548 |
| 9W | AD vs H-L | 16 | 11 | 0.2545 | −2.0270 to 2.5360 | 0.9947 |
| 9W | AD vs Don | 16 | 10 | 0.78 | −0.5992 to 2.1590 | 0.4101 |
| 10W | AD vs WT | 16 | 15 | -1.997 | −3.7350 to −0.2579 | 0.0206 |
| 10W | AD vs H-H | 16 | 16 | 0.85 | −0.7219 to 2.4220 | 0.4573 |
| 10W | AD vs H-L | 16 | 11 | -0.1591 | −2.6550 to 2.3370 | 0.9991 |
| 10W | AD vs Don | 16 | 10 | 0.77 | −0.4793 to 2.0190 | 0.3324 |

**Table S6. The visceral-body ratio of mice in each group after drug administration.**

| Visceral-body ratio (%) | WT | AD | H-H | H-L | Don |
| --- | --- | --- | --- | --- | --- |
| Liver/body ratio | 4.46 ± 0.14 | 3.81 ± 0.14 | 4.02 ± 0.05 | 4.09 ± 0.10 | 4.33 ± 0.18 |
| Brain/body ratio | 1.40 ± 0.02 | 1.55 ± 0.02 | 1.58 ± 0.02 | 1.52 ± 0.03 | 1.55 ± 0.02 |
| Spleen/body ratio | 0.35 ± 0.02 | 0.25 ± 0.01 | 0.26 ± 0.01 | 0.25 ± 0.01 | 0.33 ± 0.05 |

Data are presented as mean ± SEM and expressed as percentages of body weight

**Table S7. Differential metabolites in brain tissues of 5xFAD mice (HLJDD group vs AD group).**

| Identification | Formula | Reference ion | Measured [*m/z*] | Calculated [*m/z*] | Delta Mass [ppm] | RT [min] | Mode | Log2 FC: (HLJDD) / (AD) | P-value: (HLJDD) / (AD) | VIP: (HLJDD) / (AD) |
| --- | --- | --- | --- | --- | --- | --- | --- | --- | --- | --- |
| 3-(3'-Methylthio)propylmalic acid | C_8_H_14_O_5_S | [M+H]^+^ | 223.0630 | 222.0557 | -2.17 | 1.510 | HILIC | -0.69 | 0.034927 | 2.35671 |
| Succinic acid | C_4_H_6_O_4_ | [M-H]^-^ | 117.0190 | 118.0262 | -3.17 | 5.978 | HILIC | 0.70 | 0.003931 | 1.14397 |
| Butane-2,3-dione | C_4_H_6_O_2_ | [M-H]^-^ | 85.0294 | 86.0367 | -0.77 | 8.041 | HILIC | 0.39 | 0.010581 | 1.12215 |
| Cyclohexylamine | C_6_H_13_N | [M+H]^+^ | 100.1119 | 99.1046 | -1.90 | 9.338 | HILIC | -7.31 | 1.41E-07 | 3.85681 |
| Hypoxanthine | C_5_H_4_N_4_O | [M+H]^+^ | 137.0455 | 136.0382 | -2.18 | 10.763 | HILIC | 0.49 | 0.020663 | 1.62393 |
| Urocanic acid | C_6_H_5_N_2_O_2_ | [M+H]^+^ | 138.0425 | 137.0352 | 0.50 | 10.773 | HILIC | 0.61 | 0.012728 | 1.65821 |
| L-Prolyl-L-leucine | C_11_H_20_N_2_O_3_ | [M+H]^+^ | 229.1541 | 228.1468 | -2.44 | 10.812 | HILIC | 2.86 | 0.02762 | 1.71281 |
| Adenosine monophosphate | C_10_H_14_N_5_O_7_P | [M-H]^-^ | 347.0574 | 348.0647 | -2.77 | 17.261 | HILIC | -0.62 | 0.004923 | 1.54142 |
| 4-Hydroxy-L-threonine | C_4_H_9_NO_4_ | [M+H]^+^ | 136.0617 | 135.0544 | 9.00 | 1.091 | RP-C18 | 1.88 | 0.026741 | 2.03167 |
| 4-Trimethylammoniobutanoic acid | C_7_H_15_NO_2_ | [M+H]^+^ | 146.1175 | 145.1102 | -0.35 | 1.102 | RP-C18 | 1.51 | 0.038087 | 2.079 |
| Indole | C_8_H_7_N | [M+H]^+^ | 118.0648 | 117.0575 | -2.77 | 5.714 | RP-C18 | -0.27 | 0.012859 | 1.33718 |
| Styrene | C_8_H_8_ | [M+H]^+^ | 105.0695 | 104.0622 | -3.46 | 5.715 | RP-C18 | -0.30 | 0.01779 | 1.2981 |
| Palmitamide | C_16_H_33_NO | [M+H]^+^ | 256.2629 | 255.2557 | -2.20 | 6.073 | RP-C18 | -0.53 | 0.000268 | 1.50607 |
| Octadecanamide | C_18_H_37_NO | [M+H]^+^ | 284.2940 | 283.2867 | -2.85 | 7.114 | RP-C18 | -0.44 | 0.000606 | 1.37129 |
| (2E,4E)-N-Isobutyl-2,4-octadecadienamide | C_22_H_41_NO | [M+H]^+^ | 336.3248 | 335.3175 | -3.83 | 9.660 | RP-C18 | 0.45 | 0.014465 | 1.2985 |
| Eicosapentaenoic acid | C_20_H_30_O_2_ | [M-H]^-^ | 301.2154 | 302.2227 | -6.16 | 9.822 | RP-C18 | 0.87 | 6.11E-05 | 1.55593 |
| (9E)-N-(2-Hydroxyethyl)-9-octadecenamide | C_20_H_39_NO_2_ | [M+H]^+^ | 326.3041 | 325.2968 | -3.81 | 10.152 | RP-C18 | 0.59 | 0.027189 | 1.28877 |
| Docosahexaenoic acid | C_22_H_32_O_2_ | [M+H]^+^ | 329.2462 | 328.2390 | -3.85 | 10.533 | RP-C18 | 0.81 | 1.23E-05 | 1.86696 |
| Cinnamaldehyde | C_9_H_8_O | [M+H]^+^ | 133.0643 | 132.0570 | -3.96 | 10.919 | RP-C18 | -0.84 | 1.25E-06 | 1.62064 |
| Docosatetraenoic acid | C_22_H_36_O_2_ | [M-H]^-^ | 331.2629 | 332.2702 | -3.96 | 11.842 | RP-C18 | 0.97 | 0.001933 | 1.40666 |

**Table S8. Differential metabolites in liver tissues of 5xFAD mice (HLJDD group vs AD group).**

| Identification | Formula | Reference ion | Measured [*m/z*] | Calculated [*m/z*] | Delta Mass [ppm] | RT [min] | Mode | Log2 FC: (HLJDD) / (AD) | P-value: (HLJDD) / (AD) | VIP: (HLJDD) / (AD) |
| --- | --- | --- | --- | --- | --- | --- | --- | --- | --- | --- |
| p-Xylene | C_8_H_10_ | [M+H]^+^ | 107.0850 | 106.0777 | -4.81 | 1.796 | HILIC | 0.68 | 0.01223 | 1.01544 |
| Norepinephrine | C_8_H_11_NO_3_ | [M+H]^+^ | 170.0806 | 169.0733 | -3.63 | 2.329 | HILIC | -5.16 | 2.74E-09 | 1.65788 |
| Malonic acid | C_3_H_4_O_4_ | [M-H]^-^ | 103.0034 | 104.0107 | -2.81 | 3.551 | HILIC | -1.19 | 4.45E-05 | 1.60803 |
| Pyridine | C_5_H_5_N | [M+H]^+^ | 80.0490 | 79.0418 | -5.64 | 4.126 | HILIC | 2.53 | 0.000819 | 1.458 |
| Glycine | C_2_H_5_NO_2_ | [M-H]^-^ | 74.0246 | 75.0318 | -2.69 | 4.254 | HILIC | -0.53 | 0.035594 | 1.5235 |
| Sphinganine | C_18_H_39_NO_2_ | [M+H]^+^ | 302.3044 | 301.2971 | -3.23 | 4.586 | HILIC | 1.63 | 0.000295 | 1.34721 |
| Threose | C_4_H_8_O_4_ | [M-H]^-^ | 119.0346 | 120.0419 | -3.07 | 4.823 | HILIC | -1.03 | 0.001271 | 1.19707 |
| Valinol | C_5_H_13_NO | [M+H]^+^ | 104.1065 | 103.0993 | -4.50 | 4.920 | HILIC | -2.35 | 0.007406 | 1.14961 |
| Pantothenic acid | C_9_H_17_NO_5_ | [M+H]^+^ | 220.1173 | 219.1100 | -3.11 | 5.103 | HILIC | 0.78 | 0.00682 | 1.12378 |
| Zeatin | C_10_H_13_N_5_O | [M+H]^+^ | 220.1173 | 219.1100 | -9.25 | 5.250 | HILIC | 0.79 | 0.025608 | 1.50187 |
| Butane-2,3-dione | C_4_H_6_O_2_ | [M-H]^-^ | 85.0293 | 86.0365 | -2.90 | 6.396 | HILIC | -0.31 | 0.023641 | 1.38168 |
| N-Acetyl-L-glutamic acid | C_7_H_11_NO_5_ | [M+H]^+^ | 190.0702 | 189.0629 | -4.15 | 7.049 | HILIC | -0.80 | 0.000144 | 1.65504 |
| Malondialdehyde | C_3_H_4_O_2_ | [M-H]^-^ | 71.0136 | 72.0209 | -2.90 | 7.057 | HILIC | -0.29 | 0.01647 | 1.38809 |
| L-Ethionine | C_6_H_13_NO_2_S | [M+H]^+^ | 164.0733 | 163.0660 | -4.17 | 7.572 | HILIC | 3.43 | 0.002231 | 1.27982 |
| gamma-Aminobutyric acid | C_4_H_9_NO_2_ | [M+H]^+^ | 104.0702 | 103.0629 | -4.01 | 8.020 | HILIC | 5.24 | 0.00193 | 1.11378 |
| Oleamide | C_18_H_35_NO | [M+H]^+^ | 282.2777 | 281.2705 | -5.01 | 8.617 | HILIC | 2.20 | 2.44E-08 | 1.48114 |
| Threo-sphingosine | C_18_H_37_NO_2_ | [M+H]^+^ | 300.2881 | 299.2809 | -5.28 | 8.641 | HILIC | 2.96 | 3.26E-07 | 1.46027 |
| L-Alanine | C_3_H_7_NO_2_ | [M+H]^+^ | 90.0544 | 89.0471 | -6.23 | 9.044 | HILIC | 3.03 | 0.000121 | 1.29259 |
| Aceturic acid | C_4_H_7_NO_3_ | [M-H]^-^ | 116.0351 | 117.0424 | -1.57 | 9.234 | HILIC | -1.18 | 0.0142 | 1.23742 |
| 2-Oxoglutarate dianion | C_5_H_4_O_5_ | [M-H]^-^ | 142.9982 | 144.0054 | -3.02 | 9.500 | HILIC | -0.43 | 0.018105 | 1.29733 |
| L-Acetylcarnitine | C_9_H_17_NO_4_ | [M+H]^+^ | 204.1224 | 203.1151 | -3.34 | 9.594 | HILIC | -4.87 | 0.01198 | 1.33074 |
| Norhaman | C_11_H_8_N_2_ | [M+H]^+^ | 169.0753 | 168.0681 | -4.18 | 9.653 | HILIC | -1.18 | 0.026788 | 1.1335 |
| Pterin | C_6_H_5_N_5_O | [M+H]^+^ | 164.0561 | 163.0488 | -3.93 | 9.691 | HILIC | -0.53 | 0.004145 | 1.50362 |
| Allantoin | C_4_H_6_N_4_O_3_ | [M-H]^-^ | 157.0360 | 158.0433 | -4.60 | 10.147 | HILIC | -0.26 | 0.024083 | 1.25179 |
| Phosphocholine | C_5_H_14_NO_4_P | [M+H]^+^ | 184.0726 | 183.0653 | -4.23 | 10.280 | HILIC | 1.03 | 0.021006 | 1.19664 |
| Creatinine | C_4_H_7_N_3_O | [M+H]^+^ | 114.0656 | 113.0583 | -5.28 | 10.627 | HILIC | -1.46 | 0.000237 | 1.40569 |
| 4-Trimethylammoniobutanoic acid | C_7_H_15_NO_2_ | [M+H]^+^ | 146.1169 | 145.1096 | -4.81 | 10.628 | HILIC | -3.36 | 3.05E-06 | 1.67372 |
| Oleic acid | C_18_H_34_O_2_ | [M-H]^-^ | 281.2468 | 282.2541 | -6.46 | 10.692 | HILIC | 0.53 | 0.030627 | 1.38692 |
| Adenine | C_5_H_5_N_5_ | [M+H]^+^ | 136.0611 | 135.0538 | -4.86 | 10.865 | HILIC | -0.91 | 0.004141 | 1.36027 |
| N-Acetylgalactosamine 6-sulfate | C_8_H_15_NO_9_S | [M-H]^-^ | 300.0375 | 301.0447 | -6.72 | 10.869 | HILIC | -1.01 | 1.26E-06 | 1.66108 |
| L-Prolyl-L-leucine | C_11_H_20_N_2_O_3_ | [M+H]^+^ | 229.1537 | 228.1465 | -4.10 | 11.366 | HILIC | 3.49 | 5.55E-05 | 1.41527 |
| Hypoxanthine | C_5_H_4_N_4_O | [M+H]^+^ | 137.0452 | 136.0379 | -4.48 | 11.408 | HILIC | -1.20 | 0.024174 | 1.04122 |
| L-Carnitine | C_7_H_15_NO_3_ | [M+H]^+^ | 162.1119 | 161.1046 | -3.80 | 11.594 | HILIC | 9.04 | 2.15E-09 | 1.68154 |
| Neuraminic acid | C_9_H_17_NO_8_ | [M+H]^+^ | 268.1028 | 267.0955 | 0.30 | 11.632 | HILIC | -1.84 | 2.93E-07 | 1.81039 |
| Indole | C_8_H_7_N | [M+H]^+^ | 118.0646 | 117.0573 | -4.72 | 11.634 | HILIC | -0.88 | 0.000934 | 1.45599 |
| Aminoadipic acid | C_6_H_11_NO_4_ | [M+H]^+^ | 162.0754 | 161.0681 | -4.50 | 11.678 | HILIC | -2.01 | 0.000272 | 1.76319 |
| Pyrimidine | C_4_H_4_N_2_ | [M+H]^+^ | 81.0442 | 80.0369 | -6.93 | 11.682 | HILIC | -1.45 | 1.38E-09 | 1.68942 |
| (R)-3-hydroxybutyrylcarnitine | C_11_H_21_NO_5_ | [M+H]^+^ | 248.1482 | 247.1409 | -4.46 | 11.686 | HILIC | -9.04 | 6.87E-08 | 1.76508 |
| Proline | C_5_H_9_NO_2_ | [M+H]^+^ | 116.0701 | 115.0628 | -4.73 | 11.769 | HILIC | -0.89 | 0.001573 | 1.52345 |
| Acetamide | C_2_H_5_NO | [M+H]^+^ | 60.0438 | 59.0366 | -9.26 | 11.779 | HILIC | -0.70 | 0.013073 | 1.24069 |
| L-Threonine | C_4_H_9_NO_3_ | [M+H]^+^ | 120.0650 | 119.0577 | -4.71 | 11.780 | HILIC | -5.14 | 9.44E-07 | 1.61499 |
| Isoxanthopterin | C_6_H_5_N_5_O_2_ | [M-H]^-^ | 178.0360 | 179.0433 | -5.98 | 11.982 | HILIC | 2.15 | 0.017699 | 1.30072 |
| 3-Sulfinoalanine | C_3_H_7_NO_4_S | [M-H]^-^ | 152.0015 | 153.0088 | -5.10 | 12.074 | HILIC | -1.64 | 8.49E-06 | 1.65366 |
| gamma-Glutamylleucine | C_11_H_20_N_2_O_5_ | [M+H]^+^ | 261.1435 | 260.1363 | -3.71 | 12.146 | HILIC | 4.22 | 3.57E-07 | 1.75319 |
| Creatine | C_4_H_9_N_3_O_2_ | [M+H]^+^ | 132.0764 | 131.0691 | -2.93 | 12.153 | HILIC | 2.94 | 2.09E-07 | 1.6363 |
| Isoprene | C_5_H_8_ | [M+H]^+^ | 69.0694 | 68.0621 | -7.45 | 12.302 | HILIC | 0.49 | 3.89E-05 | 1.66435 |
| Nicotinamide | C_6_H_6_N_2_O | [M+H]^+^ | 123.0548 | 122.0475 | -4.29 | 12.353 | HILIC | 1.02 | 2.41E-09 | 1.68504 |
| Taurine | C_2_H_7_NO_3_S | [M-H]^-^ | 124.0068 | 125.0141 | -4.52 | 12.370 | HILIC | -1.88 | 1.86E-05 | 1.73535 |
| Glycyl-Lysine | C_8_H_17_N_3_O_3_ | [M+H]^+^ | 204.1334 | 203.1261 | -4.23 | 12.482 | HILIC | -1.93 | 6.16E-05 | 1.67801 |
| Alanylproline | C_8_H_14_N_2_O_3_ | [M+H]^+^ | 187.1069 | 186.0996 | -4.55 | 12.494 | HILIC | 3.00 | 1.83E-07 | 1.87055 |
| Tranexamic acid | C_8_H_15_NO_2_ | [M+H]^+^ | 158.1169 | 157.1095 | -4.75 | 12.537 | HILIC | 1.39 | 2.54E-05 | 1.53126 |
| Glycylleucine | C_8_H_16_N_2_O_3_ | [M-H]^-^ | 187.1075 | 188.1148 | -6.81 | 12.573 | HILIC | -5.24 | 1.19E-06 | 1.6103 |
| Leucine | C_6_H_13_NO_2_ | [M-H]^-^ | 130.0867 | 131.0940 | -5.17 | 12.672 | HILIC | -2.82 | 4.8E-06 | 1.55487 |
| N-Methylethanolamine phosphate | C_3_H_10_NO_4_P | [M+H]^+^ | 156.0416 | 155.0343 | -3.06 | 12.692 | HILIC | -1.16 | 3.15E-06 | 1.61408 |
| Guanine | C_5_H_5_N_5_O | [M+H]^+^ | 152.0561 | 151.0488 | -3.91 | 12.708 | HILIC | 1.51 | 0.001301 | 1.41006 |
| Desthiobiotin | C_10_H_18_N_2_O_3_ | [M+H]^+^ | 215.1381 | 214.1309 | -4.10 | 12.894 | HILIC | -2.76 | 0.001721 | 1.5323 |
| 5-Methylcytosine | C_5_H_7_N_3_O | [M+H]^+^ | 126.0657 | 125.0584 | -4.17 | 12.917 | HILIC | 0.80 | 0.002059 | 1.35507 |
| Threonic acid | C_4_H_8_O_5_ | [M+H]^+^ | 137.0452 | 136.0379 | 5.57 | 12.923 | HILIC | 0.34 | 0.022705 | 1.464 |
| Cytidine | C_9_H_13_N_3_O_5_ | [M+K]^+^ | 282.0474 | 243.0844 | -4.68 | 12.953 | HILIC | -2.00 | 0.016095 | 1.18611 |
| Arabic acid | C_5_H_10_O_6_ | [M-H]^-^ | 165.0396 | 166.0468 | -5.40 | 13.019 | HILIC | 3.86 | 1.12E-05 | 1.51351 |
| Lactic acid | C_3_H_6_O_3_ | [M-H]^-^ | 89.0240 | 90.0311 | -6.84 | 13.097 | HILIC | -0.51 | 0.006049 | 1.2536 |
| Cytosine | C_4_H_5_N_3_O | [M+H]^+^ | 112.0501 | 111.0428 | -4.27 | 13.200 | HILIC | 3.06 | 0.000183 | 1.33526 |
| Uric acid | C_5_H_4_N_4_O_3_ | [M-H]^-^ | 167.0202 | 168.0275 | -5.07 | 13.280 | HILIC | -0.55 | 0.005851 | 1.28086 |
| Dihydrothymine | C_5_H_8_N_2_O_2_ | [M+H]^+^ | 129.0654 | 128.0581 | -3.79 | 13.424 | HILIC | -0.58 | 0.015786 | 1.34379 |
| p-Aminobenzoic acid | C_7_H_7_NO_2_ | [M+H]^+^ | 138.0544 | 137.0471 | -4.40 | 13.500 | HILIC | -1.99 | 2.36E-09 | 1.79417 |
| Tyrosine | C_9_H_11_NO_3_ | [M+H]^+^ | 182.0804 | 181.0731 | -4.30 | 13.511 | HILIC | -0.66 | 0.002478 | 1.20158 |
| N-Acetylvaline | C_7_H_13_NO_3_ | [M+H]^+^ | 160.0961 | 159.0889 | -4.35 | 13.531 | HILIC | -1.67 | 2.56E-05 | 1.84895 |
| Taurocyamine | C_3_H_9_N_3_O_3_S | [M+H]^+^ | 168.0430 | 167.0357 | -4.52 | 13.549 | HILIC | 1.45 | 0.00262 | 1.30164 |
| 4-Hydroxycinnamic acid | C_9_H_8_O_3_ | [M+H]^+^ | 165.0539 | 164.0466 | -4.74 | 13.586 | HILIC | -0.67 | 0.020156 | 1.09455 |
| Acetic acid | C_2_H_4_O_2_ | [2M+FA-H]^-^ | 165.0397 | 60.0208 | -6.28 | 13.698 | HILIC | -3.45 | 5.91E-08 | 1.80951 |
| 3-Methyladenine | C_6_H_7_N_5_ | [M+H]^+^ | 150.0767 | 149.0695 | -4.64 | 13.817 | HILIC | -0.78 | 6.98E-05 | 1.55135 |
| Pantetheine 4'-phosphate | C_11_H_23_N_2_O_7_PS | [M-H]^-^ | 357.0874 | 358.0947 | -4.63 | 13.871 | HILIC | 4.33 | 0.007581 | 1.16279 |
| Erythrose, 4-phosphate | C_4_H_9_O_7_P | [M+Na]^+^ | 222.9969 | 200.0077 | -4.44 | 14.012 | HILIC | -3.95 | 4.45E-07 | 1.57979 |
| Glutamic acid | C_5_H_9_NO_4_ | [M+H]^+^ | 148.0598 | 147.0525 | -4.53 | 14.013 | HILIC | -4.63 | 7.85E-09 | 1.75792 |
| N-Acetylhistidine | C_8_H_11_N_3_O_3_ | [M+H]^+^ | 198.0866 | 197.0793 | -3.83 | 14.116 | HILIC | -4.79 | 1.57E-06 | 1.73996 |
| Leucyl-glycyl-glycine | C_10_H_19_N_3_O_4_ | [M+H]^+^ | 246.1438 | 245.1365 | -4.44 | 14.118 | HILIC | -1.29 | 4E-05 | 1.51558 |
| Glycyl-l-prolyl-l-alanine | C_10_H_17_N_3_O_4_ | [M+H]^+^ | 244.1281 | 243.1208 | -4.37 | 14.319 | HILIC | -2.22 | 0.000133 | 1.52024 |
| Valine | C_5_H_11_NO_2_ | [M+H]^+^ | 118.0858 | 117.0785 | -4.26 | 14.330 | HILIC | -2.12 | 0.000782 | 1.3062 |
| 4-Acetamidobutanoic acid | C_6_H_11_NO_3_ | [M+H]^+^ | 146.0806 | 145.0733 | -4.11 | 14.347 | HILIC | -1.61 | 1.59E-07 | 1.81853 |
| Gluconic acid | C_6_H_12_O_7_ | [M-H]^-^ | 195.0508 | 196.0581 | -0.94 | 14.421 | HILIC | -2.72 | 9.62E-07 | 1.65247 |
| Glutarylcarnitine | C_12_H_21_NO_6_ | [M+H]^+^ | 276.1431 | 275.1358 | -4.04 | 14.513 | HILIC | -2.61 | 0.000194 | 1.52984 |
| Malic acid | C_4_H_6_O_5_ | [M-H]^-^ | 133.0141 | 134.0214 | -1.22 | 14.759 | HILIC | -1.79 | 1.07E-06 | 1.74219 |
| Peracitol | C_7_H_16_O_7_ | [M-H]^-^ | 211.0814 | 212.0886 | -4.85 | 14.876 | HILIC | 2.05 | 0.003572 | 1.79424 |
| N-Acetylornithine | C_7_H_14_N_2_O_3_ | [M+H]^+^ | 175.1070 | 174.0998 | -3.88 | 14.883 | HILIC | -1.09 | 0.014172 | 1.10807 |
| (2R,3R)-2,3-Dihydroxy-4-(hydroxyamino)-4-oxobutyl dihydrogen phosphate | C_4_H_10_NO_8_P | [M-H]^-^ | 230.0061 | 231.0134 | -4.39 | 15.066 | HILIC | -2.05 | 0.000702 | 1.61042 |
| Alanylglutamine | C_8_H_15_N_3_O_4_ | [M+H]^+^ | 218.1127 | 217.1055 | -3.72 | 15.182 | HILIC | -1.82 | 0.00062 | 1.2032 |
| Citric acid | C_6_H_8_O_7_ | [M-H]^-^ | 191.0190 | 192.0263 | -3.63 | 15.355 | HILIC | -2.52 | 6.86E-08 | 1.89238 |
| Aspartic acid | C_4_H_7_NO_4_ | [M-H]^-^ | 132.0301 | 133.0373 | -1.38 | 15.403 | HILIC | -0.91 | 0.00705 | 1.49548 |
| Asparagine | C_4_H_8_N_2_O_3_ | [M-H]^-^ | 131.0459 | 132.0532 | -2.39 | 15.637 | HILIC | -0.73 | 0.011331 | 1.17951 |
| Glutamine | C_5_H_10_N_2_O_3_ | [M+H]^+^ | 147.0758 | 146.0686 | -3.93 | 15.683 | HILIC | -1.48 | 0.00561 | 1.30324 |
| Pyroglutamic acid | C_5_H_7_NO_3_ | [M+H]^+^ | 130.0494 | 129.0421 | -3.89 | 15.706 | HILIC | -1.36 | 0.002965 | 1.3568 |
| Glycylglycylglycine | C_6_H_11_N_3_O_4_ | [M-H]^-^ | 188.0667 | 189.0740 | -5.05 | 15.943 | HILIC | -1.70 | 0.028735 | 1.19289 |
| Serylserine | C_6_H_12_N_2_O_5_ | [M-H]^-^ | 191.0664 | 192.0736 | -5.15 | 15.974 | HILIC | -2.02 | 0.00051 | 1.32569 |
| Glutaminylserine | C_8_H_15_N_3_O_5_ | [M-H]^-^ | 232.0926 | 233.0999 | -5.61 | 16.076 | HILIC | -1.86 | 0.000382 | 1.36771 |
| 5-(2-Hydroxyethyl)-4-methylthiazole | C_6_H_9_NOS | [M+H]^+^ | 144.0472 | 143.0399 | -4.25 | 16.128 | HILIC | -2.40 | 0.000467 | 1.2392 |
| L-Histidine trimethylbetaine | C_9_H_15_N_3_O_2_ | [M+H]^+^ | 198.1231 | 197.1158 | -3.39 | 16.206 | HILIC | -1.49 | 0.000361 | 1.33064 |
| L-Sorbose | C_6_H_12_O_6_ | [M-H]^-^ | 179.0553 | 180.0626 | -4.30 | 16.321 | HILIC | -0.68 | 0.031364 | 1.05189 |
| Diethyl dicarbonate | C_6_H_10_O_5_ | [M-H]^-^ | 161.0450 | 162.0522 | -3.59 | 16.338 | HILIC | -0.82 | 0.001183 | 1.26805 |
| Dimethyl-L-arginine | C_8_H_18_N_4_O_2_ | [M+H]^+^ | 203.1496 | 202.1423 | -3.42 | 16.510 | HILIC | -1.77 | 0.000919 | 1.11982 |
| L-Targinine | C_7_H_16_N_4_O_2_ | [M+H]^+^ | 189.1339 | 188.1266 | -3.81 | 16.898 | HILIC | -2.33 | 0.002644 | 1.2296 |
| Methylhistidine | C_7_H_11_N_3_O_2_ | [M+H]^+^ | 170.0918 | 169.0845 | -3.75 | 16.970 | HILIC | -2.98 | 3.28E-08 | 1.66256 |
| Histidine | C_6_H_9_N_3_O_2_ | [M-H]^-^ | 154.0617 | 155.0689 | -3.46 | 17.091 | HILIC | -1.48 | 0.001501 | 1.374 |
| 4-Hydroxy-L-threonine | C_4_H_9_NO_4_ | [M+H]^+^ | 136.0613 | 135.0540 | 6.30 | 17.176 | HILIC | -1.02 | 0.017307 | 1.09368 |
| Adenosine monophosphate | C_10_H_14_N_5_O_7_P | [M-H]^-^ | 346.0541 | 347.0614 | -4.96 | 17.275 | HILIC | -1.77 | 1.2E-05 | 1.82379 |
| Uridine monophosphate | C_9_H_13_N_2_O_9_P | [M-H]^-^ | 323.0271 | 324.0344 | -4.67 | 17.321 | HILIC | -1.94 | 1.45E-06 | 1.87532 |
| Inosine-5'-monophosphate | C_10_H_13_N_4_O_8_P | [M-H]^-^ | 347.0377 | 348.0449 | -6.22 | 17.534 | HILIC | -1.56 | 0.000376 | 1.76441 |
| Lysine | C_6_H_14_N_2_O_2_ | [M+H]^+^ | 147.1122 | 146.1049 | -4.01 | 17.702 | HILIC | -1.71 | 0.009272 | 1.12396 |
| Ornithine | C_5_H_12_N_2_O_2_ | [M+H]^+^ | 133.0966 | 132.0893 | -4.04 | 17.779 | HILIC | -2.62 | 4.53E-05 | 1.4394 |
| D-Ribulose 5-phosphate | C_5_H_11_O_8_P | [M-H]^-^ | 229.0102 | 230.0175 | -7.12 | 17.968 | HILIC | -2.64 | 3.91E-05 | 1.61203 |
| Glucose 6-phosphate | C_6_H_13_O_9_P | [M-H]^-^ | 259.0207 | 260.0279 | -6.83 | 18.030 | HILIC | -1.03 | 0.018857 | 1.27878 |
| Guanosine monophosphate | C_10_H_14_N_5_O_8_P | [M-H]^-^ | 362.0480 | 363.0553 | -7.39 | 18.035 | HILIC | -1.06 | 0.00184 | 1.56326 |
| (16alpha,17beta)-16-Hydroxyestra-1,3,5(10)-triene-3,17-diyl dihexanoate | C_30_H_44_O_5_ | [M-H]^-^ | 483.3102 | 484.3175 | -2.91 | 7.372 | RP-C18 | 1.03 | 0.005795 | 2.40182 |
| Dibutyl phthalate | C_16_H_22_O_4_ | [M+H]^+^ | 279.1581 | 278.1508 | -3.73 | 8.410 | RP-C18 | -0.46 | 0.04001 | 1.22161 |
| Androstane | C_19_H_32_ | [M-H]^-^ | 259.2419 | 260.2492 | -4.81 | 9.324 | RP-C18 | 0.62 | 0.029889 | 1.6633 |
| Docosahexaenoic acid | C_22_H_32_O_2_ | [M-H]^-^ | 327.2321 | 328.2394 | -2.54 | 10.370 | RP-C18 | 0.85 | 0.046142 | 1.83197 |
| Palmitic acid | C_16_H_32_O_2_ | [M-H]^-^ | 255.2318 | 256.2391 | -4.46 | 10.504 | RP-C18 | 0.43 | 0.040523 | 1.5059 |
| Lysidine | C_4_H_8_N_2_ | [M+H]^+^ | 85.0755 | 84.0683 | -5.83 | 10.807 | RP-C18 | -3.17 | 0.013268 | 1.15865 |
| Styrene | C_8_H_8_ | [M+H]^+^ | 105.0695 | 104.0622 | -3.58 | 10.824 | RP-C18 | 0.60 | 1.36E-05 | 1.91352 |
| 9,12-Octadecadienal | C_18_H_32_O | [M+H]^+^ | 265.2517 | 264.2444 | -3.62 | 10.864 | RP-C18 | -0.67 | 0.000216 | 1.71234 |
| Phosphonoacetate | C_2_H_4_O_5_P | [M+H]^+^ | 139.9874 | 138.9802 | 3.73 | 11.019 | RP-C18 | 0.30 | 0.002664 | 1.28053 |

**Table S9. Concentration-response standard curve of neurotransmitters and tricarboxylic acid cycle metabolite.**

| Compound | Standard Curve | r^2^ |
| --- | --- | --- |
| Homocysteine | *Y* = 2066.66695*X* + 7077.09388 | 0.999 |
| Melatonin | *Y* = 1.16685e5*X* - 8.42101e4 | 0.999 |
| Tryptamine | *Y* = 1.31683e5*X* - 4.23620e4 | 0.999 |
| Picolinic acid | *Y* = 8.85275e4*X* - 3.81519e4 | 0.999 |
| Skatole | *Y* = 4958.19992*X* + 7030.01982 | 0.998 |
| S-adenosyl homocysteine | *Y* = 28130.37933*X* - 23756.06995 | 0.999 |
| Kynurenine | *Y* = 9.87040e4*X* - 28930.43208 | 0.999 |
| 3-Hydroxykynurenine | *Y* = 9.72592e4*X* + 3939.07075 | 0.999 |
| Nicotinic acid | *Y* = 6.91227e4*X* + 1.02229e5 | 0.999 |
| Urea | *Y* = 4244.43365*X* + 2.07059e6 | 0.991 |
| kynurenic acid | *Y* = 1890.32834*X* + 3.79579e5 | 0.999 |
| 5-Hydroxyindole-3-acetic acid | *Y* = 1154.64082*X* + 1.71583e5 | 0.995 |
| Adenosine diphosphate | *Y* = 83.59174*X* + 2861.83661 | 0.995 |
| NAD^+^ | *Y* = 576.99490*X* - 6290.82129 | 0.999 |
| Citric acid | *Y* = 3788.01304*X* + 5.87381e4 | 0.999 |
| Malic acid | *Y* = 10986.10012*X* + 28304.81086 | 0.999 |
| Pyruvic acid | *Y* = 73.51366*X* + 9685.91018 | 0.999 |
| Lactic acid | *Y* = 526.97707*X* + 4.57576e5 | 0.993 |
| Succinic acid | *Y* = 6776.60484*X* + 2.81272e5 | 0.999 |
| 5'-Adenylic acid | *Y* = 3635.47026*X* - 11917.47692 | 1.000 |
| NADH | *Y* = 72.97602*X* + 628.63071 | 1.000 |
| Methionine | *Y* = 26259.94036*X* + 14377.84489 | 0.999 |
| Serotonin | *Y* = 9.22757e4*X* + 6.88032e4 | 0.998 |
| Indole-3-acetic acid | *Y* = 6.47018e4*X* + 24843.84966 | 0.999 |
| Glycine | *Y* = 738.85513*X* + 27345.22180 | 0.998 |
| Alanine | *Y* = 7515.32377*X* + 1.50012e5 | 0.999 |
| Glutamine | *Y* = 1.23547e5*X* + 1.99184e6 | 0.985 |
| Glutamic acid | *Y* = 3.24120e4*X* + 9.81264e5 | 0.992 |
| Cysteine | *Y* = 2884.60364*X* + 9278.00943 | 0.998 |
| Choline | *Y* = 6.09836e4*X* + 1.45405e7 | 0.990 |
| Dopamine | *Y* = 5.81550e4*X* + 8.28005e4 | 0.999 |
| Citrulline | *Y* = 5.29692e4*X* + 5.09104e5 | 0.996 |
| Arginine | *Y* = 12042.26062*X* + 1.46454e6 | 0.995 |
| Lysine | *Y* = 6.41017e4*X* + 2.49219e6 | 0.996 |
| Leucine | *Y* = 1.49353e5*X* + 1.95025e6 | 0.991 |
| Ornithine | *Y* = 3.59968e4*X* + 3.16124e5 | 1.000 |
| Adrenaline | *Y* = 3272.96541*X* + 3.68804e5 | 0.999 |
| Serine | *Y* = 12068.60233*X* + 4.13133e5 | 0.999 |
| Threonine | *Y* = 9140.51987*X* + 3.00151e5 | 0.999 |
| Aspartic acid | *Y* = 8692.66898*X* + 3.40473e5 | 0.985 |
| Asparagine | *Y* = 9420.46853*X* + 7.93763e4 | 0.987 |
| γ-Aminobutyric acid | *Y* = 16224.75050*X* + 3.94790e5 | 0.992 |
| Acetylcholine | *Y* = 1.14498e6*X* + 4.44158e5 | 0.999 |
| Histamine | *Y* = 5.50542e5*X* + 5.53590e4 | 0.986 |
| Levodopa | *Y* = 4.34334e4*X* + 5.02822e4 | 0.999 |
| Taurine | *Y* = 3419.13214*X* + 3.58478e4 | 0.987 |
| Proline | *Y* = 6.68087e4*X* + 1.37518e6 | 0.982 |
| Phenylalanine | *Y* = 3.06972e5*X* + 1.04735e6 | 0.999 |
| Histidine | *Y* = 9.70259e4*X* + 7.96920e5 | 0.999 |

**Table S10. List of primer sequences for the target gene.**

| Gene symbol | Forward primer sequence (5′–3′) | Reverse primer sequence (5′–3′) |
| --- | --- | --- |
| *Adipoq* | ACAATGGCACACCAGGCCGTG | GAAGCCCCGTGGCCCTTCAG |
| *Lep* | CCCTCATCAAGACCATTGTCACC | CCAAGCCAGTGACCCTCTGC |
| *Fabp4* | CACCGCAGACGACAGGAAGG | TCCACCACCAGCTTGTCACCA |
| *Pck1* | CTCCGAGGAGGAGTACGGGCA | TCCTGGCCACATCTCGAGGG |
| *Aldob* | GTGGTGGGCATCAAGTTGGACC | GAGCACAGCGTTCAGAGAGGCC |
| *G6pc1* | CCTCTGGGTGGCAGTGGTCGG | CCACCAATACGGGCGTTGTCC |
| *Hnf4a* | GGTTTAGCCGACAATGTGTGG | TCCCGCTCATTTTGGACAGC |
| *Fbp1* | CACCGCGATCAAAGCCATCT | CCAGTCACATTGGTTGAGCCA |
| *Slc4a1* | TGGGGTCGCCCACATCTAT | AGGAAGGTGCCTTTTGAGAAGA |
| *Obp2a* | GGCTGTCCTGAAGGCTCAGGAA | GGGTACCATTGTGTACCATGGCC |
| *Car1* | CGACCATGGCTCTGAGCACACC | TGCCAGGCCATCAGCCTTGG |
| *Abca1* | GTTACGGCAGATCAAGCATCC | TGGAAGGGACAAATTGTGCTG |
| *Pik3ap1* | AAATGACTTGTGATGATGAGCCA | GCTCCGTGTCGGTTACTGA |
| *Akt1* | AGAAGAGACGATGGACTTCCG | TCAAACTCGTTCATGGTCACAC |
| *Nos3* | GCCCTGGCCGGGGAGACTTCC | GGGCAGCACATCAAAGCGGCC |
| *Gpx4* | CGACGGGCACATGGTCTGCC | GGTTGCAGGGGAAGGCCAGG |
| *Cxcl9* | CAGCACCAGCCGAGGCACGA | GCCGAGTCCGGATCTAGGCAGG |
| *Ly86* | GCCGGCCCTGTCAATAACCC | GGCACAAGCCACAGTAGCACGG |
| *Cd68* | CCCAACAAAACCAAGGTCCAGG | GGACACATTGTATTCCACCGCC |
| *Ccl6* | CCCCACCAGTGGTGGGTGCA | TGGGCCTTGCTTCAGGGTGC |
| *Itgax* | CTGGATAGCCTTTCTTCTGCTG | GCACACTGTGTCCGAACTCA |
| *Cst7* | CTGACACCCATGGGGCACGC | GCGGCCTTAAGCACTCCTGGG |
| *Wfdc17* | GGTGGCTTTGATCACTGTGGGG | GGCTTGGGACAAGCTCCAGGTT |
| *Gusb* | CGGGAAGCAATCCTGCCACG | CCTCAAAGGGGAGGTGACCTCCC |
| *Elk1* | CCAGCACCCGCCTCAGGGAG | GTTTGGGGCCTCTGGTGGGG |
| *Csf3r* | GGGCTGGGAGCCTGCACCCTG | GGACAGGGTCCCCCAGGCGG |
| *Fxyd2* | GGTGGTACCTGGGTGGCAGTGC | ATGATGAGGAGGCCCACGACGA |
| *Atp2a1* | GAAGGCGAAGAAACCGTCACT | TAGCGTTCTCTGCATTCCGTT |
| *Aspg* | GACCAGAGCGGCGGCTGCTG | CTTCAGGACGGCAGCCAGGCC |
| *Asns* | CAGACCGGAGGATTGGCTGCC | TCGGGGCTGTCCTCCATGCC |
| *Serpina12* | GACCCAGGCACCGTGATGATTC | AGGCCATGTCGTACAAGCCCC |
| *Hes1* | GCACCTCCGGAACCTGCAGCGG | ACTCGCTGAAGCCGGCGCGG |
| *Ugt1a9* | GGGGTGACCCTGAATGTCCTTG | TGAAGGCTGGAGAGGCGCAT |
| *Svbp* | CCAGCCTTCAGAGTGGAGAAGGC | CTGCTGCTGCTCCAGCTCCG |
| *Actb* | AGAGGGAAATCGTGCGTGAC | CAATAGTGATGACCTGGCCGT |
| *Gapdh* | AGGTCGGTGTGAACGGATTTG | GGGGTCGTTGATGGCAACA |
| *Slc2a2* | TGTGTGAGGATGACCTGC | CGAGGAAGGAATCGGTTTAAG |
| *Slc25a18* | GCTCCTCAGATTGTATCACG | GTCACTCCACATCTGTGTT |
| *Tnf* | TCCCCAAAGGGATGAGAAGTT | GAGGAGGTTGACTTTCTCCTGG |
| *Il6* | CTTCTTGGGACTGATGCTGGT | CACAACTCTTTTCTCATTTCCACG |
| *Il12a* | GCATGTGTCAATCACGCTACC | ATGTCATCTGTGGTCTTCAGCAG |
| *Il12b* | GGACATCATCAAACCAGACCC | ACGCACCTTTCTGGTTACACC |
| *Nr1h2* | CAACAAACGATCTTTCTCCGAC | GGCAAAGTCCACAATCTCCTG |
| *Pparg* | GCTTGTGAAGGATGCAAGGG | GATATCACTGGAGATCTCCGCC |
| *Ifng* | CTCAAGTGGCATAGATGTGGAAG | GACCTCAAACTTGGCAATACTCA |
| *Ccl2* | CCCTACTATTCCTGATGGCACT | CTATGAGAAACCCACCACATCTG |
| *Il1b* | GGGCCTCAAAGGAAAGAATCT | GAGGTGCTGATGTACCAGTTGG |

**Table S11. Animal allocation and attrition**

| Group | Abbreviation | Sex | Treatment | Initial | Mortality | Excluded | Final n for behavioral analysis |
| --- | --- | --- | --- | --- | --- | --- | --- |
| Wild-type control | WT | Male | Vehicle | 15 | 0 | 0 | 15 |
| 5xFAD model | AD | Male | Vehicle | 16 | 0 | 0 | 16 |
| HLJDD high-dose | H-H | Male | HLJDD,  116 mg·kg⁻¹·d⁻¹ | 16 | 0 | 0 | 16 |
| HLJDD low-dose | H-L | Male | HLJDD,  58 mg·kg⁻¹·d⁻¹ | 11 | 0 | 0 | 11 |
| Donepezil | Don | Male | Donepezil,  1 mg·kg⁻¹·d⁻¹ | 10 | 0 | 0 | 10 |

**Table S12. Assay-specific biological sample sizes**

| Assay | Sample | WT | AD | H-H | H-L | Don |
| --- | --- | --- | --- | --- | --- | --- |
| Behavioral tests | Whole animal | 15 | 16 | 16 | 11 | 10 |
| RNA sequencing | Brain, liver | 3 | 3 | 3 | — | — |
| DIA proteomics | Brain, liver | 5 | 5 | 5 | — | — |
| Phosphoproteomics | Brain, liver | 5 | 5 | 5 | — | — |
| Untargeted metabolomics | Brain, liver, serum | 6 | 6 | 6 | — | — |
| qRT-PCR | Brain, liver | 3 | 3 | 3 | — | — |
| Biochemical assays | Brain, liver, serum | 6 | 6 | 6 | — | — |
| H&E staining | Brain | 3 | 3 | 3 | 3 | 3 |
| Congo red staining | Brain | 3 | 3 | 3 | 3 | 3 |
| Aβ immunohistochemistry | Brain | 3 | 3 | 3 | 3 | 3 |
| PS1 immunohistochemistry | Brain | 3 | 3 | 3 | 3 | 3 |
| PPAR-γ immunofluorescence | Brain | 3 | 3 | 3 | — | — |
| PPAR-γ immunohistochemistry | Liver | 3 | 3 | 3 | — | — |
| NF-κB immunohistochemistry | Brain; liver | 3 | 3 | 3 | — | — |

**Table S13. Randomization, blinding, batch allocation, and sample-size rationale**

| Design feature | Procedure |
| --- | --- |
| Randomization | Animals were allocated to the experimental groups using a random-number method. |
| Behavioral testing and analysis | Behavioral testing was jointly conducted by two investigators, one of whom was blinded to group allocation. All behavioral assays were performed according to predefined standardized protocols under consistent experimental conditions. Behavioral parameters were objectively recorded and quantified using automated tracking software. |
| Image quantification blinding | Histological and immunofluorescence images were coded before quantification, and investigators performing image quantification were blinded to group allocation. |
| Batch allocation | Samples from different experimental groups were distributed across processing and analytical batches to minimize potential batch effects. When multiple batches were required, samples from the different experimental groups were represented within each batch. |
| Mortality and exclusions | No animal deaths or exclusions occurred throughout the study. |
| Experimental unit | The individual mouse was considered the experimental unit. For histological and imaging analyses, measurements from multiple sections or microscopic fields obtained from the same mouse were averaged to generate a single value per animal and were not treated as independent biological replicates. |
| Sample-size rationale | No formal a priori power calculation was performed. Sample sizes were determined based on preliminary experimental observations, sample sizes commonly used in previous studies employing the 5xFAD model, the anticipated variability of the respective experimental endpoints, analytical feasibility, the planned allocation of animals across multi-omics and validation assays, and animal-welfare considerations. |
